# Genome-wide association study identifies Sjögren’s risk loci with functional implications in immune and glandular cells

**DOI:** 10.1038/s41467-022-30773-y

**Published:** 2022-07-27

**Authors:** Bhuwan Khatri, Kandice L. Tessneer, Astrid Rasmussen, Farhang Aghakhanian, Tove Ragna Reksten, Adam Adler, Ilias Alevizos, Juan-Manuel Anaya, Lara A. Aqrawi, Eva Baecklund, Johan G. Brun, Sara Magnusson Bucher, Maija-Leena Eloranta, Fiona Engelke, Helena Forsblad-d’Elia, Stuart B. Glenn, Daniel Hammenfors, Juliana Imgenberg-Kreuz, Janicke Liaaen Jensen, Svein Joar Auglænd Johnsen, Malin V. Jonsson, Marika Kvarnström, Jennifer A. Kelly, He Li, Thomas Mandl, Javier Martín, Gaétane Nocturne, Katrine Brække Norheim, Øyvind Palm, Kathrine Skarstein, Anna M. Stolarczyk, Kimberly E. Taylor, Maria Teruel, Elke Theander, Swamy Venuturupalli, Daniel J. Wallace, Kiely M. Grundahl, Kimberly S. Hefner, Lida Radfar, David M. Lewis, Donald U. Stone, C. Erick Kaufman, Michael T. Brennan, Joel M. Guthridge, Judith A. James, R. Hal Scofield, Patrick M. Gaffney, Lindsey A. Criswell, Roland Jonsson, Per Eriksson, Simon J. Bowman, Roald Omdal, Lars Rönnblom, Blake Warner, Maureen Rischmueller, Torsten Witte, A. Darise Farris, Xavier Mariette, Marta E. Alarcon-Riquelme, PRECISESADS Clinical Consortium, Caroline H. Shiboski, Sjögren’s International Collaborative Clinical Alliance (SICCA), Marie Wahren-Herlenius, Wan-Fai Ng, UK Primary Sjögren’s Syndrome Registry, Kathy L. Sivils, Indra Adrianto, Gunnel Nordmark, Christopher J. Lessard

**Affiliations:** 1 Genes and Human Disease Research Program, Oklahoma Medical Research Foundation, Oklahoma City, OK USA; 2 Arthritis and Clinical Immunology Research Program, Oklahoma Medical Research Foundation, Oklahoma City, OK USA; 3 Department of Clinical Science, University of Bergen, Bergen, Norway; 4 NGS Core Laboratory, Oklahoma Medical Research Foundation, Oklahoma City, OK USA; 5 Salivary Disorder Unit, National Institute of Dental and Craniofacial Research, Bethesda, MD USA; 6 Center for Autoimmune Diseases Research (CREA), Universidad del Rosario, Bogotá, Colombia; 7 Department of Oral Surgery and Oral Medicine, Faculty of Dentistry, University of Oslo, Oslo, Norway; 8 Department of Health Sciences, Kristiania University College, Oslo, Norway; 9 Department of Medical Sciences, Rheumatology and Science for Life Laboratory, Uppsala University, Uppsala, Sweden; 10 Department of Rheumatology, Faculty of Medicine and Health, Örebro University, Örebro, Sweden; 11 Department of Rheumatology and Immunology, Hannover Medical School, Hannover, Germany; 12 Department of Rheumatology and Inflammation Research, Sahlgrenska Academy at University of Gothenburg, Gothenburg, Sweden; 13 Department of Rheumatology, Haukeland University Hospital, Bergen, Norway; 14 Department of Internal Medicine, Clinical Immunology Unit, Stavanger University Hospital, Stavanger, Norway; 15 Section for Oral and Maxillofacial Radiology, Department of Clinical Dentistry, Medical Faculty, University of Bergen, Bergen, Norway; 16 Rheumatology Unity, Department of Medicine, Karolinska University Hospital, Karolinska Institutet, Stockholm, Sweden; 17 Academic Specialist Center, Center for Rheumatology and Studieenheten, Stockholm Health Services, Region Stockholm, Sweden; 18 Translational Sciences, The Janssen Pharmaceutical Companies of Johnson & Johnson, Spring House, PA USA; 19 Rheumatology, Department of Clinical Sciences Malmö, Lund University, Malmö, Sweden; 20 Instituto de Biomedicina y Parasitología López-Neyra, Consejo Superior de Investigaciones Científicas (CSIC), Granada, Spain; 21 Université Paris-Saclay, Assistance Publique–Hôpitaux de Paris (AP-HP), Hôpital Bicêtre, Institut National de la Santé et de la Recherche Médicale (INSERM) UMR1184, Le Kremlin Bicêtre, France; 22 Department of Rheumatology, Stavanger University Hospital, Stavanger, Norway; 23 Department of Rheumatology, University of Oslo, Oslo, Norway; 24 Department of Pathology, Haukeland University Hospital, Bergen, Norway; 25 Department of Medicine, Russell/Engleman Rheumatology Research Center, University of California San Francisco, San Francisco, California USA; 26 Genyo, Center for Genomics and Oncological Research, Pfizer/University of Granada/Andalusian Regional Government, Granada, Spain; 27 Department of Rheumatology, Skåne University Hospital, Malmö, Sweden; 28 Medical Affairs, Jannsen-Cilag EMEA (Europe/Middle East/Africa), Beerse, Belgium; 29 Division of Rheumatology, Cedars-Sinai Medical Center, Los Angeles, CA USA; 30 David Geffen School of Medicine, University of California Los Angeles, Los Angeles, CA USA; 31 Hefner Eye Care and Optical Center, Oklahoma City, OK USA; 32 Oral Diagnosis and Radiology Department, University of Oklahoma College of Dentistry, Oklahoma City, OK USA; 33 Department of Oral and Maxillofacial Pathology, University of Oklahoma College of Dentistry, Oklahoma City, OK USA; 34 Department of Ophthalmology, Dean McGee Eye Institute, University of Oklahoma Health Sciences Center, Oklahoma City, OK USA; 35 Department of Medicine, University of Oklahoma Health Sciences Center, Oklahoma City, OK USA; 36 Department of Oral Medicine/Oral & Maxillofacial Surgery, Atrium Health Carolinas Medical Center, Charlotte, NC USA; 37 Department of Otolaryngology/Head and Neck Surgery, Wake Forest University School of Medicine, Winston-Salem, NC USA; 38 Department of Pathology, University of Oklahoma Health Sciences Center, Oklahoma City, OK USA; 39 US Department of Veterans Affairs Medical Center, Oklahoma City, OK USA; 40 Institute of Human Genetics (IHG), University of California San Francisco, San Francisco, CA USA; 41 Genomics of Autoimmune Rheumatic Disease Section, National Human Genome Research Institute, NIH, Bethesda, MD USA; 42 Department of Biomedical and Clinical Sciences, Division of Inflammation and Infection, Linköping University, Linköping, Sweden; 43 Rheumatology Department, University Hospital Birmingham NHS Foundation Trust, Birmingham, UK; 44 Rheumatology Research Group, Institute of Inflammation & Ageing, University of Birmingham, Birmingham, UK; 45 Rheumatology Department, Milton Keynes University Hospital, Milton Keynes, UK; 46 Rheumatology Department, The Queen Elizabeth Hospital, Woodville, South Australia; 47 University of Adelaide, Adelaide, South Australia; 48 Department of Orofacial Sciences, University of California San Francisco, San Francisco, CA USA; 49 Translational and Clinical Research Institute, Newcastle University, Newcastle upon Tyne, UK; 50 NIHR Newcastle Biomedical Centre and NIHR Newcastle Clinical Research Facility, Newcastle upon Tyne Hospitals NHS Foundation Trust, Newcastle upon Tyne, UK; 51 Center for Bioinformatics, Department of Public Health Sciences, Henry Ford Health System, Detroit, MI USA

**Keywords:** Rheumatic diseases, Rheumatic diseases, Genome-wide association studies, Genetic predisposition to disease

## Abstract

Sjögren’s disease is a complex autoimmune disease with twelve established susceptibility loci. This genome-wide association study (GWAS) identifies ten novel genome-wide significant (GWS) regions in Sjögren’s cases of European ancestry: *CD247*, *NAB1*, *PTTG1-MIR146A*, *PRDM1-ATG5*, *TNFAIP3*, *XKR6*, MAPT-*CRHR1*, *RPTOR-CHMP6-BAIAP6*, *TYK2*, *SYNGR1*. Polygenic risk scores yield predictability (AUROC = 0.71) and relative risk of 12.08. Interrogation of bioinformatics databases refine the associations, define local regulatory networks of GWS SNPs from the 95% credible set, and expand the implicated gene list to >40. Many GWS SNPs are eQTLs for genes within topologically associated domains in immune cells and/or eQTLs in the main target tissue, salivary glands.

## Abstract (web summary)

The genetic architecture underlying Sjögren’s syndrome is not fully understood. Here, the authors perform a genome-wide association study to identify 10 new genetic risk regions, implicating genes involved in immune and salivary gland function.

## Introduction

Sjögren’s disease is a chronic autoimmune disease characterized by the existence of autoantibodies to ribonuclear proteins, Ro-52 and Ro-60, as well as focal lymphocytic infiltration of the exocrine glands resulting in hypofunction and dryness^1–8^. Extraglandular manifestations can also present as leukocytoclastic vasculitis, inflammatory arthritis, pulmonary, and/or neurological dysfunction, as well as a 7-to-19-fold increased risk of lymphoma^9–11^. The etiology of Sjögren’s remains unclear, but evidence suggests that Sjögren’s develops in genetically susceptible individuals who were exposed to unknown environmental conditions^12–14^. Polygenic liability modeling estimated the familial transmission for Sjögren’s, e.g., heritability and shared environmental contributions, to be 0.54^15^.

Studies of Sjögren’s genetics have been largely limited to familial aggregation and candidate gene studies^12^. In 2013, the Sjögren’s Genetics Network (SGENE) published the first large-scale genomic study of Sjögren’s of European ancestry using the ImmunoChip 1.0 array^16^. It identified six novel genome-wide significant (GWS; *P*_GWAS_ < 5 × 10^−08^) regions of association, identified several additional suggestive regions of association, and replicated previously established regions^16^. In the same issue of *Nature Genetics*, Li et al. published the first genome-wide association study (GWAS) identifying *GTF2IRD1-GTF2I* as a GWS region of association in Sjögren’s of Han Chinese ancestry^17^. Later, Zhao et al. leveraged ImmunoChip data from European and East Asian populations to further characterize the *GTF2IRD1-GTF2I* locus in systemic lupus erythematosus (SLE) and Sjögren’s, discovering a missense mutation in Neutrophil cytosolic factor 1 (*NCF1*) (pArg90His) that decreases reactive oxygen species production and predisposes individuals to Sjögren’s and other autoimmune diseases^18^. Also in 2017, the Sjögren’s International Collaborative Clinical Alliance (SICCA) reported a GWAS of Sjögren’s of European and Asian ancestry that uncovered ancestry-specific heterogeneity between genetic associations, replicated previously established associations, and identified several suggestive regions of association but, due to the small European case-control cohort, did not identify new GWS associations^19^. To date, only 12 loci (9 in European populations) are established as GWS associations with Sjögren’s (Supplementary Data 1)^12,16–24^; at least ten times fewer loci than related autoimmune diseases, such as SLE and rheumatoid arthritis (RA)^25,26^.

**Fig. 1:**
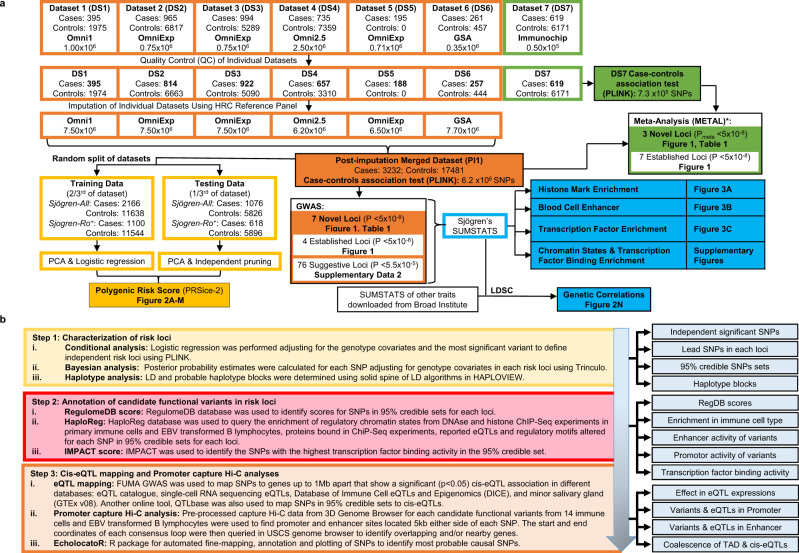
Schematic of the Sjögren’s GWAS and Bioinformatic Workflow. **a** Workflow of the six genotyped datasets (DS1–6) and one ImmunoChip dataset (DS7) used in this study, including the number of cases, controls, and SNPs included in each dataset pre- and post-quality control, and after whole-genome imputation. The post-imputation merged dataset (PI1) containing DS1–6 was used to perform the SNP-Sjögren’s single marker trait analysis (orange), the polygenic risk score (PRS) analysis (yellow), the genetic correlation analyses (blue), and the epigenetic enrichment analyses (blue). Meta-analysis was performed using the genotyped PI1 merged dataset and DS7 ImmunoChip dataset (green) merged using the DS7 genotyping platform. See Supplementary Data 1 for detailed information for each dataset. **b** Statistical and bioinformatic analysis workflow applied to each novel risk locus to identify and predict functionality of likely functional SNPs.

Defining the genetic risk of Sjögren’s will provide important insights into the dysregulated molecular mechanisms that influence disease pathogenesis and promote the development of new therapeutic approaches to improve early diagnosis and treatment. To address the current gap in Sjögren’s genetics, we performed the largest GWAS to date in Sjögren’s of European ancestry, resulting in the identification of ten novel GWS associations (Fig. 1a). Using genotyped SNPs, we also assessed the genetic correlation with related autoimmune diseases and the ability of these SNPs to predict disease using polygenic risk score (PRS) analyses. Last, we performed a deep analysis of bioinformatic data to predict the functionality of the most likely functional SNPs in each locus (Fig. 1b). Our approach, which searched for a coalescence of available cell type-specific expression quantitative trait loci (eQTLs) and mapped topologically associated domain (TAD) interactions with enrichment of epigenetic marks indicative of gene regulation, identified several likely functional SNPs from each locus for future mechanistic investigation^27–30^. Further, mapping TADs that interacted with genomic regions carrying risk SNPs revealed several extended regulatory networks that likely modulate the expression of >40 additional genes up or downstream of the index gene.

## Results

### Genome-wide association study of Sjögren’s of European ancestry

**Fig. 2:**
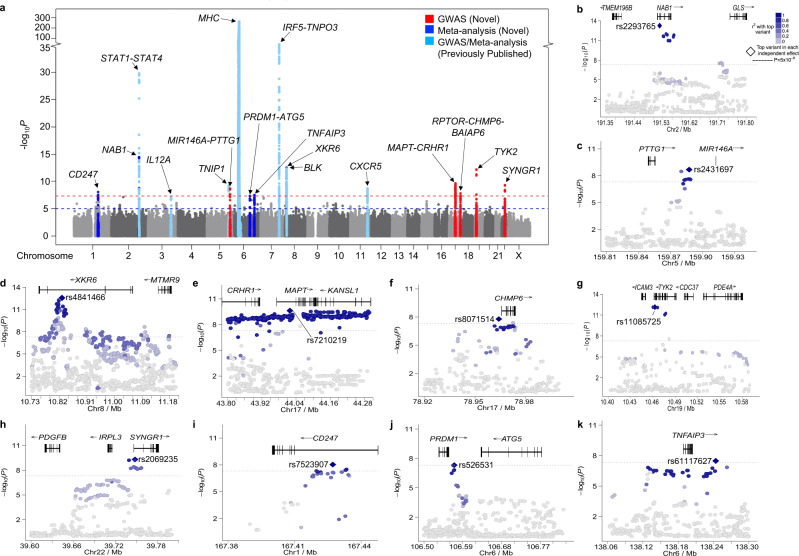
Summary of the SNP-Sjögren associations in a European population. **a** Manhattan plot shows the summary data from the meta-analysis of the 7.3 × 10^5^ SNPs shared between the GWAS and ImmunoChip dataset (Supplementary Data 1) after imputation. The −log_10_(*P*) for each variant is plotted according to chromosome and base pair position. A total of seven novel loci (indicated in red) exceeded genome-wide significance (GWS) of *P*_GWAS_ < 5 × 10^−8^ (red dashed line). Three additional novel loci (indicated in royal blue) exceed GWS after meta-analysis (*P*_*META*_ < 5 × 10^−8^). Several previously established loci were replicated (indicated in light blue). The suggestive GWAS and meta-analysis threshold (*P*_Suggestive_ < 5 × 10^−5^) is indicated by the blue dashed line. **b**–**h** Logistical regression analysis was performed on GWAS dataset PI1 after imputation, identifying the top SNP associations (e.g., index SNPs) of the novel GWS regions of association: *NAB1* (**b**), *PTTG1-MIR146A* (**c**), *XKR6* (**d**), *MAPT-CRHR1* (**e**), *RPTOR-CHMP6-BAIAP6* (**f**), *TYK2* (**g**), *SYNGR1* (**h**). **i**–**k** Logistical regression analysis was performed after meta-analysis of the GWAS and ImmunoChip data, identifying the top SNP associations (e.g., index SNPs) of the novel GWS regions of association: *CD247* (**i**), *PRDM1-ATG5* (**j**), *TNFAIP3* (**k**).

A GWAS was performed on 3232 Sjögren’s cases and 17,481 population controls of European ancestry remaining after quality control (Supplementary Fig. 1A–G; Supplementary Data 2). Inflation in the test statistic for the 101,574 SNPs in common between the arrays was *λ* = 1.15 and was reduced to *λ* = 1.10 when the previously established regions were removed (Supplementary Fig. 1E, F). Whole-genome imputation increased the number of variants tested for single-marker SNP-Sjögren’s association from 101,574 to 6,257,359 polymorphisms tested. In total, seven novel regions exceeded GWS (*P*_GWA*S*_ < 5 × 10^−8^), while 74 additional regions reached suggestive association (*P*_Suggestive_ < 5 × 10^−5^) (Fig. 2a–h; Table 1; Supplementary Fig. 1G; Supplementary Data 3). Four of the nine previously established regions in Sjögren’s of European ancestry were replicated at GWS threshold: major histocompatibility complex (MHC) region, including the previously associated MICA*008, *STAT1-STAT4*, *TNIP1*, and *IRF5-TNPO3* (Supplementary Fig. 1G, Supplementary Data 1). Previously associated *IL12A*, *BLK*, and *CXCR5* loci reached suggestive association threshold.

**Table 1:** Summary results of the GWAS and meta-analysis of Sjögren’s of European Ancestry.

Index gene	Other disease associations^a^					GWAS			Immunochip			Meta
SNP rsID	Chr	Base pair	min^b^	maf^c^	MAF^d^	O/I^e^	OR (95%CI)	*P* value	O/I	OR (95%CI)	*P* value	*P* value
Novel significant loci by GWAS (*P* < 5 × 10^−8^):												
**NAB1**		IIM, MS, PBC, RA, SLE, SSc										
rs2293765	2	191520845	C	A	0.41	O	1.24 (1.17–1.32)	5.53 × 10^−14^	–	–	–	–
rs11900804	2	191533767	C	T	0.42	I	1.22 (1.16–1.30)	2.44 × 10^−12^	–	–	–	–
rs2192008	2	191545147	A	G	0.42	I	1.23 (1.16–1.30)	1.37 × 10^−12^	I	1.22 (1.07–1.40)	2.71 × 10^−03^	2.18 × 10^−14^
rs744600	2	191564757	G	T	0.38	I	1.22 (1.16–1.30)	2.89 × 10^−12^	I	1.27 (1.11–1.45)	4.53 × 10^−03^	6.07 × 10^−15^
rs10202868	2	191563991	C	T	0.25	I	0.88 (0.83–0.95)	5.49 × 10^−4^	O	0.89 (0.76–1.04)	0.16	2.24 × 10^−4^
**PTTG1-MIR146A**		SLE										
rs2910194	5	159844175	T	C	0.49	I	1.11 (1.05–1.18)	1.53 × 10^−04^	O	1.11 (0.97–1.27)	0.06	4.34 × 10^-05^
rs2431698	5	159884083	T	G	0.47	I	0.84 (0.81–0.89)	2.65 × 10^−08^	I	0.91 (0.80–1.05)	0.21	5.19 × 10^−08^
rs2431697	5	159879978	C	T	0.42	O	0.83 (0.79–0.88)	3.33 × 10^−09^	I	0.91 (0.79–1.04)	0.17	6.61 × 10^−09^
rs2431099	5	159886620	A	G	0.47	I	0.84 (0.80–0.89)	2.36 × 10^−08^	O	0.91 (0.80–1.05)	0.21	4.79 × 10^−08^
**XKR6**		Asthma, SLE										
rs4841466	8	10828909	T	C	0.46	I	1.17 (1.11–1.24)	3.77 × 10^−08^	I	1.40 (1.23–1.60)	3.46 × 10^−07^	2.73 × 10^−13^
rs11250098	8	10818607	A	G	0.48	I	1.16 (1.09–1.23)	3.87 × 10^−07^	I	1.39 (1.22–1.58)	7.77 × 10^−07^	6.89 × 10^−12^
rs11250099	8	10818657	A	G	0.47	I	1.17 (1.10–1.24)	5.99 × 10^−08^	I	1.40 (1.22–1.60)	4.97 × 10^−07^	5.97 × 10^−13^
rs4841465	8	10819854	C	T	0.48	O	1.17 (1.10–1.23)	9.42 × 10^−08^	I	1.42 (1.24–1.62)	1.72 × 10^−07^	4.85 × 10^−13^
rs3885690	8	10826869	C	T	0.37	I	0.85 (0.80–0.91)	5.47 × 10^−07^	O	0.78 (0.68–0.89)	5.45 × 10^−04^	1.32 × 10^−09^
**MAPT-CRHR1**		PBC										
rs7210219	17	44018519	C	T	0.19	I	0.78 (0.72–0.84)	2.40 × 10^−10^	–	–	–	–
rs2696609	17	44293205	G	A	0.20	I	0.78 (0.72–0.84)	2.77 × 10^−10^	–	–	–	–
rs7215239	17	43767773	C	T	0.21	O	0.80 (0.75–0.86)	8.74 × 10^−09^	–	–	–	–
**RPTOR-CHMP6-BAIAP6**												
rs8071514	17	78964083	C	A	0.44	I	0.84 (0.79–0.89)	1.64 × 10^−08^	–	–	–	–
rs6565516	17	78965146	A	G	0.41	I	0.85 (0.80–0.90)	1.26 × 10^−07^	–	–	–	–
rs4969331	17	78966915	C	T	0.41	O	0.85 (0.80–0.90)	2.13 × 10^−07^	–	–	–	–
**TYK2**		ATD, IIM, MS, PBC, RA, SLE, SSc, T1DM										
rs11085725	19	10462513	T	C	0.28	I	0.78 (0.73–0.83)	7.17 × 10^−13^	I	0.96 (0.82–1.11)	0.59	8.56 × 10^−11^
rs74908652	19	10828909	C	T	0.16	I	0.92 (0.85–0.99)	4.54 × 10^−02^	O	0.79 (0.65–0.95)	1.67 × 10^−02^	3.43 × 10^−03^
rs753859	19	10531224	C	G	0.48	I	0.87 (0.82–0.92)	2.56 × 10^−06^	I	0.92 (0.81–1.05)	0.22	2.88 × 10^−06^
rs34953890	19	10526854	A	C	0.20	I	0.83 (0.77–0.89)	1.94 × 10^−06^	I	0.98 (0.83–1.16)	0.84	2.39 × 10^−05^
rs2304256	19	10475652	A	C	0.28	O	0.79 (0.74–0.84)	9.14 × 10^−12^	I	0.95 (0.82–1.11)	0.55	5.38 × 10^−10^
**SYNGR1**		PBC, RA, SLE										
rs2069235	22	39747780	A	G	0.31	I	1.21 (1.14–1.28)	5.06 × 10^−10^	–	–	–	–
rs909685	22	39747671	A	T	0.31	I	1.21 (1.14–1.28)	5.90 × 10^−10^	–	–	–	–
rs3747177	22	39747780	A	C	0.24	I	1.21 (1.14–1.30)	3.93 × 10^−09^	–	–	–	–
rs5757611	22	39708357	T	C	0.22	O	1.19 (1.11–1.27)	1.70 × 10^−07^	–	–	–	–
Novel significant loci by meta-analysis (*P* < 5 × 10^−8^):												
**CD247**		Asthma, ATD, RA, SLE, SSc										
rs7523907	1	167427247	C	T	0.42	I	0.85 (0.80–0.90)	2.35 × 10^−07^	I	0.83 (0.73–0.96)	1.14 × 10^−02^	9.33 × 10^−09^
rs2949661	1	167424924	T	C	0.37	I	0.86 (0.81–0.91)	2.20 × 10^−06^	I	0.84 (0.73–0.96)	1.41 × 10^−02^	9.99 × 10^−08^
rs1723018	1	167433420	C	T	0.38	I	0.86 (0.81–0.91)	8.08 × 10^−07^	I	0.83 (0.73–0.92)	1.20 × 10^−02^	3.22 × 10^−08^
rs2056626	1	167420425	G	T	0.39	I	0.86 (0.81–0.91)	1.65 × 10^−06^	O	0.83 (0.73–0.96)	1.10 × 10^−02^	5.94 × 10^−08^
rs864537	1	167411384	G	A	0.39	O	0.92 (0.87–0.97)	7.80 × 10^−03^	O	0.82 (0.71–0.94)	6.49 × 10^−03^	2.51 × 10^−04^
**PRDM1-ATG5**		Asthma, RA, SLE SSc										
rs526531	6	106570056	A	G	0.32	I	1.13 (1.07–1.21)	2.1 × 10^−05^	O	1.28 (1.11–1.47)	3.84 × 10^−04^	4.86 × 10^−08^
rs533733	6	106564236	A	G	0.33	I	1.14 (1.07–1.21)	1.79 × 10^−05^	I	1.21 (1.05–1.39)	5.27 × 10^−03^	3.25 × 10^−07^
rs7768653	6	106574794	T	C	0.41	I	1.13 (1.06–1.19)	3.52 × 10^−05^	I	1.14 (0.99–1.30)	5.40 × 10^−02^	5.49 × 10^−06^
rs4946728	6	106590363	A	C	0.25	O	1.09 (1.02–1.16)	5.13 × 10^−03^	I	1.15 (0.99–1.33)	5.91 × 10^−02^	7.62 × 10^−04^
**TNFAIP3**		RA, SLE, SSc										
rs61117627	6	138243700	A	G	0.03	I	1.45 (1.26–1.67)	2.52 × 10^−07^	O	1.42 (1.02–1.98)	3.48 × 10^−02^	3.32 × 10^−08^
rs58905141	6	138132123	G	A	0.03	I	1.44 (1.24–1.67)	6.85 × 10^−07^	I	1.35 (0.96–1.92)	7.81 × 10^−02^	2.16 × 10^−07^
rs5029937	6	138195151	T	G	0.03	I	1.43 (1.25–1.65)	3.33 × 10^−07^	I	1.26 (0.91–1.75)	1.59 × 10^−01^	2.93 × 10^−07^
rs5029924	6	138187498	T	C	0.03	I	1.42 (1.23–1.63)	7.75 × 10^−07^	I	1.28 (0.92–1.77)	1.29 × 10^−01^	4.62 × 10^−07^
rs2230926	6	138196066	G	T	0.03	O	1.43 (1.25–1.65)	3.33 × 10^−07^	I	1.26 (0.91–1.75)	1.59 × 10^−01^	2.93 × 10^−07^
rs7749323	6	138230389	A	G	0.03	I	1.43 (1.23–1.65)	1.14 × 10^−06^	O	1.30 (0.92–1.84)	1.25 × 10^−01^	6.18 × 10^−07^

*ATD* autoimmune thyroid disease, *IIM* idiopathic inflammatory myopathies, *MS* multiple sclerosis, *PBC* primary biliary cirrhosis, *RA* rheumatoid arthritis, *SLE* systemic lupus erythematosus, *SSc* systemic sclerosis, *T1DM* type 1 diabetes mellitus.

^a^Disease associations indicated based on NHGRI-EBI GWAS Catalog.

^b^Minor allele (min); ^c^major allele (max); ^d^minor allele frequency (MAF); ^e^observed (O) or imputed (I) SNP in GWAS or immunochip analyses.

Since some of the identified regions overlapped with loci covered by the ImmunoChip 1.0, a meta-analysis was performed using ImmunoChip 1.0 data from an additional 619 Sjögren’s cases of European ancestry and 6171 population controls independent from the GWAS, focusing on regions with common genotyped SNPs (Supplementary Fig. 1H, I; Supplementary Data 2)^16^. The meta-analysis replicated four regions from the GWAS and identified three additional novel GWS associations (Fig. 2a, i–k; Table 1). It also increased the significance of the previously associated *IL12A*, *BLK*, and *CXCR5* loci from suggestive to GWS (Fig. 2a; Supplementary Data 1). *MAPT-CRHR1, RPTOR-CHMP6-BAIAP6*, and *SYNGR1* regions were not available for testing in the ImmunoChip 1.0 data. Collectively, this GWAS and meta-analysis increased the number of established Sjögren-associated loci surpassing GWS from 12 to 22.

### Polygenic risk score of Sjögren’s of European ancestry

**Fig. 3:**
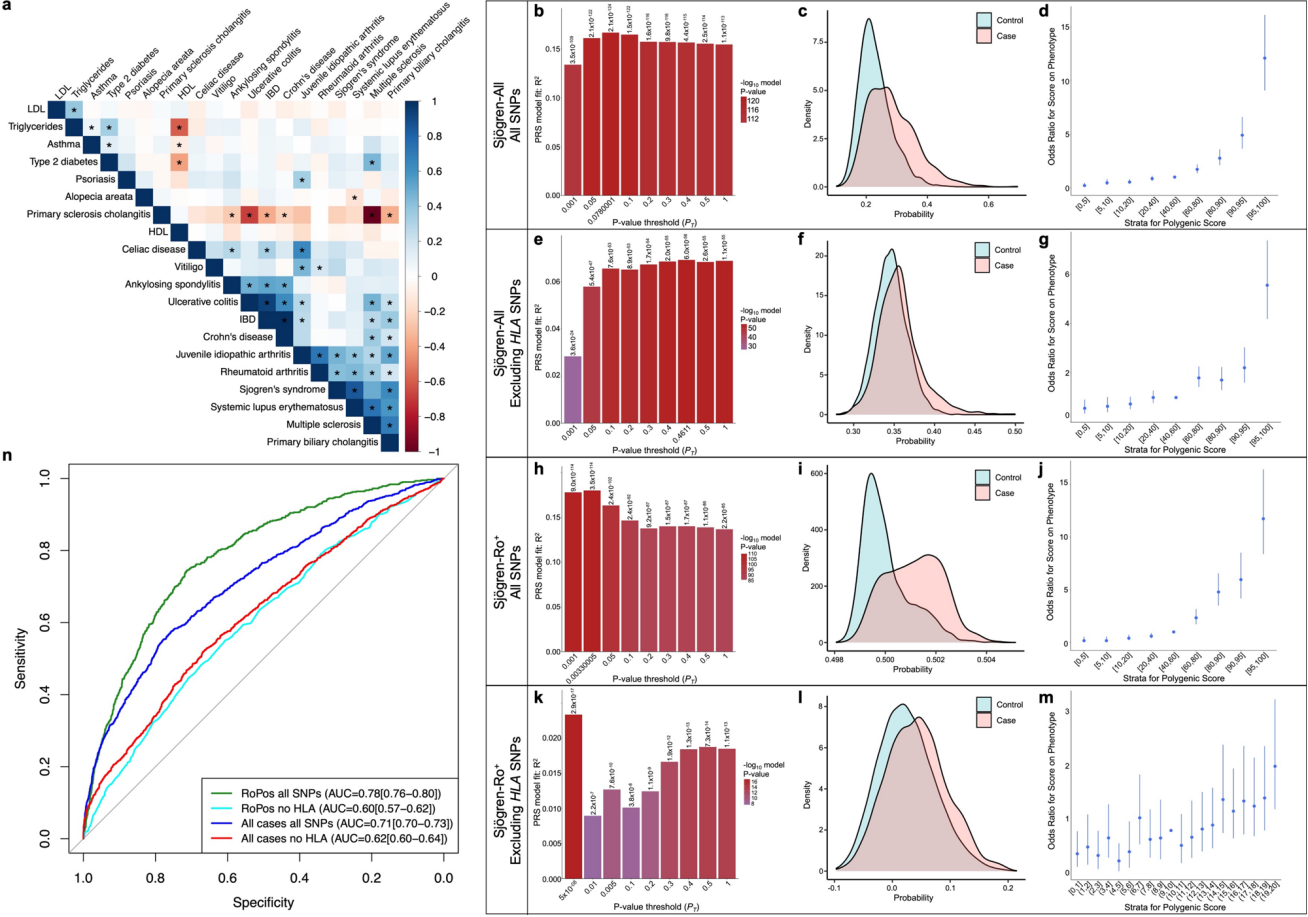
Polygenic risk score analysis of the Sjögren-SNPs in all genotype Sjögren’s cases and Ro^+^ Sjögren’s cases with or without the HLA region. **a** Heatmap of LDSC-estimated genetic correlations between Sjögren’s and 19 other immune-mediated diseases and other common traits using European GWAS summary data from the 1000 Genomes Project. Box color indicates magnitude of correlation; * indicates significant *P*-value after Bonferroni correction. **b**–**m** Polygenic risk scores (PRS) were calculated for all genotyped individuals from the Sjögren-All (**b**–**g**) or Sjögren-Ro^+^ (**h**–**m**) subsets, divided 2/3 into training and 1/3 into testing datasets, using LD-pruned genotyped SNPs (*r*^*2*^ > 0.2) including (**b**–**d**, **h**–**j**) or excluding (**e**–**g**, **k**–**m**) SNPs from the *HLA* region (6p21.3–22.3.). (**b**, **e**, **h**, **k**) Bar plot of multiple *P*-value thresholds (*P*_T_) for PRS prediction of Sjögren’s. **c**, **f**, **i**, **l** Histogram of the PRS distribution in Sjögren’s cases (orange) and controls (teal). **d**, **g**, **j**, **m** Strata plot of the odds ratio (OR) when comparing PRS from different quantile ranges. Bars indicate the 95% confidence intervals of the OR from each quantile range. **n** Area under the receiver operating characteristic curve (AUROC) values of PRS using LD-pruned genotyped Sjögren-SNPs including SNPs from 6p21.3–22.3 in all genotyped Sjögren’s cases (dark blue) or Ro^+^ Sjögren’s cases (green) relative to population controls, and LD-pruned genotyped Sjögren-SNPs excluding SNPs from 6p21.3–22.3 in all genotyped Sjögren’s cases (red) or Ro^+^ Sjögren’s cases (light blue) relative to population controls.

A cross-trait linkage disequilibrium score regression (LDSC) analysis of the GWAS summary statistics revealed that the risk alleles of Sjögren-SNPs strongly correlated with immune-mediated diseases, including SLE and RA (Fig. 3a). Polygenic risk scores analyses were performed to further reveal how this GWAS improves understanding of the genetic burden of Sjögren’s. All genotyped Sjögren’s individuals (Sjögren-All) and population control individuals were randomly divided into a training dataset (2/3 individuals; 2166 cases, 11,638 controls) or testing dataset (1/3 individuals; 1076 cases, 5826 controls) (Supplementary Fig. 2A, B). Sjögren-All individuals with reported Ro autoantibody status were further subset into Ro autoantibody positive (Sjögren-Ro^+^) or negative (Sjögren-Ro^−^) datasets, then the Sjögren-Ro^+^ subset was similarly divided into training (1100 cases, 11,544 controls) and testing (618 cases, 5896 controls) datasets for PRS calculations. Unfortunately, the Sjögren-Ro^−^ subset was too small for accurate PRS calculations. Sjögren-All and Sjögren-Ro^+^ training sets most accurately predicted case-control status at a *P* value threshold *P*_T_ = 0.078 or *P*_T_ = 0.003 with a PRS model fit Nagelkerke’s pseudo *R*^2^ = 0.167 (*P* = 2.076 × 10^−124^) and *R*^2^ = 0.179 (*P* = 3.48 × 10^−114^), respectively (Fig. 3b, c, h, i; Supplementary Fig. 2C–E; G–I).

The *HLA* region was the strongest GWS-Sjögren’s association (Fig. 2a) and is associated with Ro-52 and/or Ro-60 autoantibody positivity^21,31^. Removal of SNPs positioned in 6p21.3–22.3 from the PRS calculation yielded a best-predicted case-control status at a *P*_T_ = 0.461 and *P*_T_ = 5 × 10^−08^ and a model fit *R*^2^ = 0.069 (*P* = 6 × 10^−56^) and *R*^2^ = 0.023 (*P* = 2.93 × 10^−17^) for Sjögren-All and Sjögren-Ro^+^, respectively (Fig. 3e, f, k, l; Supplementary Fig. 2F, J). Area under the receiver operating characteristic (AUROC) curves were used to evaluate the accuracy of the two PRS models to distinguish case-control status. Removal of the *HLA* region reduced the AUROC from AUROC = 0.71 (95% CI = 0.70–0.73) to AUROC = 0.62 (95% CI = 0.60–0.64) in the Sjögren-All, and AUROC = 0.78 (95% CI = 0.706–0.80) to AUROC = 0.60 (95% CI = 0.57–0.62) in the Sjögren-Ro^+^ (Fig. 3n). Removal of the HLA also reduced the odds ratio (OR) in the Sjögren-All from OR = 12.08 (95% CI = 9.07–16.11) to OR = 5.54 (95% CI = 4.17–7.35) (Fig. 3d, f), and in the Sjögren-Ro^+^ from OR = 11.73 (95% CI = 8.38–16.41) to OR = 2.16 (95% CI = 1.38–3.38) when compared to the 40th–60th percentile reference interval (Fig. 3j, m).

### Sjögren-SNPs enriched in epigenetic and expression data

**Fig. 4:**
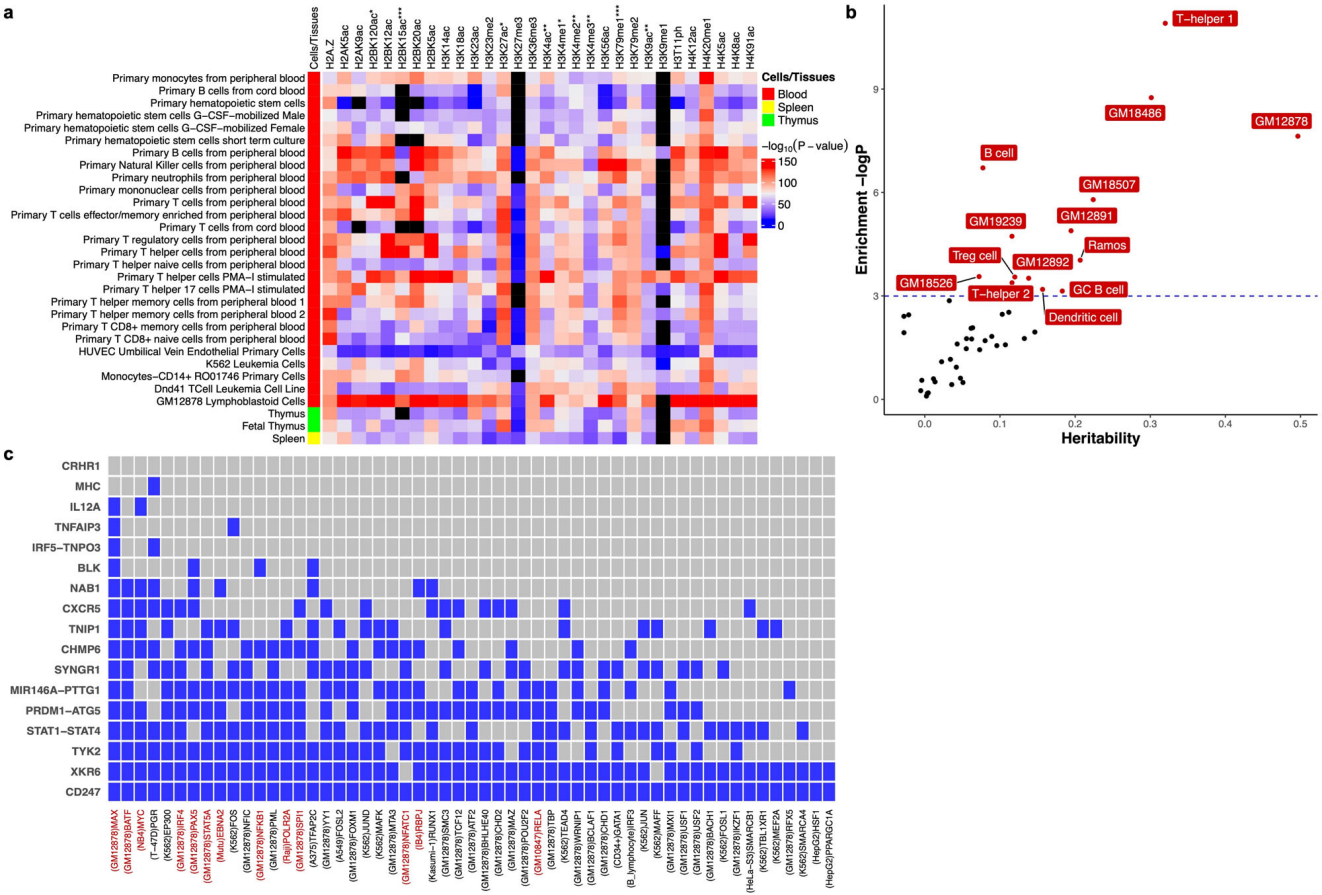
Epigenetic and transcription factor enrichment analysis of Sjögren’s risk loci in specific cell types and tissues. **a** Enrichment analysis of 30 histone marks in 127 different cell types and tissues from the Roadmap Epigenome Consortium Project was performed using GREGOR software. Heatmap displays the enrichment *P*-value for each histone mark plotted relative to specific immune cell types from the blood (red), spleen (yellow), and thymus (green). Black boxes indicate missing data. Complete analysis of all 127 cell types and tissues from the Roadmap Epigenome Consortium Project are reported in Supplemental Fig. 8 and Supplemental Data 3. **b** Enrichment of heritability in cell type-specific enhancers reported in the EnhancerAtlas2.0 database. Enrichment was calculated as partitioned heritability divided by the proportion of GWS Sjögren-SNPs that intersect with enhancer regions in each cell type. Cell types exhibiting significant GWS Sjögren-SNPs in enhancers are indicated in red. Blue dashed line is the threshold of significance after correction for multiple testing of *P* ≲ 0.05. **c** Enrichment of cell type-specific transcription factor binding sites. Transcription factors are indicated by a blue box if at least one Bonferroni-corrected (*P* ≲ 0.05) intersection between an indicated transcription factor and Sjögren-associated risk locus was detected. Red font indicates transcription factors that are also associated with the EBNA2 super enhancer. The cell type with the most significant interaction is listed in parentheses for each transcription factor.

GWASs disproportionately identify SNPs positioned in non-coding regions of the genome that most likely function by modulating the activity of regulatory elements that modify gene expression in specific cellular contexts^32^. Cell type- and tissue-specific epigenetic data from the Roadmap Epigenomics Consortium project were used to show that GWS Sjögren-SNPs were enriched in epigenetic marks of peripheral blood immune cell types^33,34^. Specifically, Sjögren-SNPs were most significantly enriched in (1) the histone H2B lysine 20 acetylation (H2BK20ac) peaks of GM12878 lymphoblastoid cell line (*P* = 4.02 × 10^−232^), primary B cells (*P* = 2.69 × 10^−135^), and primary natural killer cells (*P* = 1.13 × 10^−131^); (2) the histone H2A lysine 5 acetylation (H2AK5ac) peaks of GM12878 lymphoblastoid cell line (*P* = 1.03 × 10^−161^); (3) the histone H2B lysine 12 acetylation (H2BK12ac) peaks of primary T regulatory cells (*P* = 1.25 × 10^−137^) and primary neutrophils (*P* = 9.79 × 10^−107^); (4) the histone H4 lysine 20 mono-methylation (H4K20me1) peaks of primary monocytes (*P* = 1.45 × 10^−119^) (Fig. 4a; Supplementary Data 4).

A partitioned heritability analysis was performed using aggregated immune cell enhancer data obtained from EnhancerAtlas 2.0 [http://www.enhanceratlas.org/] to evaluate the enrichment of GWS Sjögren-SNPs in the enhancers of different immune cell subsets^35^. Significant enrichment of GWS Sjögren-SNPs was observed in the enhancers of T helper, T regulatory, dendritic, and B cells, with T helper 1 cells showing the most significant enrichment (*P* = 1.83 × 10^−5^), followed by GM18486 and GM12878 (*P* = 1.58 × 10^−4^ and *P* = 4.48 × 10^−4^, respectively) (Fig. 4b). Last, Regulatory Element Locus Intersection (RELI) analysis [https://github.com/WeirauchLab/RELI] was used to identify positional overlap between Sjögren’s risk loci and transcription factor binding sites, as well as the potential role for Epstein–Barr virus (EBV) in Sjögren’s, as had been reported in other related diseases^36,37^. The transcription factors, MYC-associated factor X (MAX), basic leucine zipper ATF-like transcription factor (BATF), and MYC proto-oncogene, BHLH transcription factor (MYC), exhibited the highest number of intersections with Sjögren’s risk loci (Fig. 4c). Further, seven of the ten transcription factors yielding the highest number of intersection counts with Sjögren’s risk loci were previously reported to be associated with EBV^38^. Moreover, the EBV-encoded protein, Epstein–Barr Virus nuclear protein 2 (EBNA2), occupied 8 of the 17 GWS Sjögren’s risk loci.

### Refinement of the novel Sjögren’s genetic associations

**Fig. 5:**
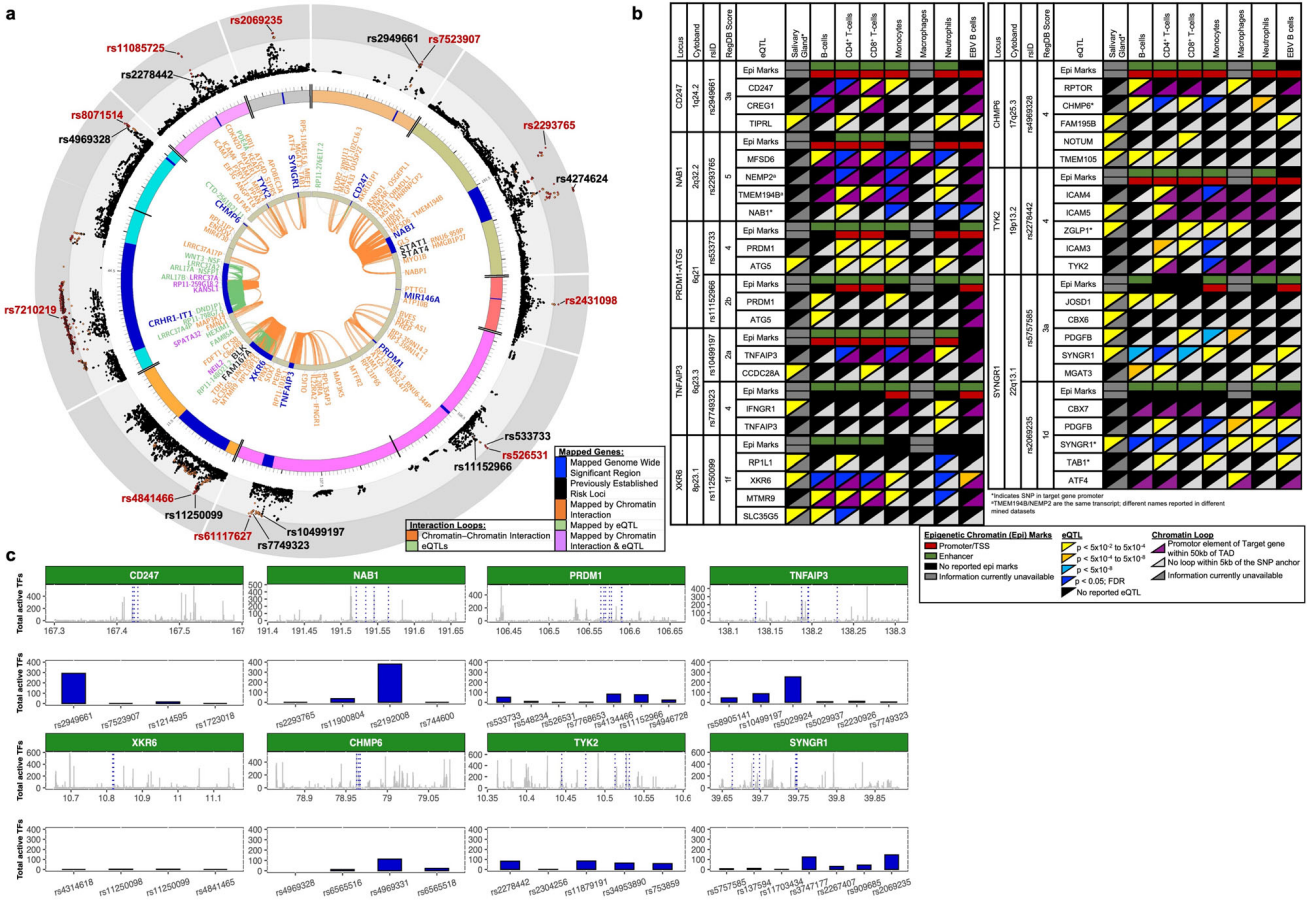
Chromatin Interactions and eQTLs of the ten novel Sjögren-associated genetic risk loci. **a** Circos plot shows the zoom regional Manhattan plots for each genetic risk locus (outer most layer); SNPs with *P*-value <0.05 (black); *r*^*2*^ > 0.08 (red); *r*^*2*^ > 0.06 (orange). Index SNP rsIDs are indicated in red. Black rsIDs are prioritized SNPs from the 95% credible set that are also eQTLs that exhibit chromatin-chromatin interactions and are shown in (**b**). Outer circle displays the chromosome coordinate with the genomic risk loci highlighted in blue. Genes that are eQTLs (green) or exhibit chromatin interaction by Hi-C in Epstein–Barr virus (EBV)-transformed B lymphocytes (orange) are reported on the inner circles as text or interaction links. Each index gene is colored blue. Genes that are eQTLs and engage in chromatin interactions are reported in purple. **b** Cell type-specific functional annotations (horizontal rectangles), select eQTLs (top triangles), and chromatin-chromatin interactions (bottom triangle) are shown for the indicated prioritized SNPs from each 95% credible set. *MIR146A* was omitted because mined eQTL databases did not test *MIR146A*. Complex linkage disequilibrium of the *CRHR1* association impaired refinement and fine-mapping of the region. **c** IMPACT annotation of the most likely functional Sjögren-SNPs to quantify SNP position in 700 cell-type-specific active transcription factor binding sites. Top panel depicts SNP position (blue lines) relative to genomic coordinates (Mb) of each indicated locus. Bottom panel shows the total number of active transcription factor binding sites detected at each indicated SNP.

Publicly available Hi-C data from the EBV B cell line, GM12878, and eQTL data from the DICE database in FUMA [http://fuma.ctglab.nl/]^39,40^ were used to map TADs and eQTLs reported for genes positioned in each Sjögren’s risk locus. A majority of the Sjögren’s risk loci exhibited strong linkage disequilibrium with many SNPs and enrichment of reported eQTLs and TADs in GM12878 (Fig. 5a), indicating that the risk alleles of these Sjögren-SNPs may modulate extended local regulatory networks to alter the expression of genes beyond the index gene, which is identified based on closest proximity to the index SNP. For example, one of the two associated regions on chromosome 17, *MAPT-CRHR1*, exhibited extended linkage disequilibrium with many SNPs spread broadly across several genes within the ~1.4 Mb region flanking the index gene (Supplementary Fig. 3; Supplementary Data 5, 6). Exploring TADs reported in various cell types revealed that many of the SNPs may have potential functional implications spanning up to 1.9 Mb from *MAPT-CRHR1* (Supplementary Fig. 3F). These data were further supported by the large number of strong cis-eQTLs and blood cell count traits reported for many of the SNPs in the Sjögren-associated *MAPT-CRHR1* locus (Supplementary Data 7).

Logistic regression and Bayesian analyses were used to refine the association signals from each of the 10 novel GWS regions, then posterior probability analyses were used to identify the 95% credible set of likely functional SNPs present in each region (Fig. 2b–k; Supplementary Figs. 3–12; Supplementary Data 5–34). SNPs from each credible set were independently interrogated for evidence of functionality using a variety of bioinformatics databases, including RegulomeDB [https://www.regulomedb.org/regulome-search/]^41,42^ and HaploReg [https://pubs.broadinstitute.org/mammals/haploreg/haploreg.php]^43^, and annotated accordingly. SNPs with the strong evidence of known and predicted regulatory functions, including transcription factor binding sites, reported promotor and enhancer activities, DNAse hypersensitivity, and eQTLs were selected for further functional dissection (Fig. 1b).

Coalescence of epigenetic marks with eQTLs mapped to TADs identified potential functional SNPs from each locus that most likely influence gene expression by one of four mechanisms: (1) intronic enhancers acting in *cis* and *trans* with a broader local regulatory network (*CD247*, *XKR6*, and *SYNGR1*) (Supplementary Figs. 4–6; Supplementary Data 8–16); (2) promoter and/or enhancer elements that act both in *cis* and *trans* within a broader local regulatory network (*NAB1* and *RPTOR-CHMP6-BAIAP6*) (Supplementary Figs. 7, 8; Supplementary Data 17–22); (3) intergenic enhancers interacting in *cis* (*PRDM1-ATG5* and *PTTG1-MIR146A*) (Supplementary Figs. 9, 10; Supplementary Data 23–28); and (4) intergenic enhancers acting in *cis* and *trans* (*TNFAIP3* and *TYK2*) (Supplementary Figs. 11, 12; Supplementary Data 29–34).

### Functional analysis of Sjögren-SNPs in intronic enhancers

Four variants at the first intron of CD247 molecule gene (*CD247*; encodes the T cell receptor ζ-chain) showed regulatory potential: rs7523907 (index SNP), rs2949661, rs1214595, and rs1723018 (Fig. 5; Supplementary Fig. 4; Supplementary Data 8–10). While rs7523907 and rs1723018 showed some coalescence between eQTLs, epigenetic marks, and TAD formation, rs2949661 and rs1214595 are eQTLs with corresponding TAD interactions with the promoter of *CD247* in CD4^+^ and CD8^+^ T cells (Fig. 5b; Supplementary Fig. 4G-J; Supplementary Data 10). Further, rs2949661 may alter the expression of cellular repressor of E1A stimulated genes 1 (*CREG1*), as both eQTLs and TAD coalesced in B and CD8^+^ T cells. Active transcription factor binding sites were also enriched at rs2949661 compared with other 95% credible SNPs in *CD247* (Fig. 5c). While TAD information is currently unavailable for salivary glands, eQTL data from salivary glands suggest that rs2949661 might modulate the expression of TOR signaling pathway regulator (*TIPRL*), and rs1723018 may influence pogo transposable element derived with KRAB domain (*POGK*) (Fig. 5b; Supplementary Fig. 4H, J; Supplementary Data 10). Finally, EcholocatoR^44^, an R package for automated statistical and functional fine-mapping [https://github.com/RajLabMSSM/echolocatoR], was used to further evaluate the expanded regulatory network of the *CD247* region and identify additional SNPs with potential functional significance. Independent EcholocatoR analyses, specifying each of the SNPs identified above as an index SNP, supported the identification of rs7523907 as a likely functional SNP and further identified rs2462552 and rs10800313 as additional SNPs of interest (Supplementary Fig. 4K).

The association region peaking at rs4841466 positioned in the intron of XK-related 6 (*XKR6*) is an independent association signal from the previously reported *FAM167A-BLK* region (Fig. 2d; Supplementary Fig. 5; Supplementary Data 11, 12)^45,46^; as observed in SLE^47,48^. Bioinformatic analyses revealed that the index variant, rs4841466, likely has no biological function (Supplementary Data 13); however, four additional potential functional variants positioned in a predicted intronic regulatory element of *XKR6* were identified in the 95% credible set: rs11250099, rs4841465, rs11250098, and rs4314618 (Supplementary Fig. 5G–J; Supplementary Data 11, 12). Coalescence between epigenetic marks, eQTLs, and TADs indicated that rs11250098 and rs11250099 are most likely to impact enhancer activity that may target *XKR6* and myotubularin-related protein 9 (*MTMR9*) (Fig. 5b; Supplementary Fig. 5G, I; Supplementary Data 13). Moreover, eQTL data in minor salivary glands implicated *XKR6*, RP1 like 1 (*RP1L1*), and solute carrier family 35 member G5 (*SLC35G5*). Interestingly, IMPACT analyses showed limited transcription factor binding activity (Fig. 5c), suggesting that specific cellular contexts may be required. EcholocatoR analyses using rs11250099, rs11250098, rs4841465, or rs4314618 as the index SNP all identified rs60724652 as an additional SNP of interest (Supplementary Fig. 5K–N).

The index SNP, rs2069235, positioned in the intronic region of synaptogyrin 1 (*SYNGR1*), has epimarks indicative of a promoter element for an alternate isoform in immune cells (Fig. 5b, Supplementary Fig. 6G; Supplementary Data 16). While the conditional analysis indicated a single effect in the region, several SNPs were present in the 95% credible set and exhibited bioinformatic indicators of function, including rs2069235, rs909685, rs2267407, and rs3747177 (Fig. 2h; Supplementary Fig. 6; Supplementary Data 14–16). The IMPACT annotation showed transcription factor binding activity for rs2069235 and rs3747177 (Fig. 5c). rs2069235 not only modulated *SYNGR1* expression in several immune cell types and minor salivary gland, but also showed coalescence of both TADs and eQTLs for activating transcription factor 4 (*ATF4*) and chromobox 7 (*CBX7*) in macrophages and neutrophils (Fig. 5b; Supplementary Fig. 6G; Supplementary Data 16). Closer evaluation of the SNPs in the 95% credible set revealed a second set of SNPs with much lower posterior probabilities that were located ~84.5 kb upstream of the peak association signal in the proximal promoter of *PDGFB*: rs5757585, rs11703434, rs137594 (Fig. 5b; Supplementary Fig. 6K–M; Supplementary Data 14–16). Several eQTLs, including *SYNGR1* and platelet-derived growth factor subunit B (*PDGFB*) in several immune cell types and the minor salivary gland, have been reported for rs5757585. Independent EcholocatoR analyses using rs2069235, rs909685, rs2267407, rs3747177, rs5757585, rs11703434, or rs137594 as the index SNP all identified rs470049 as an additional SNP enriched for bioinformatic indicators of function (Supplementary Fig. 6N–T). EcholocatoR analyses using rs5757585, rs11703434, or rs137594 also identified rs5757599 and rs11089938 as potential functional SNPs (Supplementary Fig. 6R–T).

### Functional analysis of Sjögren-SNPs in promoters and enhancers

On chromosome 2, an association peaking at rs2293765 positioned in the promoter region of *NAB1* was found to be independent of the previously established Sjögren’s risk locus, *STAT1-STAT4*, ~350 kb downstream of the NGFI-A binding protein 1 (*NAB1*) gene (Fig. 2b; Supplementary Data 17, 18)^49^. SNP rs2293765 had the highest posterior probability of the 95% credible SNP set (Supplementary Fig. 7B). Epigenetic regulatory marks, eQTLs, and TAD data suggest that rs2293765 may alter promoter activity and *NAB1* expression, as well as alter enhancer activity to modify major facilitator superfamily domain containing 6 (*MFSD6*), nuclear envelope integral membrane protein 2 (*TMEM194B*/*NEMP2*), and *AC093388.3* expression in multiple cell types (Fig. 5b; Supplementary Fig. 7G; Supplementary Data 19). *TMEM194B/NEMP2* was also a *cis*-eQTL in the minor salivary gland for three correlated variants: rs11900804 (*r*^2^ = 0.96), rs2192008 (*r*^2^ = 0.94), and rs744600 (*r*^2^ = 0.72) (Supplementary Fig. 7H–J; Supplementary Data 20). Our annotation analysis showed that rs2192008 has high transcription factor binding activity (Fig. 5c). EcholocatoR analyses of the *NAB1* region using rs2192008 and rs744600 as index SNPs identified rs1468685 as an additional SNP of interest (Supplementary Fig. 7L, N). Analyses using rs2293765 or rs11900804 as index SNPs did not identify any additional SNPs (Supplementary Fig. 7K, M).

The index SNP, rs8071514, positioned in the promoter region of *RPTOR-CHMP6-BAIAP6*, had the highest posterior probability of the 95% credible SNP set, but bioinformatic analyses suggested that it was not likely functional (Supplementary Fig. 8B; Supplementary Data 20–22). Four additional SNPs in the 95% credible set exhibited bioinformatic indicators of function, including epigenetic marks of both promoter and enhancer activity, eQTLs, and TADs in several immune cell types: rs4969328, rs6565516, and rs4969331 in the promoter region of charged multivesicular body protein 6 (*CHMP6*) and rs6565518 in a predicted intronic enhancer of *CHMP6* (Fig. 5b; Supplementary Fig. 8G–J; Supplementary Data 22). The fact that rs4969328, rs6565516, and rs4969331 were all eQTLs for *CHMP6* in immune cell types, despite TAD deficiency in the region, indicated that the three SNPs likely modulate *CHMP6* expression by altering promoter activity. In addition, rs4969328 exhibited moderate transcription factor binding activity, while there was no notable transcription factor binding activity in the other two SNPs (Fig. 5c). All three SNPs were also minor salivary gland eQTLs for TMEM105 long non-coding RNA (*TMEM105*) and small integral membrane protein 11 (*SMIM11*, a.k.a. *FAM165B*) ~314 kb and ~816 kb downstream, respectively (Fig. 5b; Supplementary Fig. 8G–J; Supplementary Data 22). Further, TAD and eQTL data suggested that rs4969328 may also modulate the activity of an enhancer that, in turn, modulates the activity of the *RPTOR*) promoter through long-range interactions in several immune cell types. EcholocatoR analyses using rs4969328, rs6565516, rs4969331 or rs6565518 as the index SNP all identified rs4969322, rs34050444, and rs8071514 as additional SNPs of interest (Supplementary Fig. 8K–N).

### Functional analysis of Sjögren-SNPs in intergenic enhancers

In the region of *PRDM1*-*ATG5*, GWS Sjögren-SNP association was observed after meta-analysis, peaking at rs526531 positioned 10 kb downstream of PR/SET domain 1 (*PRDM1*; encodes B lymphocyte-induced maturation protein 1 (BLIMP1)) (Fig. 2j; Supplementary Data 23, 24). Seven variants in the 95% credible set clustered in an intergenic region enriched with enhancer regulatory marks (Supplementary Fig. 9; Supplementary Data 25). Of these, four of the SNPs were eQTLs for *PRDM1* and autophagy-related 5 (*ATG5*) in immune cell types (Fig. 5b; Supplementary Fig. 9; Supplementary Data 25). Interestingly, *ATG5* was also an eQTL in the minor salivary gland for rs526531 and rs533733 (Fig. 5b; Supplementary Fig. 9G, I; Supplementary Data 25). Further, IMPACT analyses revealed elevated transcription factor binding activity at rs533733 (Fig. 5C). While we did not observe colocalization with TADs and eQTLs in this region, lymphoblastoid cell lines do show potential for these variants to interact with the promoters of both *PRDM1* and *ATG5*. EcholocatoR analyses also identified rs1008944 as an additional SNP of interest (Supplementary Fig. 9N–T).

The *TNFAIP3* region of association peaked at rs61117627 (Fig. 2k; Supplementary Data 29, 30) near the 3’ end of TNF alpha-induced protein 3 (*TNFAIP3*) gene. Bioinformatic analyses revealed that rs61117627 is not likely functional but identified several other correlated variants within the 95% credible set that are likely functional (Supplementary Data 31). Two variants, rs10499197 and rs58915141, are positioned in a likely enhancer upstream of *TNFAIP3* that engages in several TADs, including the *TNFAIP3* promoter, and are eQTLs for *TNFAIP3* in multiple immune cell types (Fig. 5b; Supplementary Fig. 11H, I; Supplementary Data 31). In addition, rs10499197 and rs58915141 have elevated transcription factor binding activity (Fig. 5c). rs5029924 had the highest transcription factor binding activity among the 95% credible SNPs (Fig. 5c), but no coalescence of eQTLs and TADs was observed for this SNP (Supplementary Fig. 11J; Supplementary Data 31). We did discover that rs7749323, the tagging SNP of the previously characterized TT > A variant contributing to hypomorphic *TNFAIP3* expression with the SLE risk haplotype^46,50,51^, is also an eQTL for *IFNGR1* in the minor salivary gland (Fig. 5b; Supplementary Fig. 11G; Supplementary Data 31). TAD data is currently unavailable for the salivary gland, but a TAD between the TT > A enhancer region and upstream interferon-gamma receptor 1 (*IFNGR1*) promoter was found in EBV B cells. Interestingly, EcholocatoR analyses only identified additional variants of interest when rs10499197 or rs58905141 were designated as index SNPs, identifying rs675640, rs142373084, and rs113237273 (Supplementary Fig. 11M–S).

In chromosome 19, the Sjögren-SNP association peaked at rs11085725, an intronic variant of tyrosine kinase 2 (*TYK2*) (Fig. 2g; Supplementary Data 32, 33). Although rs11085725 and rs35251378 had the highest poster probabilities of those in the 95% credible set (Supplementary Fig. 12B; Supplementary Data 32, 33), bioinformatic analyses identified three other SNPs, including the previously characterized missense variant in *TYK2*, rs2304256, as more likely to be functional (Fig. 5b; Supplementary Fig. 12G–I; Supplementary Data 34)^52–54^. Correlated variants, rs2278442 in an intronic region of intracellular adhesion molecule 3 (*ICAM3*) and rs11879191 in an intronic region of cell division cycle 37, HSP90 cochaperone (*CDC37*), both exhibited epigenetic regulatory marks indicative of both promoter and enhancer activity, as well as colocalization of TADs and *cis*-eQTLs that broaden this region of association to mitochondrial ribosomal protein L4 (*MRPL4*), intracellular adhesion molecule 1 (*ICAM1*), intracellular adhesion molecule 4 (*ICAM4*), intracellular adhesion molecule 5 (*ICAM5*), and eukaryotic translation initiation factor 3 subunit G (*EIF3G*). rs2278442 and rs11879191 also both exhibited higher transcription factor binding activity relative to rs2304256 (Fig. 5c). Further, *ICAM5*, which functions in innate immune responses^55,56^, and *EIF3G*, which regulates the initiation of protein translation^57,58^, were eQTLs in the minor salivary gland (Fig. 5b; Supplementary Fig. 12G–I; Supplementary Data 34). EcholocatoR analyses of index SNPs, rs2304256, rs11879191, and rs2278442, identified rs280525 as an additional SNP of interest (Supplementary Fig. 12J–L). Further, analysis of index SNPs, rs2304256 and rs2278442, identified the additional SNP rs8101195, while analysis of index SNP rs11879191 identified the additional SNP, rs753859.

## Discussion

This study identified ten Sjögren’s genetic susceptibility loci in the largest GWAS to date of Sjögren’s of European ancestry, nearly doubling the total number of identified genetic risk loci from 12 to 22. Although this list lags far behind the >120 risk loci identified for SLE or RA, the Sjögren-SNPs yielded similar PRS calculations (Sjögren-All AUROC = 0.71; Sjögren-Ro^+^ AUROC = 0.78; SLE AUROC = 0.72; RA AUROC = 0.76), suggesting that this GWAS accounts for a substantial portion of Sjögren’s heritability^25,26,59,60^. To understand how these ten novel risk loci contribute to Sjögren’s pathogenesis and identify the GWS Sjögren-SNPs responsible, we also fine mapped the loci and performed a deep bioinformatic analyses of the Sjögren-SNPs from each credible set of SNPs. We leveraged publicly available promoter-capture Hi-C data and eQTL data to map Sjögren-SNPs in the context of their (a) local regulatory network, (b) position relative to cell type-specific chromatin-chromatin interactions, and (c) potential function in specific immune cells and disease target tissues. Our deep bioinformatic analyses revealed new potential functional implications for the novel risk loci and further expanded the list of implicated genes to >40 (Supplementary Fig. 13).

PRSs assess the cumulative trait-associated genetic risk of an individual and, though limited in trans-ancestral applications, have accurately predicted an individual’s genetic risk for specific phenotypes, disease severity, and/or early onset of autoimmune disease in tightly controlled case-control studies^61^. Limitations of PRS applicability are due, in part, to the limited genetic load accounted for by algorithms that calculate the PRS based on the prevalence and/or odds ratio of selected GWS SNPs. By leveraging the PRSice-2 algorithm, which calculates PRSs using all genotyped SNPs (after LD-pruning), we obtained PRS calculations with a similar genetic load as previously reported for SLE and RA studies, despite having 5-fold fewer regions reaching genome-wide association^25,26,59,60^. Interestingly, removal of SNPs from the *HLA* region significantly reduced the predictability of the Sjögren’s PRS calculations in both the Sjögren-All and Sjögren-Ro^+^ datasets, thus indicating that the SNPs carried on the *HLA* are strong genetic risk factors for Sjögren’s and the anti-Ro autoantibody positive sub-phenotype. Due to the limited number of Sjögren-Ro^−^ individuals available in this study, we could not assess the impact of HLA on the genetic risk of the anti-Ro autoantibody negative phenotype. However, the residual effect observed in the Sjögren-All PRS predictions after *HLA* removal suggests that the *HLA* association may have a lower impact on the genetic risk of the Sjögren-Ro^−^ subphenotype^62^. Future studies focusing on the genetic risk of Sjögren-Ro^−^ patients are needed to assess these subphenotypic genetic differences.

Infiltration and proliferation of lymphocytes, particularly CD4^+^ T and B cells within the salivary gland, are driving forces of inflammation, impaired salivary secretion, and destruction of salivary glands in Sjögren’s^3–5,63,64^. Consistently, our pathway analyses of the 10 novel risk loci implicate potential alterations to immune cell function (*CD247*, *NAB1, MIR146A*, *PRDM1*, *TNFAIP3*, *TYK2*), inflammatory signaling (*TNFAIP3*, *CRHR1*, *TYK2*), cell survival and proliferation (*CD247*, *MIR146A*, *PRDM1*, *TNFAIP3*, *TYK2*), and cell stress (*ATG5*, *CHMP6*) (Supplementary Fig. 13).

Mapping the local regulatory networks and identifying additional genes of interest based on coalescence of reported TADs and cell type-specific eQTLs provided additional insights into the potential functional implications of susceptibility regions where the index gene function is unknown. For example, the functional implications of the *XKR6* susceptibility locus, identified herein and implicated in other autoimmune diseases^65–67^, remains largely unknown. Our bioinformatic analyses revealed that rs11250099 is positioned in an intronic enhancer of *XKR6* that forms a TAD with the promoter of *MTMR9* and is an eQTL for *MTMR9* in CD4^+^ and CD8^+^ T cells and B cells (Fig. 5b; Supplementary Fig. 5G; Supplementary Data 13). *MTMR9* encodes myotubulin-related protein 9, a protein that interacts and modulates the enzymatic activity of the autophagic inhibitor, MTMR8, in HeLa cells and Drosophila^68,69^. Given that autophagy is implicated in the regulation of B and T cell proliferation, survival, and ability to distinguish self from non-self^70,71^, it is interesting to hypothesize that the risk allele of rs11250099 may influence Sjögren’s susceptibility, in part, by modulating MTMR8/MTMR9-mediated autophagy^27–29^. However, to address this hypothesis, detailed functional studies, such as testing for allele-specific TAD formation between the *XKR6* intronic enhancer and the *MTMR9* promoter and the role of *MTMR9* in autophagy, in Sjögren’s are needed.

In addition to dysregulated immunity, analyzing genetic susceptibility in the context of salivary gland function may provide additional insights into why the salivary gland becomes a target tissue in Sjögren’s. Transcriptomic studies of the salivary gland have identified several differentially expressed genes associated with Sjögren’s pathology^3,4,6,64^; many of the salivary gland eQTLs reported herein are from similar studies. Unfortunately, most of these studies lack the cellular granularity to discern differential expression in salivary acinar and ductal cells from invading immune cells. Further, very little is known about the cell-type-specific chromatin architectures in the salivary gland. By exploring the local regulatory networks from EBV B cells, which are known to exhibit more promiscuous chromatin looping^72,73^, in the context of reported salivary gland eQTLs, we have garnered several new insights into how Sjögren’s genetic susceptibility may influence initial salivary gland dysfunctions.

*TNFAIP3* is a common autoimmune disease risk locus with several functionally characterized SNPs that regulate proinflammatory nuclear factor kB (NFκB) signaling implicated in the chronic inflammation and immune cell dysfunctions of autoimmunity^74^. The coding polymorphism, rs2230926, which propagates NFκB signaling by hindering *TNFAIP3* expression, is statistically associated with primary Sjögren-associated lymphoma^75,76^. Additionally, the *TNFAIP3* locus has a complex genomic architecture carrying several enhancers and regulatory elements, including the previously characterized TT > A enhancer ^50,51^. The tag SNP for the TT > A enhancer, rs7749323, was a GWS Sjögren-SNP in this GWAS and is an eQTL for *TNFAIP3* in neutrophils (Figs. 2k, 5b; Supplementary Data 31). Interestingly, rs7749323 is also a salivary gland eQTL for *IFNGR1* and engages the promoter of *IFNGR1* through chromatin looping in EBV B cells (Fig. 5b; Supplementary Data 31). IFNγ signaling is a potent driver of inflammation and immune activation in the salivary gland, and upregulation of interferon signaling is strongly correlated with autoimmunity^63,77,78^. In Sjögren’s, focal inflammation and the accumulation of IFNγ and other inflammatory cytokines contribute to exocrine gland dysfunction independent of lymphoid infiltration^78^. Therefore, our bioinformatic analyses implicate rs7749323 and allele-specific expression of *IFNGR1* as one of likely several potential mechanisms involved in the previously observed interferon signature of the salivary gland. Given that the mechanisms driving dysregulation of the salivary gland are largely understudied, these observations are meant to provide insights that help formulate hypotheses to test predicted mechanisms.

Inflammation and cell stress are known activators of autophagy, a process that has been implicated in the production of self-antigens in autoimmune disease target tissue when dysregulated^79–81^. Like our *IFNGR1* finding, we observed that rs533733 in the *PRDM1-ATG5* locus is a salivary gland eQTL for the autophagy regulator, *ATG5*, and is positioned in a TAD with the *ATG5* promoter in EBV B cells (Fig. 5b; Supplementary Data 25), leading us to hypothesize that the risk allele of rs533733 may also contribute to Sjögren’s pathogenesis by modulating autophagy in the salivary gland.

Interpretations of our bioinformatic analyses must consider the following limitations: (1) leveraged eQTL databases do not always report directionality, which limits the ability to interpret whether an implicated Sjögren-SNP increases or decreases gene expression; (2) the promoter-capture Hi-C data used for TAD mapping were obtained from quiescent cell types. Cellular microenvironments, including the presence/absence of inflammatory cytokines, can modulate gene expression in several ways, including altered transcription factor binding, regulatory element activity, and TADs between regulatory elements and target gene promoters^28–30^. Therefore, genes in the expanded gene list that have reported eQTLs, but lack cell-specific chromatin interactions, should be further examined in the context of specific stimuli or environments.

In conclusion, we performed a GWAS of Sjögren’s of European ancestry, then leveraged fine mapping and bioinformatic databases to determine the functional potential of GWS Sjögren-SNPs. Evaluating the local regulatory networks of each region in the context of immune cell type-specific eQTLs revealed that most of the regions have broad regulatory networks collectively involving >40 genes up- and downstream of the index gene. Further, pathway analyses of genes within these expanded local regulatory networks have diverse functional potential and may influence the pathogenesis of disease by altering cellular functions that might not have been otherwise considered (Supplementary Fig. 13). While our study demonstrates how deep bioinformatic analyses can expand the utility of GWAS data, we recognize that future functional studies in specific immune cell and tissue types at single-cell resolution in the minor salivary gland and in different disease subphenotypes will be needed to understand whether the genes we have identified within the broad regulatory networks of the ten novel Sjögren’s risk loci influence disease pathology.

## Methods

### Subjects

We obtained genotype data from a total of 3,885 Sjögren’s cases and 23,725 population controls of European ancestry collected from the United States of America, Australia, Austria, France, Germany, Hungary, Italy, Norway, Spain, Sweden, Switzerland, and United Kingdom (Supplementary Data 2). Genotype data were organized into six datasets: Dataset 1 (DS1; Phase 1)^16^, DS2 (Scandinavian-1), DS3 (Non-Scandinavian), DS4 (SICCA; phs000672.v1.p1 [https://www.ncbi.nlm.nih.gov/projects/gap/cgi-bin/study.cgi?study_id=phs000672.v1.p1])^19^, DS5 (Scandinavian-2), and DS6 (PRECISESADS)^82^ (Fig. 1a; Supplementary Data 2). In addition, we performed meta-analysis using case-control ImmunoChip 1.0 data in DS7^16^ (Supplementary Data 2). All datasets were subjected to quality control measures described below. Genetic matching was performed for each case from DS2 to five population controls using identity-by-state, as implemented in PLINK (v1.09) [https://www.cog-genomics.org/plink/], to assess allele sharing, then remaining controls were used in DS3 and DS4.

All cases fulfilled the American-European Consensus Group (AECG) criteria for primary Sjögren’s disease according to clinical evaluations performed within their respective cohort^83,84^. Anti-Ro autoantibody positivity was determined according to the approved study protocol of each respective cohort and reported as positive or negative by each respective cohort for this study. Written informed consent was obtained in accordance with the Institutional Review Boards of each respective cohort. All study protocols and informed consent documents from outside institutions were reviewed and approved by the OMRF Institutional Review Board. Then, this study was conducted in accordance with the OMRF Institutional Review Board approval.

### Genotyping and QC

Ilumina Infinium Omni1 or Omni2.5 Genome-Wide Genotyping Array kits were used to genotype DS1^16^ and DS4^19^. Illumina OmniExpress kit was used to genotype DS2, DS3, and DS5. DS6 was genotyped using the Illumina Global Screening Array (GSA) kit following Infinium chemistry. DS7 was genotyped using the ImmunoChip 1.0 array^16^. Strict quality control procedures were applied to each dataset: (i) subjects within well-defined cluster scatter plots; (ii) SNP having MAF > 1%; (iii) SNPs and samples each with call rate >95%; (iv) controls with Hardy–Weinberg proportion test with *P* > 0.001; (v) cases and controls with differential missingness (*P* > 0.001) were selected for downstream analysis. PLINK (v1.9) was used to merge the quality controlled DS1, DS2, DS3, DS4, DS5, and DS6 into a single merged dataset, then quality control procedures were applied to the merged genotyped dataset^85^.

Individual genotyped data were excluded from the merged genotyped dataset and DS7 if it had <95% call rate and excessive heterozygosity (>5 s.d. from the mean). PLINK (v1.9) was used to determine relatedness within the remaining samples using identity-by-descent (IBD) estimates^85^. One individual from each pair was removed if the proportion of the alleles that shared IBD was >0.4. Base pair positions were assigned according to the GRCh37/hg19 version of the human genome reference sequence. After quality control, 3,232 cases and 17,481 population controls in the merged genotyped dataset, and 619 cases and 6171 controls in DS7 were available for subsequent analyses (Fig. 1a; Supplementary Data 2).

### Assessment of population stratification

Population substructure within the PI1 and DS7 datasets was determined using EIGENSTRAT [https://www.hsph.harvard.edu/alkes-price/software/] with 53,108 (merged genotyped dataset) or 16,596 (DS7) independent markers (*r*^*2*^ < 0.20 between variants), respectively^86^. Eigenvectors distinguish five continental ancestral populations from the 1000 Genomes Project samples: East Asian (CHB, JPT, CHS, CDx, KHV), Admixed American (MXL, PUR, CLM, PEL), European (CEU, TSI, FIN, GBR, IBS), African (YRI, LWK, GWD, MSL, ESN, ASW), and South Asian (GIH, PJL, BEB, STU, ITU)^87^. Principal component analysis (PCA) was used to identify and remove outliers defined as having standard deviations greater than 6 (s.d. > 6) from the mean. Case and control samples used in the merged genotyped dataset and DS7 were plotted by principal components (PC) 1 and PC2, consistent with a European ancestral population.

### Imputation

Whole-genome imputation was performed using the Haplotype Reference Consortium panel version 1.1 (*n* = 32,611; human haplotypes = 64,976; SNPs = 39,235,157) on the Michigan Imputation Server, a free service for large-scale population studies [https://imputationserver.sph.umich.edu/]^88^. Each dataset (DS1-DS7) was imputed individually, using only the variants that passed quality control. SHAPEIT [https://odelaneau.github.io/shapeit4/] was used for prephasing and Minimac3 [https://github.com/Santy-8128/Minimac3] for the imputation^89,90^. To be included in analyses, imputed variants had to meet or exceed the imputation quality score (INFO) > 0.5 and quality control criteria described above. Imputed variants from datasets DS1-DS6 were merged to create dataset PI1 (Fig. 1a) and subjected to quality control criteria described above. Whole-genome imputation increased the number of SNPs tested for single-marker trait association from 101,574 to 6,257,359.

### Statistical analysis

Logistical regression models were computed using PLINK to test for single marker SNP-Sjögren’s association in the post-imputation PI1 dataset^85^. The additive genetic model was calculated for the 22 autosomal chromosomes, while adjusting for the first four principal components. PC1-PC4 accounted for >80% of the variation in the dataset after quality control.

To analyze imputed SNPs on the X chromosome, two separate logistical regression analyses were performed, one for each sex, using the same quality-controlled samples from the analysis of the autosomal genome and adjusting for the first four principal components. A dosage compensation model was used to account for inactivation of chromosome X, thereby assigning the SNPs as 0 or 2 (i.e., males were treated as homozygotes for the present allele). Logistic results of both sexes were then meta-analyzed to determine the common effect of chromosome X variants for the disease using a weighted Z-score in METAL [http://csg.sph.umich.edu/abecasis/metal/]^91^.

Results from the logistic regression analyses of the novel GWS regions (*P* < 5 × 10^−8^), 74 suggestive GWS regions (*P* < 5 × 10^−5^), and previously established regions were meta-analyzed with DS7 data using a fixed-effect model in METAL by weighing the SNP effect by sample size^91^. Cochran’s Q test statistic and I^2^ index were both used to test for meta-analysis heterogeneity^92,93^. LD and probable haplotype blocks were determined using the solid spine of LD algorithms in the HAPLOVIEW software v4.2 [https://www.broadinstitute.org/haploview/haploview], and a threshold of *r*^*2*^ > 0.8^94^.

### Polygenic risk score calculation

Genotyped individuals were randomly separated into a training dataset (2/3rd of the individuals) and testing dataset (1/3rd of the individuals) to calculate individual polygenic risk scores (PRS). PRSs were calculated for both the Sjögren-All and Sjögren-Ro^+^ dataset (Fig. 1a). Anti-Ro autoantibody positivity was determined and reported by each respective cohort as described above. To correct for population stratification, PCA analyses were performed for each of the datasets separately, then the first three PCs were used as covariates in the PRS analysis. Data was pruned to remove highly correlated SNPs using independent pairwise analysis with a window size of 50 kb, step size or variant count of 5, and *r*^*2*^ > 0.2 in PLINK (v1.9)^85,94^, then PRSice-2 v2.3.3 [https://www.prsice.info/]^95^ was used to calculate PRSs. After pruning, the Sjögren-All training dataset included 2166 cases and 11,638 population controls and testing dataset included 1076 cases and 5826 population controls. The Sjögren-Ro^+^ testing dataset included 1100 cases and 11,544 population controls and training dataset included 618 cases and 5,894 population controls. In subsequent analyses, the *HLA* region (24–37 Mb) was dropped and PRSs were recalculated using PRSice-2. PRSs were generated at multiple *P* value thresholds (*P*_T_) ranging from *P* = 0.001 to *P* = 1. The best-fit thresholds were used to predict Sjögren’s status under logistic regression, while adjusting for the three PCs using the general linear model (glm) function in R 3.6.0. Distribution of PRSs among cases and controls were plotted using R. Area under the receiver operating characteristic curves (AUROC) were used to evaluate the accuracy of the two PRS models to distinguish the case from control status using the pROC package [http://web.expasy.org/pROC/] in R v4.0.4 [http://www.r-project.org/].

### Genetic correlation with autoimmune diseases

Cross-trait linkage disequilibrium score regression (LDSC) was performed on the GWAS summary statistics using LDSC software [https://github.com/bulik/ldsc]^96^ to estimate the degree of genetic correlation between Sjögren’s and 19 other immune-mediated diseases and other common traits. Publicly available summary statistics were obtained for 19 other diseases and traits [https://alkesgroup.broadinstitute.org/sumstats_formatted/?C=S;O=A]. The LD Score computed using European data from the 1000 Genomes Project was used as the reference panel [https://alkesgroup.broadinstitute.org/LDSCORE]^97^. Analysis was limited to the Sjögren-associated GWS SNPs imputed using HapMap3 as implemented in the LDSC software. Traits with a genetic correlation (rg) of *p* < 0.05 were considered as genetically correlated with Sjögren’s.

### Cells and tissue-specific epigenetic enrichment and Sjögren’s heritability

Partitioned LD Score Regression (LDSC)^96^ was performed to estimate enrichment of Sjögren’s risk loci in cell-type-specific enhancer peaks (Fig. 1B). Cell type- and tissue-specific annotations were downloaded from EnhancerAtlas2 database [http://www.enhanceratlas.org/]^35^. LD scores were calculated based on EnhancerAtlas2 custom annotation, linkage pattern derived from European samples from 1000 Genomes Project and the baseline model suggested by Finucane et al.^97^. Epigenomic enrichments of genetic variants were tested using GREGOR [http://csg.sph.umich.edu/GREGOR/]^98^. Sjögren’s variants with *P* < 5 × 10^−8^ were tested for the enrichment in 4035 genomic features. The saddle-point approximation was used to estimate the enrichment *P* value by comparing it to the distribution of permuted statistics^97^. The enrichment was considered significant if the enrichment *P* value was less than the Bonferroni-corrected threshold of 1.23 × 10^−5^ (nominal *P* = 0.05 of 4035 tested genomic features). Imputed narrow peak data from the Roadmap Epigenomics Project was used for this purpose^33,34^.

### Transcription factor enrichment in Sjögren’s risk loci

Regulatory Element Locus Intersection (RELI) algorithm^37^ was used to identify transcription factors intersecting with Sjögren’s risk loci (Fig. 1b). Sjögren’s variants with *P* < 5 × 10^−8^ were used as input. RELI picks the most significantly associated SNP in each risk loci as an anchor SNP for calculating the LD blocks using sequencing data from the 1000 Genomes Project [http://ftp.1000genomes.ebi.ac.uk/vol1/ftp/release/20130502/]. Variants with *r*^*2*^ > 0.8 with the top significant SNPs were prepared as input for RELI. RELI was run with the default parameters to calculate the z-score and *P*-value for each transcription factor, and Bonferroni corrected to obtain the final *P*-value.

### Fine-mapping

To identify the independent associations at each of the seven novel GWS loci (*P* < 5 × 10^−8^), logistic regression was performed while adjusting for the most significant variants (i.e. conditional analysis; *P* < 0.0001). LocusZoom [http://locuszoom.org/genform.php?type=yourdata] was used to plot logistic regression results for each independent Sjögren-associated region^99^. Bayesian analyses were used to further refine the association signal in each GWS region. Posterior probability (PP) estimates were calculated for each SNP in the GWS region as if the SNP was a true putative casual variant. Then, a credible set of SNPs (PP > 95%) was defined using the Trinculo software package [https://sourceforge.net/projects/trinculo/files/]^100,101^.

Fine-mapping was also performed separately using EcholocatoR R package [https://github.com/RajLabMSSM/echolocatoR]^44^ that uses SuSiE and PolyFun+SuSiE and summary statistics to analyse SNPs from a 1 Mb window centered on each designated index SNP (i.e., ±0.5 Mb flanking the SNP). Both approaches used the Bayesian model and provided posterior probability (PP) to each SNP on a scale from 0 to 1 and defined the credible sets based on PP ≥ 0.95. In both models, the maximum number of casual SNPs was set to 5. Enrichment of GWAS SNPs was performed using the following annotations: (1) cell and tissue-type specific regulatory chromatin states from Roadmap Epigenomics Consortium, (2) ENCODE transcription factor binding sites in different cell types, and (3) regulatory chromatin states from DNAse hypersensitivity (Roadmap Epigenomics Consortium).

### Functional annotation

SNPs in the 95% credible set were annotated for potential function using FUMA version v1.3.5e [http://fuma.ctglab.nl/]^102^. FUMA is an online platform for the functional mapping and annotation of genetic variants to define genomic risk loci and obtain functional information of the relevant variants in that locus. FUMA identifies SNPs that have a GWAS *P* value (*P* < 5 × 10^−8^) and are not in LD (*r*^*2*^ < 0.6). Then, it provides a list of all known SNPs, regardless of presence in the GWAS input, that are in LD (*r*^*2*^ ≥ 0.6) with the independent SNPs for further annotation. Index SNPs were defined as independent SNPs with an *r*^*2*^ < 0.1. Independent Sjögren-associated genomic risk loci were defined as regions that are both in LD with an index SNP and separated by >250 kb from another region of association. Hi-C data of GM12878 and eQTL from DICE database in FUMA was utilized for chromatin interaction mapping with a false discovery rate (FDR) cutoff of <1 × 10^−5^ to define the significant interactions. FUMA performs chromatin interaction mapping, overlapping independent significant SNPs and SNPs in LD with one end of significantly interacting regions in user-defined tissues/cell types. Predicted enhancer regions were selected in any of the 111 tissues/cell types from the Roadmap Epigenomics Project^34^ and the promotor region (250 bp up and 500 bp downstream of transcription start site) as predicted by Roadmap Epigenomics Project^34^. Circos software in FUMA was used to visualize associated regional plots, genomic risk loci, chromatin interactions, and eQTLs mappings.

Candidate functional SNPs from the 95% credible sets were further prioritized based on data mining from HaploReg [https://pubs.broadinstitute.org/mammals/haploreg/haploreg.php]^43^, RegulomeDB [https://www.regulomedb.org/regulome-search/]^41,42^, and Open Targets [https://www.opentargets.org]^103^ databases. Publicly available repositories, including eQTL Catalogue^104^, single-cell RNA sequencing eQTLs^105^, Database of Immune Cell eQTLs and Epigenomics (DICE)^39,40^, and minor salivary gland (GTEx v8)^106^ were mined, then FUMA was used to map all tested SNPs to genes up to 1 Mb apart that show a significant (*P* < 0.05) *cis*-eQTL association. We also used QTLbase [http://mulinlab.tmu.edu.cn/qtlbase] to retrieve significant (*P* < 0.05) *cis*-eQTL information on SNPs and mapped genes.

Pre-processed capture Hi-C data for the candidate functional variants from 14 immune cell types and the GM12878 cell line were obtained from the 3D Genome Browser [http://3dgenome.fsm.northwestern.edu/chic.php]^107^. Promotor and enhancer sites located 5 kb on either side of each SNP were queried and the start and end coordinates for each loop were obtained. Consensus loops for each SNP were defined, then the start and end coordinates of each consensus loop were visualized in the UCSC Genome Browser [http://genome.ucsc.edu/] to find nearby or overlapping genes.

### Risk loci and IMPACT annotation

To have a better understanding of the link between the Sjögren’s risk loci and transcription factor binding sites, the risk loci were annotated against ~700 cell-type-specific active transcription factor binding sites from the IMPACT model (Fig. 1b)^108^. First, the total number of active transcription factor binding sites was calculated for each genomic position. A genomic position with an IMPACT score >0.5 was reported as an active transcription factor binding site. The candidate SNPs in Sjögren’s risk loci against the total active binding sites were then annotated.

### Functional pathway analysis

Candidate functional genes that coalesced with eQTLs and TAD interactions were selected for canonical pathway analysis and disease and function analysis using the Ingenuity Pathway Analysis Program (version: 60467501, Ingenuity System).

### Reporting summary

Further information on research design is available in the Nature Research Reporting Summary linked to this article.

## Supplementary information

Supplementary Information

Description of Additional Supplementary Files

Supplementary Data 1-34

Reporting Summary

## Data Availability

The genome-wide association summary statistics generated in this study have been deposited in the Databases of Genotypes and Phenotypes (dbGaP) under accession number: phs002723.v1.p1. Individual-level genotype data for 1199 subjects included in this study are available via dbGAP controlled access under accession number: phs002723.v1.p1. Individual-level genotype data for 10,850 subjects are available via dbGAP controlled access under accession numbers: phs000672.v1.p1 (*n* = 735), phs000428.v2.p2 (*n* = 8519), phs000196.v3.p1 (*n* = 995), phs000187.v1.p1 (*n* = 602). Remaining pre-existing individual-level genotype data were generated by coauthors and provided for specific use in this study and cannot be publicly shared per data use agreements. Contact the corresponding author (chris-lessard@omrf.org) with inquiries about accessing this pre-existing genotyping data. All other data presented in this study was previously published and can be accessed by: Haplotype Reference Consortium panel version 1.1 (Michigan Imputation Server) [https://imputationserver.sph.umich.edu/], 1000 Genomes Project reference data [http://ftp.1000genomes.ebi.ac.uk/vol1/ftp/release/20130502/], EnhancerAtlas 2.0 database [http://www.enhanceratlas.org/], FUMA [http://fuma.ctglab.nl/], RegulomeDB [https://www.regulomedb.org/regulome-search/], Open Targets [https://www.opentargets.org], Haploreg [https://pubs.broadinstitute.org/mammals/haploreg/haploreg.php], GWAS Summary used in LDSC analysis [https://alkesgroup.broadinstitute.org/sumstats_formatted/?C=S;O=A], LD Score European data from the 1000 Genome Project [https://alkesgroup.broadinstitute.org/LDSCORE], 3D Genome Browser [http://3dgenome.fsm.northwestern.edu/chic.php], UCSC Genome Browser [http://genome.ucsc.edu/], QTLbase [http://mulinlab.tmu.edu.cn/qtlbase].
