# Author Correction: Genome-wide association study identifies Sjögren’s risk loci with functional implications in immune and glandular cells

**DOI:** 10.1038/s41467-022-34311-8

**Published:** 2022-10-31

**Authors:** Bhuwan Khatri, Kandice L. Tessneer, Astrid Rasmussen, Farhang Aghakhanian, Tove Ragna Reksten, Adam Adler, Ilias Alevizos, Juan-Manuel Anaya, Lara A. Aqrawi, Eva Baecklund, Johan G. Brun, Sara Magnusson Bucher, Maija-Leena Eloranta, Fiona Engelke, Helena Forsblad-d’Elia, Stuart B. Glenn, Daniel Hammenfors, Juliana Imgenberg-Kreuz, Janicke Liaaen Jensen, Svein Joar Auglænd Johnsen, Malin V. Jonsson, Marika Kvarnström, Jennifer A. Kelly, He Li, Thomas Mandl, Javier Martín, Gaétane Nocturne, Katrine Brække Norheim, Øyvind Palm, Kathrine Skarstein, Anna M. Stolarczyk, Kimberly E. Taylor, Maria Teruel, Elke Theander, Swamy Venuturupalli, Daniel J. Wallace, Kiely M. Grundahl, Kimberly S. Hefner, Lida Radfar, David M. Lewis, Donald U. Stone, C. Erick Kaufman, Michael T. Brennan, Joel M. Guthridge, Judith A. James, R. Hal Scofield, Patrick M. Gaffney, Lindsey A. Criswell, Roland Jonsson, Per Eriksson, Simon J. Bowman, Roald Omdal, Lars Rönnblom, Blake Warner, Maureen Rischmueller, Torsten Witte, A. Darise Farris, Xavier Mariette, Marta E. Alarcon-Riquelme, PRECISESADS Clinical Consortium, Caroline H. Shiboski, Sjögren’s International Collaborative Clinical Alliance (SICCA), Marie Wahren-Herlenius, Wan-Fai Ng, UK Primary Sjögren’s Syndrome Registry, Kathy L. Sivils, Indra Adrianto, Gunnel Nordmark, Christopher J. Lessard

**Affiliations:** 1 Genes and Human Disease Research Program, Oklahoma Medical Research Foundation, Oklahoma City, OK USA; 2 Arthritis and Clinical Immunology Research Program, Oklahoma Medical Research Foundation, Oklahoma City, OK USA; 3 Department of Clinical Science, University of Bergen, Bergen, Norway; 4 NGS Core Laboratory, Oklahoma Medical Research Foundation, Oklahoma City, OK USA; 5 Salivary Disorder Unit, National Institute of Dental and Craniofacial Research, Bethesda, MD USA; 6 Center for Autoimmune Diseases Research (CREA), Universidad del Rosario, Bogotá, Colombia; 7 Department of Oral Surgery and Oral Medicine, Faculty of Dentistry, University of Oslo, Oslo, Norway; 8 Department of Health Sciences, Kristiania University College, Oslo, Norway; 9 Department of Medical Sciences, Rheumatology and Science for Life Laboratory, Uppsala University, Uppsala, Sweden; 10 Department of Rheumatology, Faculty of Medicine and Health, Örebro University, Örebro, Sweden; 11 Department of Rheumatology and Immunology, Hannover Medical School, Hannover, Germany; 12 Department of Rheumatology and Inflammation Research, Sahlgrenska Academy at University of Gothenburg, Gothenburg, Sweden; 13 Department of Rheumatology, Haukeland University Hospital, Bergen, Norway; 14 Department of Internal Medicine, Clinical Immunology Unit, Stavanger University Hospital, Stavanger, Norway; 15 Section for Oral and Maxillofacial Radiology, Department of Clinical Dentistry, Medical Faculty, University of Bergen, Bergen, Norway; 16 Rheumatology Unity, Department of Medicine, Karolinska University Hospital, Karolinska Institutet, Stockholm, Sweden; 17 Academic Specialist Center, Center for Rheumatology and Studieenheten, Stockholm Health Services, Region Stockholm, Sweden; 18 Translational Sciences, The Janssen Pharmaceutical Companies of Johnson & Johnson, Spring House, PA USA; 19 Rheumatology, Department of Clinical Sciences Malmö, Lund University, Malmö, Sweden; 20 Instituto de Biomedicina y Parasitología López-Neyra, Consejo Superior de Investigaciones Científicas (CSIC), Granada, Spain; 21 Université Paris-Saclay, Assistance Publique–Hôpitaux de Paris (AP-HP), Hôpital Bicêtre, Institut National de la Santé et de la Recherche Médicale (INSERM) UMR1184, Le Kremlin Bicêtre, France; 22 Department of Rheumatology, Stavanger University Hospital, Stavanger, Norway; 23 Department of Rheumatology, University of Oslo, Oslo, Norway; 24 Department of Pathology, Haukeland University Hospital, Bergen, Norway; 25 Department of Medicine, Russell/Engleman Rheumatology Research Center, University of California San Francisco, San Francisco, California USA; 26 Genyo, Center for Genomics and Oncological Research, Pfizer/University of Granada/Andalusian Regional Government, Granada, Spain; 27 Department of Rheumatology, Skåne University Hospital, Malmö, Sweden; 28 Medical Affairs, Jannsen-Cilag EMEA (Europe/Middle East/Africa), Beerse, Belgium; 29 Division of Rheumatology, Cedars-Sinai Medical Center, Los Angeles, CA USA; 30 David Geffen School of Medicine, University of California Los Angeles, Los Angeles, CA USA; 31 Hefner Eye Care and Optical Center, Oklahoma City, OK USA; 32 Oral Diagnosis and Radiology Department, University of Oklahoma College of Dentistry, Oklahoma City, OK USA; 33 Department of Oral and Maxillofacial Pathology, University of Oklahoma College of Dentistry, Oklahoma City, OK USA; 34 Department of Ophthalmology, Dean McGee Eye Institute, University of Oklahoma Health Sciences Center, Oklahoma City, OK USA; 35 Department of Medicine, University of Oklahoma Health Sciences Center, Oklahoma City, OK USA; 36 Department of Oral Medicine/Oral & Maxillofacial Surgery, Atrium Health Carolinas Medical Center, Charlotte, NC USA; 37 Department of Otolaryngology/Head and Neck Surgery, Wake Forest University School of Medicine, Winston-Salem, NC USA; 38 Department of Pathology, University of Oklahoma Health Sciences Center, Oklahoma City, OK USA; 39 US Department of Veterans Affairs Medical Center, Oklahoma City, OK USA; 40 Institute of Human Genetics (IHG), University of California San Francisco, San Francisco, CA USA; 41 Genomics of Autoimmune Rheumatic Disease Section, National Human Genome Research Institute, NIH, Bethesda, MD USA; 42 Department of Biomedical and Clinical Sciences, Division of Inflammation and Infection, Linköping University, Linköping, Sweden; 43 Rheumatology Department, University Hospital Birmingham NHS Foundation Trust, Birmingham, UK; 44 Rheumatology Research Group, Institute of Inflammation & Ageing, University of Birmingham, Birmingham, UK; 45 Rheumatology Department, Milton Keynes University Hospital, Milton Keynes, UK; 46 Rheumatology Department, The Queen Elizabeth Hospital, Woodville, South Australia; 47 University of Adelaide, Adelaide, South Australia; 48 Department of Orofacial Sciences, University of California San Francisco, San Francisco, CA USA; 49 Translational and Clinical Research Institute, Newcastle University, Newcastle upon Tyne, UK; 50 NIHR Newcastle Biomedical Centre and NIHR Newcastle Clinical Research Facility, Newcastle upon Tyne Hospitals NHS Foundation Trust, Newcastle upon Tyne, UK; 51 Center for Bioinformatics, Department of Public Health Sciences, Henry Ford Health System, Detroit, MI USA

**Keywords:** Rheumatic diseases, Rheumatic diseases, Genome-wide association studies, Genetic predisposition to disease

Correction to: *Nature Communications* 10.1038/s41467-022-30773-y, published online 27 July 2022

In this article the funding from ‘FOREUM Foundation for Research in Rheumatology’ was omitted. The original article has been corrected.

