# Supplementary material for: Genome-wide association study identifies Sjögren’s risk loci with functional implications in immune and glandular cells
Source: Nat Commun. 2022 Jul 27;13:4287. doi: 10.1038/s41467-022-30773-y (PMC9329286; doi:10.1038/s41467-022-30773-y)
Supplement: Supplementary file 1 — Supplementary Information [file 41467_2022_30773_MOESM1_ESM.pdf]

Supplementary Information for

Genome-wide association study identifies Sjögren's risk loci with functional implications in immune and glandular cells

Bhuwan Khatri[1], Kandice L. Tessneer[1], Astrid Rasmussen[1], Farhang Aghakhanian[1], Tove Ragna Reksten[2,3], Adam Adler[4], Ilias Alevizos[5], Juan-Manuel Anaya[6], Lara A. Aqrawi[7, 8], Eva Baecklund[9], Johan G. Brun[3], Sara Magnusson Bucher[10], Maija-Leena Eloranta[9], Fiona Engelke[11], Helena Forsblad-d'Elia[12], Stuart B. Glenn[1], Daniel Hammenfors[13], Juliana Imgenberg-Kreuz[9], Janicke Liaaen Jensen[7], Svein Joar Auglænd Johnsen[14], Malin V. Jonsson[3,15], Marika Kvarnström[16,17], Jennifer A. Kelly[1], He Li[2,18], Thomas Mandl[19], Javier Martín[20], Gaétane Nocturne[21], Katrine Brække Norheim[3,22], Øyvind Palm[23], Kathrine Skarstein[3,24], Anna M. Stolarczyk[1], Kimberly E. Taylor[25], Maria Teruel[26], Elke Theander[27,28], Swamy Venuturupalli[29,30], Daniel J Wallace[29,30], Kiely M. Grundahl[1], Kimberly S. Hefner[31], Lida Radfar[32], David M. Lewis[33], Donald U. Stone[34], C. Erick Kaufman[35], Michael T. Brennan[36,37], Joel M. Guthridge[2,38], Judith A. James[2,35], R. Hal Scofield[2,35,39], Patrick M. Gaffney[1], Lindsey A. Criswell[25,40,41], Roland Jonsson[3,13], Per Eriksson[42], Simon J. Bowman[43,44,45], Roald Omdal[3,14], Lars Rönnblom[9], Blake Warner[5], Maureen Rischmueller[46,47], Torsten Witte[11], A. Darise Farris[2], Xavier Mariette[21], Marta E. Alarcon-Riquelme[26], PRECISESADS Clinical Consortium, Caroline H. Shiboski[48], Sjögren's International Collaborative Clinical Alliance (SICCA), Marie Wahren-Herlenius[3,16], Wan-Fai Ng[49,50], UK Primary Sjögren's Syndrome Registry, Kathy L. Sivils[2,18], Indra Adrianto[51], Gunnel Nordmark[9], Christopher J. Lessard[1,38#]

[1]Genes and Human Disease Research Program, Oklahoma Medical Research Foundation, Oklahoma City, Oklahoma, USA.
[2]Arthritis and Clinical Immunology Research Program, Oklahoma Medical Research Foundation, Oklahoma City, Oklahoma, USA.
[3]Department of Clinical Science, University of Bergen, Bergen, Norway.
[4]NGS Core Laboratory, Oklahoma Medical Research Foundation, Oklahoma City, Oklahoma, USA.
[5]Salivary Disorder Unit, National Institute of Dental and Craniofacial Research, Bethesda, Maryland, USA.
[6]Center for Autoimmune Diseases Research (CREA), Universidad del Rosario, Bogotá, Colombia.
[7]Department of Oral Surgery and Oral Medicine, Faculty of Dentistry, University of Oslo, Oslo, Norway.
[8]Department of Health Sciences, Kristiania University College, Oslo, Norway.
[9]Department of Medical Sciences, Rheumatology and Science for Life Laboratory, Uppsala University, Uppsala, Sweden.
[10]Department of Rheumatology, Faculty of Medicine and Health, Örebro University, Örebro, Sweden.
[11]Department of Rheumatology and Immunology, Hannover Medical School, Hannover, Germany.
[12]Department of Rheumatology and Inflammation Research, Sahlgrenska Academy at University of Gothenburg, Gothenburg, Sweden.

[13]Department of Rheumatology, Haukeland University Hospital, Bergen, Norway.
[14]Department of Internal Medicine, Clinical Immunology Unit, Stavanger University Hospital, Stavanger, Norway.
[15]Section for Oral and Maxillofacial Radiology, Department of Clinical Dentistry, Medical Faculty, University of Bergen, Bergen, Norway.
[16]Rheumatology Unity, Department of Medicine, Karolinska University Hospital, Karolinska Institutet, Stockholm, Sweden.
[17]Academic Specialist Center, Center for Rheumatology and Studieenheten, Stockholm Health Services, Region Stockholm, Sweden.
[18]Translational Sciences, The Janssen Pharmaceutical Companies of Johnson & Johnson, Spring House, Pennsylvania, USA.
[19]Rheumatology, Department of Clinical Sciences Malmö, Lund University, Malmö, Sweden.
[20]Instituto de Biomedicina y Parasitología López-Neyra, Consejo Superior de Investigaciones Científicas (CSIC), Granada, Spain.
[21]Université Paris-Saclay, Assistance Publique–Hôpitaux de Paris (AP-HP), Hôpital Bicêtre, Institut National de la Santé et de la Recherche Médicale (INSERM) UMR1184, Le Kremlin Bicêtre, France.
[22]Department of Rheumatology, Stavanger University Hospital, Stavanger, Norway.
[23]Department of Rheumatology, University of Oslo, Oslo, Norway.
[24]Department of Pathology, Haukeland University Hospital, Bergen, Norway
[25]Department of Medicine, Russell/Engleman Rheumatology Research Center, University of California San Francisco, San Francisco, California, USA.
[26]Genyo, Center for Genomics and Oncological Research, Pfizer/University of Granada/Andalusian Regional Government, Granada, Spain.
[27]Department of Rheumatology, Skåne University Hospital, Malmö, Sweden.
[28]Medical Affairs, Jannsen-Cilag EMEA (Europe/Middle East/Africa), Beerse, Belgium.
[29]Division of Rheumatology, Cedars-Sinai Medical Center, Los Angeles, California, USA.
[30]David Geffen School of Medicine, University of California Los Angeles, Los Angeles California, USA.
[31]Hefner Eye Care and Optical Center, Oklahoma City, Oklahoma, USA.
[32]Oral Diagnosis and Radiology Department, University of Oklahoma College of Dentistry, Oklahoma City, Oklahoma, USA.
[33]Department of Oral and Maxillofacial Pathology, University of Oklahoma College of Dentistry, Oklahoma City, Oklahoma, USA.
[34]Department of Ophthalmology, Dean McGee Eye Institute, University of Oklahoma Health Sciences Center, Oklahoma City, Oklahoma, USA.
[35]Department of Medicine, University of Oklahoma Health Sciences Center, Oklahoma City, Oklahoma, USA.
[36]Department of Oral Medicine/Oral & Maxillofacial Surgery, Atrium Health Carolinas Medical Center, Charlotte, North Carolina, USA.
[37]Department of Otolaryngology/Head and Neck Surgery, Wake Forest University School of Medicine, Winston-Salem, North Carolina, USA.
[38]Department of Pathology, University of Oklahoma Health Sciences Center, Oklahoma City, Oklahoma, USA.
[39]US Department of Veterans Affairs Medical Center, Oklahoma City, Oklahoma, USA.

[40]Institute of Human Genetics (IHG), University of California San Francisco, San Francisco, California, USA.
[41]Genomics of Autoimmune Rheumatic Disease Section, National Human Genome Research Institute, NIH, Bethesda, Maryland, USA.
[42]Department of Biomedical and Clinical Sciences, Division of Inflammation and Infection, Linköping University, Linköping, Sweden.
[43]Rheumatology Department, University Hospital Birmingham NHS Foundation Trust, Birmingham, United Kingdom.
[44]Rheumatology Research Group, Institute of Inflammation & Ageing, University of Birmingham, Birmingham, United Kingdom.
[45]Rheumatology Department, Milton Keynes University Hospital, Milton Keynes, United Kingdom.
[46]Rheumatology Department, The Queen Elizabeth Hospital, Woodville, South Australia.
[47]University of Adelaide, Adelaide, South Australia.
[48]Department of Orofacial Sciences, University of California San Francisco, San Francisco, California, USA.
[49]Translational and Clinical Research Institute, Newcastle University, Newcastle upon Tyne, United Kingdom.
[50]NIHR Newcastle Biomedical Centre and NIHR Newcastle Clinical Research Facility, Newcastle upon Tyne Hospitals NHS Foundation Trust, Newcastle upon Tyne, United Kingdom.
[51]Center for Bioinformatics, Department of Public Health Sciences, Henry Ford Health System, Detroit, Michigan, USA.
[#]Corresponding Author: chris-lessard@omrf.org (ORCID ID: https://orcid.org/0000-0003-2440-3843)

Table of Contents: **Page**

i. Consortium Acknowledgments and Funding*…………………………… 5

ii. Supplementary Figures and Legends

a. Supplementary Figure 1 ……………………………………………… 9
b. Supplementary Figure 2 ……………………………………………… 11
c. Supplementary Figure 3 ……………………………………………… 13
d. Supplementary Figure 4 ……………………………………………… 16
e. Supplementary Figure 5 ……………………………………………… 22
f. Supplementary Figure 6 ……………………………………………… 28
g. Supplementary Figure 7 ……………………………………………… 37
h. Supplementary Figure 8 ……………………………………………… 43
i. Supplementary Figure 9 ……………………………………………… 49
j. Supplementary Figure 10 ……………………………………………...58
k. Supplementary Figure 11……………………………………………... 63
l. Supplementary Figure 12 …………………………………………….. 72
m. Supplementary Figure 13 ……………………………………………...79

Consortium Acknowledgments and Funding*:

A. The PRECISESADS Clinical Consortium is composed of the following members:

Lorenzo Beretta[1], Barbara Vigone[1], Jacques-Olivier Pers[2], Alain Saraux[2], Valérie Devauchelle-Pensec[2], Divi Cornec[2], Sandrine Jousse-Joulin[2], Bernard Lauwerys[3], Julie Ducreux[3], Anne-Lise Maudoux[3], Carlos Vasconcelos[4], Ana Tavares[4], Esmeralda Neves[4], Raquel Faria[4], Mariana Brandão[4], Ana Campar[4], António Marinho[4], Fátima Farinha[4], Isabel Almeida[4], Miguel Angel Gonzalez-Gay Montecón[5], Ricardo Blanco Alonso[5], Alfonso Corrales Martinez[5], Ricard Cervera[6], Ignasi Rodríguez-Pintó[6], Gerard Espinosa[6], Rik Lories[7], Ellen De Langhe[7], Nicolas Huzelmann[8], Doreen Belz[8], Torsten Witte[9], Niklas Baerlecken[9], Georg Stummvoll[10], Michael Zauner[10], Michaela Lehner[10], Eduardo Collantes[11], Rafaela Ortega-Castro[11], Mª Angeles Aguirre-Zamorano[11], Alejandro Escudero-Contreras[11], Mª Carmen Castro-Villegas[11], Norberto Ortego[12], María Concepción Fernández Roldán[12], Enrique Raya[13], Immaculada Jiménez Moleón[13], Enrique de Ramon[14], Isabel Díaz Quintero[14], Pier Luigi Meroni[15], Maria Gerosa[15], Tommaso Schioppo[15], Carolina Artusi[15], Carlo Chizzolini[16], Aleksandra Zuber[16], Donatienne Wynar[16], Laszló Kovács[17], Attila Balog[17], Magdolna Deák[17], Márta Bocskai[17], Sonja Dulic[17], Gabriella Kádár[17], Falk Hiepe[18], Velia Gerl[18], Silvia Thiel[18], Manuel Rodriguez Maresca[19], Antonio López-Berrio[19], Rocío Aguilar-Quesada[19], Héctor Navarro-Linares[19], and Marta E. Alarcon-Riquelme[20].

[1]Referral Center for Systemic Autoimmune Diseases, Fondazione IRCCS Ca' Granda Ospedale Maggiore Policlinico di Milano, Italy; [2]Centre Hospitalier Universitaire de Brest, Hospital de la Cavale Blanche, Brest, France; [3]Pôle de pathologies rhumatismales systémiques et inflammatoires, Institut de Recherche Expérimentale et Clinique, Université catholique de Louvain, Brussels, Belgium; [4]Centro Hospitalar do Porto, Portugal; [5]Servicio Cantabro de Salud, Hospital Universitario Marqués de Valdecilla, Santander, Spain; [6]Hospital Clinic I Provicia, Institut d'Investigacions Biomèdiques August Pi i Sunyer, Barcelona, Spain; [7]Katholieke Universiteit Leuven, Belgium; [8]Klinikum der Universitaet zu Koeln, Cologne, Germany; [9]Medizinische Hochschule Hannover, Germany; [10]Medical University Vienna, Vienna, Austria; [11]Servicio Andaluz de Salud, Hospital Universitario Reina Sofía Córdoba, Spain; [12]Servicio Andaluz de Salud, Complejo hospitalario Universitario de Granada (Hospital Universitario San Cecilio), Spain; [13]Servicio Andaluz de Salud, Complejo hospitalario Universitario de Granada (Hospital Virgen de las Nieves), Spain; [14]Servicio Andaluz de Salud, Hospital Regional Universitario de Málaga, Spain; [15]Università degli studi di Milano, Milan, Italy; [16]Hospitaux Universitaires de Genève, Switzerland; [17]University of Szeged, Szeged, Hungary; [18]Charite, Berlin, Germany; [19]Andalusian Public Health System Biobank, Granada, Spain; [20]Genyo, Center for Genomics and Oncological Research, Pfizer/University of Granada/Andalusian Regional Government, Granada, Spain.

The study was approved by the following ethic committees: Comitato Etico Area 2 (Fondazione IRCCS Ca' Granda Ospedale Maggiore Policlinico di Milano and University of Milan); approval no. 425bis Nov 19, 2014, and no. 671_2018 Sep 19, 2018; Klinikum der Universitaet zu Koeln, Cologne, Germany. Geschaftsstelle Ethikkommission; Pôle de pathologies rhumatismales systémiques et inflammatoires, Institut de Recherche Expérimentale et Clinique, Université catholique de Louvain, Brussels, Belgium. Comité d`Èthique Hospitalo-Facultaire; University of Szeged, Szeged, Hungary. Csongrad Megyei Kormanyhivatal; Hospital Clinic I Provicia, Institut d'Investigacions Biomèdiques August Pi i Sunyer, Barcelona, Spain. Comité Ética de Investigación Clínica del Hospital Clínic de Barcelona. Hospital Clinic del Barcelona; Servicio Andaluz de Salud, Hospital Universitario Reina Sofía Córdoba, Spain. Comité de Ética e la Investigación de Centro de Granada (CEI – Granada); Centro Hospitalar do Porto, Portugal.

Comissao de ética para a Saude – CES do CHP; Centre Hospitalier Universitaire de Brest, Hospital de la Cavale Blanche, Avenue Tanguy Prigent 29609, Brest, France. Comite de Protection des Personnes Ouest VI; Hospitaux Universitaires de Genève, Switzerland. DEAS – Commission Cantonale d'ethique de la recherche Hopitaux universitaires de Geneve; Andalusian Public Health System Biobank, Granada, Spain; Katholieke Universiteit Leuven, Belgium. Commissie Medische Ethiek UZ KU Leuven /Onderzoek; Charite, Berlin, Germany. Ethikkommission; Medizinische Hochschule Hannover, Germany. Ethikkommission.

PRECISESADS Study was funded by the Innovative Medicines Initiative of the European Union with grant number 115565 partly supported by the EFPIA Companies (Alarcon-Riquelme).

B. Sjögren's International Collaborative Clinical Alliance (SICCA) is composed of the following members: Cox D[1], Jordan R[1], Lee D[1], DeSouza Y[1], Drury D[1], Do A[1], Scott L[1], Nespeco J[1], Whiteford J[1], Margaret M[1], Sack S[1], Adler I[2], Smith AC[2], Bisio AM[2], Gandolfo MS[2], Chirife AM[2], Keszler A[2], Daverio S[2], Kambo V[2], Dong Y[3], Jiang Y[3], Xu D[3], Su J[3], Du D[3], Wang H[3], Li Z[3], Xiao J[3], Wu Q[3], Zhang C[3], Meng W[3], Zhang J[3], Johansen S[4], Hamann S[4], Schiødt J[4], Holm H[4], Ibsen P[4], Manniche AM[4], Kreutzmann SP[4], and Villadsen J[4], Sugai S[5], Masaki Y[5], Sakai T[5], Shibata N[5], Honjo M[5], Kurose N[5], Nojima T[5], Kawanami T[5], Sawaki T[5], Fujimoto K[5], Odell E[6], Morgan P[6], Fernandes-Naglik L[6], Varghese-Jacob B[6], Ali S[6], Adamson M[6], Seghal S[7], Mishra R[7], Bunya V[7], Massaro-Giordano M[7], Abboud SK[7], Pinto A[7], Sia YW[7], Dow K[7], Akpek E[8], Ingrodi S[8], Henderson W[8], Gourin C[8], Keyes A[8], Srinivasan M[9], Mascarenhas J[9], Das M[9], Kumar A[9], Joshi P[9], Banushree R[9], Kim U[9], Babu B[9], Ram A[9], Saravanan R[9], Kannappan KN[9], Kalyani N[9], Criswell LA[1], Shiboski SC[1], Baer A[8], Challacombe S[6], Lanfranchi H[2], Schiødt M[4], Umehara H[5], Vivino F[7], Zhao Y[3], Dong Y[3], Greenspan D[1], Heidenreich AM[2], Helin P[4], Kirkham B[6], Kitagawa K[5], Larkin G[6], Li M[3], Lietman T[1], Lindegaard J[4], McNamara N[1], Sack K[1], Shirlaw P[6], Sugai S[5], Vollenweider C[2], Whitcher J[1], Wu A[1], Zhang S[3], Zhang W[3], Greenspan JS[1], Daniels TE[1], Shiboski CH[1], Criswell LA[10].

[1]University of California San Francisco, San Francisco, CA, USA; [2]University of Buenos Aires and German Hospital, Buenos Aires, Argentina; [3]Peking Union Medical College Hospital, Beijing, China; [4]Rigshospitalet, Copenhagen, Denmark; [5]Kanazawa Medical University, Ishikawa, Japan; [6]King's College London, London, UK; [7]University of Pennsylvania, Philadelphia, Pennsylvania, USA; [8]Johns Hopkins University, Baltimore, Maryland, USA; [9]Aravind Eye Hospital, Madurai, India; [10]National Human Genome Research Institute, NIH, Bethesda, Maryland, USA.

SICCA Study was funded by the National Institutes of Health (NIH): N01DE32636 (SICCA), HHSN26S201300057C (SICCA), U01DE028891 (SICCA), R03DE029800 (SICCA), U01HG004446 (SICCA-GWAS), P30AR070155 (SICCA-GWAS).

Genotype data from the Sjögren's International Collaborative Clinical Alliance (SICCA) Registry was obtained through **dbGAP accession number phs000672.v1.p1**. This study was supported by the National Institute of Dental and Craniofacial Research (NIDCR), the National Eye Institute, and the Office of Research on Women's Health through contract number N01-DE-32636. Genotyping services were provided by the Center for Inherited Disease Research (CIDR). CIDR is fully funded through a federal contract from the National Institutes of Health (NIH) to the Johns Hopkins University (contract numbers HHSN268200782096C, HHSN268201100011I, HHSN268201200008I). Funds for genotyping were provided by the NIDCR through CIDR's NIH contract. Assistance with

data cleaning and imputation was provided by the University of Washington. SICCA thanks investigators from the following studies that provided DNA samples for genotyping: the Genetic Architecture of Smoking and Smoking Cessation, Collaborative Genetic Study of Nicotine Dependence (phs000404.v1.p1); Age-Related Eye Disease Study (AREDS) - Genetic Variation in Refractive Error Substudy (phs000429.v1.p1); and National Institute of Mental Health's Human Genetics Initiative (phs000021.v3.p2, phs000167.v1.p1). SICCA thanks the many clinical collaborators and research participants who contributed to this research.

C. The UK Primary Sjögren's Syndrome Registry is composed of the following members: Wan-Fai Ng[1], Simon J. Bowman[2], Bridget Griffiths[3], Frances Hall[4], Elalaine C. Bacabac[5], Robert Moots[5], Kuntal Chadravarty[6], Shamin Lamabadusuriya[6], Michele Bombardieri[7], Constantino Pitzalis[7], Nurhan Sutcliffe[7], Nagui Gendi[8], Rashidat Adeniba[8], John Hamburger[9], Andrea Richards[9], Saaeha Rauz[10], Sue Brailsford[1], Joanne Logan[11], Diamuid Mulherin[11], Paul Emery[12], Alison McManus[12], Colin Pease[12], Alison Booth[13], Marian Regan[13], Theodoros Dimitroulas[14], Lucy Kadiki[14], Daljit Kaur[14], George Kitas[14], Mark Lloyd[15], Lisa Moore[15], Esther Gordon[16], Cathy Lawson[16], Monica Gupta[17], John Hunter[17], Lesley Stirton[17], Gill Ortiz[18], Elizabeth Price[18], Gavin Clunie[19], Ginny Rose[19], Sue Cuckow[19], Susan Knight[20], Deborah Symmons[20], Beverley Jones[20], Shereen Al-Ali[1], Andrew Carr[1], Katherine Collins[1], Andini Natasari[1], Philip Stocks[1], Jessica Tarn[1], Ian Corbett[3], Christine Downie[3], Suzanne Edgar[3], Marco Carrozzo[3], Francisco Figuereido[3], Heather Foggo[3], Dennis Lendrem[3], Iain Macleod[3], Philip Mawson[3], Sheryl Mitchell[3], Adrian Jones[21], Peter Lanyon[21], Alice Muir[21], Paula White[22], Steven Young-Min[22], Susan Pugmire[23],Saravanan Vadivelu[23], Annie Cooper[24], Marianne Watkins[24], Anne Field[25], Stephen Kaye[25], Devesh Mewar[25], Patricia Medcalf[25], Pamela Tomlinson[25], Debbie Whiteside[25], Neil McHugh[26], John Pauling[26], Julie James[26], Nike Olaitan[26], Mohammed Akil[27], Jayne McDermott[27], Olivia Godia[27], David Coady[28], Elizabeth Kidd[28], Lynne Palmer[28], Bhaskar Dasgupta[29], Victoria Katsande[29], Pamela Long[29], Charles Li[30], Usha Chandra[31], Kirsten MacKay[31], Stefano Fedele[32], Ada Ferenkey-Koroma[32], Ian Giles[32], David Isenberg[32], Helena Maconnell[32], Stephen Porter[32],Paul Allcoat[33], John Mclaren[33].

[1]Newcastle University, Newcastle upon Tyne, UK; [2]University Hospital Birmingham, Birmingham,UK; [3]Newcastle upon Tyne Hospitals NHS Foundation Trust, Newcastle upon Tyne, UK; [4]Addenbrooke's Hospital, Cambridge, UK; [5]Aintree University Hospitals, Liverpool, UK; [6]Barking, Havering and Redbridge NHS Trust, Barking, UK; [7]Bart and the London NHS Trust, London, UK; [8]Basildon Hospital, Basildon, UK; [9]Birmingham Dental Hospital, Brimingham, UK; [10]Birmingham & Midland Eye Centre, Birmingham, UK; [11]Cannock Chase Hospital, Cannock, UK; [12]Chapel Allerton Hospital, Leeds, Leeds, UK; [13]Derbyshire Royal Infirmary, Derby, UK; [14]Dudley Group of Hospitals NHS Foundation Trust, Dudley, UK; [15]Frimley Park Hospital, Frimley Park, UK; [16]Harrogate District Foundation Trust Hospital, Harrogate, UK; [17]Gartnavel General Hospital, Glasgow, UK; [18]Great Western Hospital, Swindon, UK; [19]Ipswich Hospital NHS Trust, Ipswich, UK; [20]Macclesfield District General Hospital & Arthritis Research UK Epidemiology Unit, Manchester, Manchester, UK; [21]Nottingham University Hospital, Nottingham, UK; [22]Portsmouth Hospitals NHS Trust, Portsmouth, UK; [23]Queen's Elizabeth Hospital, Gateshead, Gateshead, UK; [24]Royal Hampshire County Hospital, Winchester, UK; [25]Royal Liverpool University Hospital, Liverpool, UK; [26]Royal National Hospital for Rheumatic Diseases, Bath, UK; [27]Sheffield Teaching Hospitals NHS Trust, Sheffield, UK; [28]Sunderland Royal Hospital, Sunderland, UK; [29]Southend University Hospital, Southend, UK; [30]Royal Surrey Hospital, Guildford, UK; [31]Torbay Hospital, Torbay, UK; [32]University College Hospital & Eastman Dental Institute, London, UK; [33]Whyteman's Brae Hospital, Kirkaldy, Fife, UK.

The UK Primary Sjögren's Syndrome Registry was funded by the Medical Research Council (G080062; W-F.N.), and the British Sjögren's Syndrome Association (W-F.N.). This work also received infra-structure support from the NIHR Newcastle Biomedical Research Centre, Newcastle and NIHR Newcastle Clinical Research Facility.

SUPPLEMENTAL FIGURE LEGENDS

Supplementary Figure 1: Genome-wide association study (GWAS) and meta-analysis of genotyped Sjögren's datasets

(a) Principal component (PC) analysis was done using EIGENSTRAT and 1000 Genomes Project reference population. Distribution of individual Sjögren's cases (red) and population controls (blue), along with reference European subjects from the 1000 Genomes Project population, are shown along PC1 and PC2. Ancestry of individuals from the 1000 Genomes Project population are indicated as follows: CEU: Utah Residents (CEPH) with Northern and Western European Ancestry (green), TSI: Toscani in Italy (yellow), FIN: Finnish in Finland (purple), GBR: British in England and Scotland (black), IBS: Iberian Population in Spain (orange).

(b) Distribution of individual Sjögren's cases and population controls from the six genotyped cohorts from this study shown along PC1 and PC2: Phase1 (Dataset 1), Scandinavian-1 (Dataset 2), Non-Scandinavian (Dataset 3), SICCA (Dataset 4), Scandinavian-2 (Dataset 5), PRECISESADS (Dataset 6) (Figure 1; Supplementary Table 2).

(c) Distribution of individual Sjögren's cases (red) and population controls (blue) along PC1 and PC2.

(d) Screeplot of 10 PCs.

(e) Quantile-Quantile (QQ) plot of observed p-values verses expected p-values ($-\log_{10}$ scale) for all tested Sjögren-SNPs. Dotted black line indicates expected distribution of p-values. λ denotes the inflation factor.

(f) Quantile-Quantile (QQ) plot of observed p-values verses expected p-values ($-\log_{10}$ scale) for all tested Sjögren-SNPs after exclusion of the previously identified regions of association: *HLA*, *IL12A*, *TNIP1*, *STAT1-STAT4*, *IRF5-TNPO3*, *FAM167A-BLK*, and *DDX6-CXCR5*. Dotted black line indicates expected distribution of p-values. λ denotes the inflation factor.

(g) Manhattan plot shows the summary data from the GWAS results for the 6.2×10^6 SNPs overlapping the six genotyped European datasets after imputation. The $-\log_{10}(P)$ for each variant is plotted according to chromosome and base pair position. A total of seven novel loci (indicated by red dots) exceeded genome-wide significance of $P_{GWAS}<5\times10^{-8}$ (red dashed line). Several previously established loci were replicated (indicated by light blue dots). The suggestive GWAS threshold ($P_{Suggestive}<5\times10_{-5}$) is indicated by the blue dashed line.

(h) Principal component analysis was done using EIGENSTRAT and 1000 Genomes Project reference population. Distribution of individual Sjögren's cases (red) and population controls (blue) from the ImmunoChip (Dataset 7) along PC1 and PC2 (Figure 1; Supplementary Table 1).

(i) Screeplot of 10 PCs.

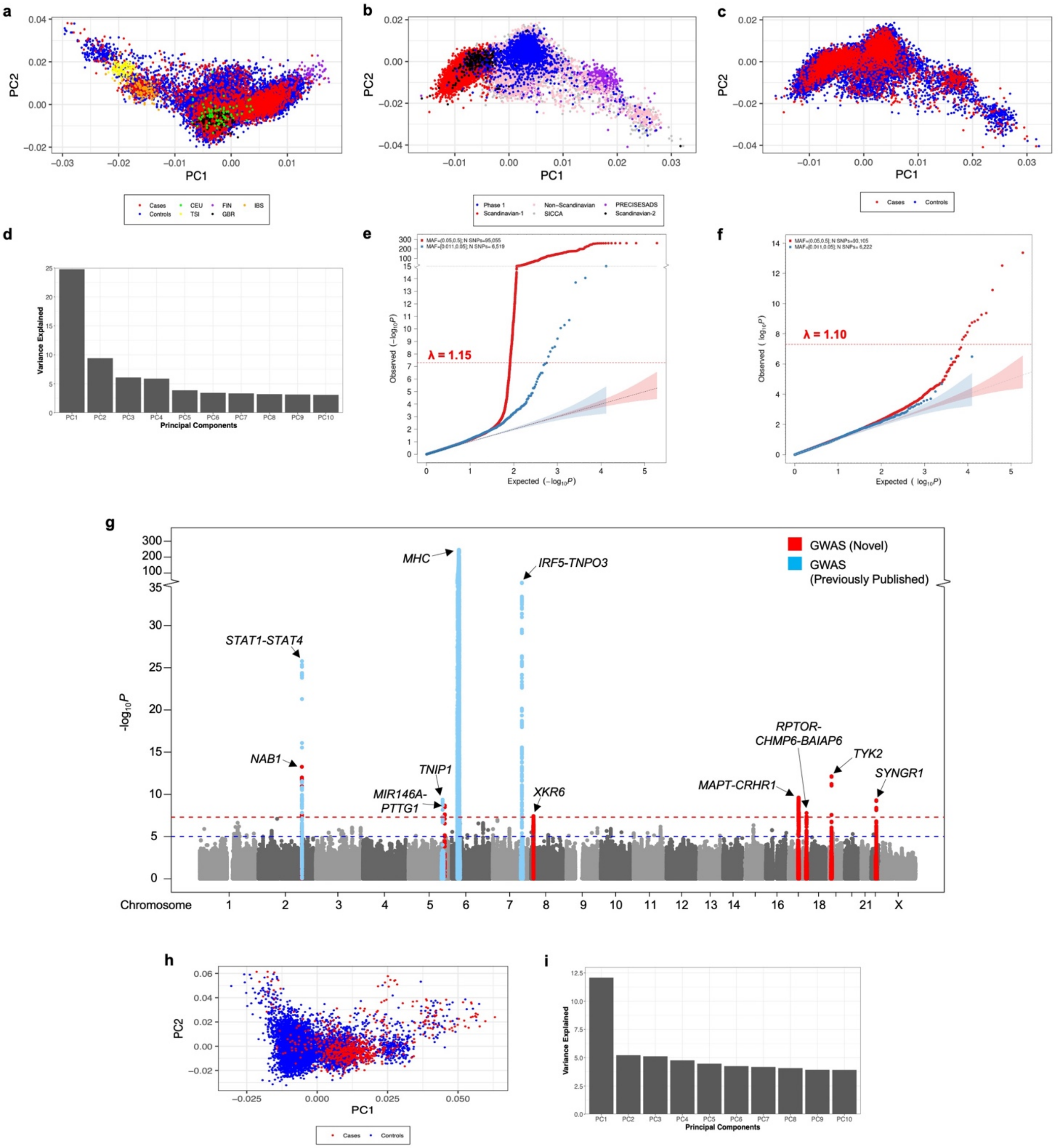

a
b
c
d
e
f
g
h
i
PC1
PC2
Cases
Controls
CEU
TSI
FIN
GBR
IBS
Phase 1
Scandinavian-1
Non-Scandinavian
SICCA
PRECISESADS
Scandinavian-2
Variance Explained
Principal Components
λ = 1.15
λ = 1.10
Expected
Observed
MHC
IRF5-TNPO3
STAT1-STAT4
NAB1
TNIP1
MIR146A-PTTG1
XKR6
MAPT-CRHR1
RPTOR-CHMP6-BAIAP6
TYK2
SYNGR1
GWAS (Novel)
GWAS (Previously Published)
Chromosome

Supplementary Figure 2: Polygenic risk score (PRS) analysis of genotyped Sjögren's individuals.

(a, b) The PI1 genotyped dataset was randomly split into 2/3rd for training and 1/3rd for testing the polygenic risk score (PRS) prediction. Principal component (PC) analysis was performed using EIGENSTRAT. Distribution of individual Sjögren's cases (red) and population control subjects (blue) along PC1 and PC2 for the (**a**) training dataset (n=2,166 cases; n=11,638 population controls) and (**b**) testing dataset (n=1,076 cases; n=5,826 population controls).

(c) Manhattan plot shows summary data of $6.2x10^6$ genotyped SNPs used in PRSice-2 to calculate PRS. The summary data was generated performing logistic regression analysis and adjusting for first 3 principal components. The $–\log_{10}(P)$ for each variant is plotted according to chromosome and base pair position. Red dashed line indicates genome-wide significance (GWS) threshold of P_{GWAS}<$5x10^{-8}$. Blue dotted line indicates a suggestive threshold of $P_{Suggestive}$ <$5x10^{-5}$.

(d) Manhattan plot shows the $4.5x10^5$ SNPs used in the PRS analysis after pruning using independent pairwise analysis with a window of 50 kb, step size, or SNPs count of 5, and r^2 >0.2 in PLINK to remove highly correlated SNPs. The summary data was generated performing logistic regression analysis and adjusting for first 3 principal components. The $–\log_{10}(P)$ for each SNP is plotted according to chromosome and base pair position. Red dashed line indicates a threshold of P_{GWAS} <5x10-8. Blue dotted line indicates a suggestive threshold of $P_{Suggestive}$ <$5x10^{-5}$.

(e) High-resolution plot showing multiple P-value thresholds (P_T) for PRS predicting Sjögren's in all genotyped individuals using all genotyped SNPs after pruning to remove highly correlated SNPs (r^2 >0.2).

(f) High-resolution plot showing multiple P-value thresholds (P_T) for PRS predicting Sjögren's in genotyped individuals after LD pruning and removal of SNPs positioned in the *HLA* region.

(g) Manhattan plot shows summary data of $6.2x10^6$ genotyped SNPs used in PRSice-2 to calculate PRS for Ro^+ Sjögren's cases relative to population controls. The $-\log_{10}(P)$ for each variant is plotted according to chromosome and base pair position. Red dashed line indicates a threshold of P_{GWAS} <$5x10^{-8}$. Blue dotted line indicates a suggestive threshold of $P_{Suggestive}$ <$5x10^{-5}$.

(h) Manhattan plot shows the $8.81x10^5$ SNPs used in the PRS analysis of Ro^+ Sjögren's cases relative to population controls after pruning using independent pairwise analysis with a window of 50 kb, step size, or SNP count of 5, and r^2 >0.2 in PLINK to remove highly correlated SNPs. The $–\log_{10}(P)$ for each SNP is plotted according to chromosome and base pair position. Red dashed line indicates a threshold of P_{GWAS}<5x10-8. Blue dotted line indicates a suggestive threshold of $P_{Suggestive}$ <$5x10^{-5}$.

(i) High-resolution plot showing multiple P-value thresholds (P_T) for PRS predicting Sjögren's in genotyped Ro^+ Sjögren's individuals using all genotyped SNPs after pruning to remove highly correlated SNPs (r^2 >0.2).

(j) High-resolution plot showing multiple P-value thresholds (P_T) for PRS predicting Sjögren's in genotyped Ro^+ Sjögren's individuals after LD pruning and removal of SNPs positioned in the *HLA* region.

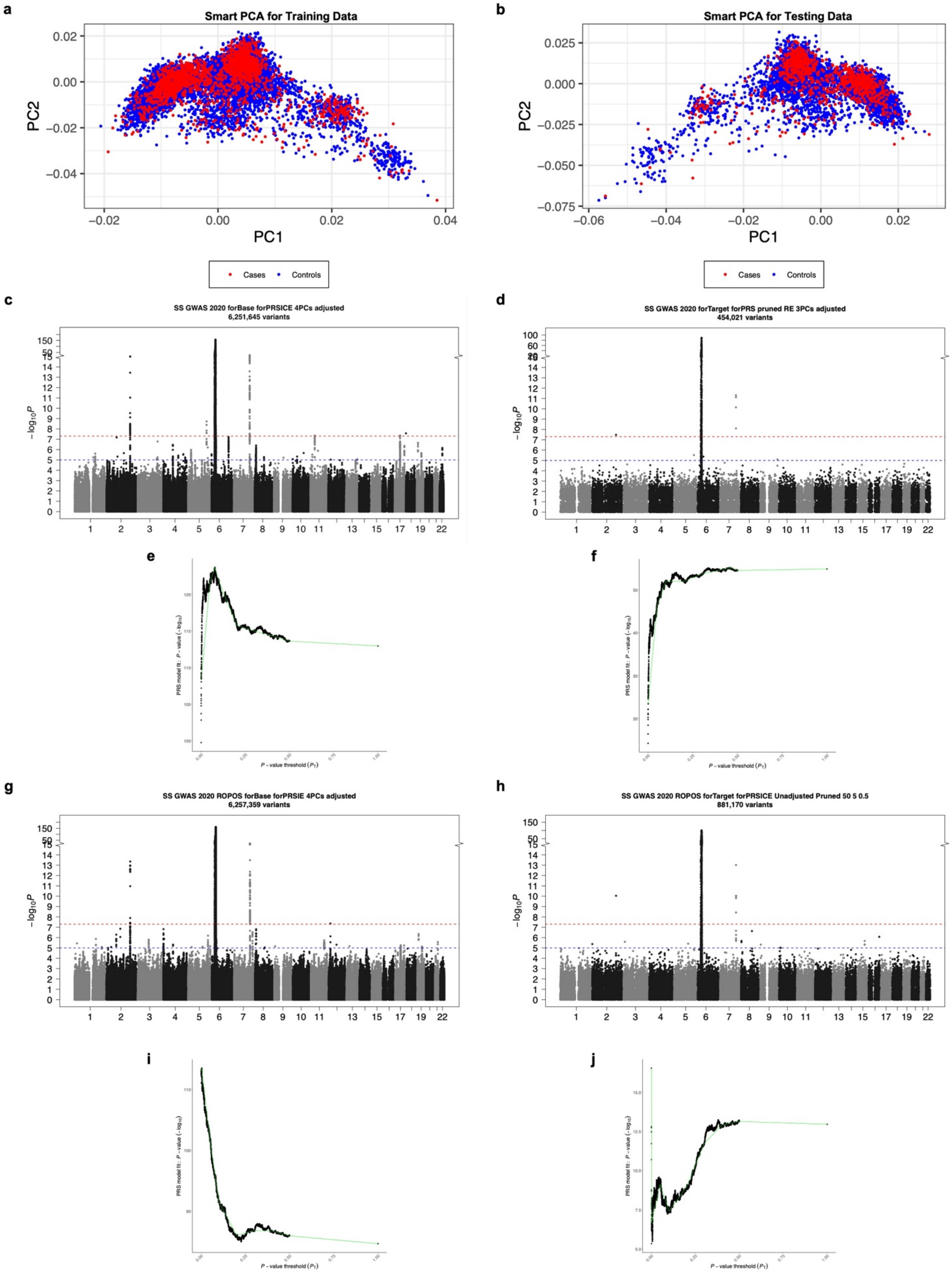

a
Smart PCA for Training Data
PC1
PC2
Cases
Controls
b
Smart PCA for Testing Data
PC1
PC2
Cases
Controls
c
SS GWAS 2020 forBase forPRSICE 4PCs adjusted
6,251,645 variants
d
SS GWAS 2020 forTarget forPRS pruned RE 3PCs adjusted
454,021 variants
e
f
PRS model fit: P-value (-log10)
P-value threshold (PT)
g
SS GWAS 2020 ROPOS forBase forPRSIE 4PCs adjusted
6,257,359 variants
h
SS GWAS 2020 ROPOS forTarget forPRSICE Unadjusted Pruned 50 5 0.5
881,170 variants
i
j

Supplementary Figure 3: Conditional analysis, posterior probability analysis, chromatin looping, and eQTL mapping in the *MAPT-CRHR1* locus.

(a) Conditional analysis was performed, adjusting for rs7210219. Residual effect was observed for rs71375320.

(b) Posterior probabilities distribution of variants in the *MAPT-CRHR1* locus, identifying rs7210219 as the most probable SNP.

(c-e) The pairwise *D'* (**c**), r^2 (**d**), and haplotypes of the *MAPT-CRHR1* locus with the frequencies of the top and functional variants in the region displayed (**e**).

(f) Circos plot mapping the zoom regional Manhattan plot of the imputed GWAS data for the *MAPT-CRHR1* region on Chromosome 17 (outer most layer). All SNPs with a $-\log_{10}$(p-value) <0.05 are shown in black or colored based on r^2 (red: r^2>0.08; orange: r^2>0.06). The index SNP of the *MAPT-CRHR1* association, rs7210219, is indicated. The outer circle displays the chromosome coordinate of the *MAPT-CRHR1* risk locus highlighted in royal blue. Genes that are eQTLs or exhibit chromatin interaction by Hi-C in GM12878 Epstein-Barr virus (EBV)-transformed B lymphocytes are reported on the inner circles in green or orange font, respectively, and are shown as links colored green or orange, respectively, on the inner most layer.

(g) EcholocatoR was used to identify, fine-map and annotate the *CRHR1* region after specifying the index SNP (indicated by red dotted line): rs7210219. Additional SNPs with plausible function were identified and indicated by the dotted yellow lines.

a b

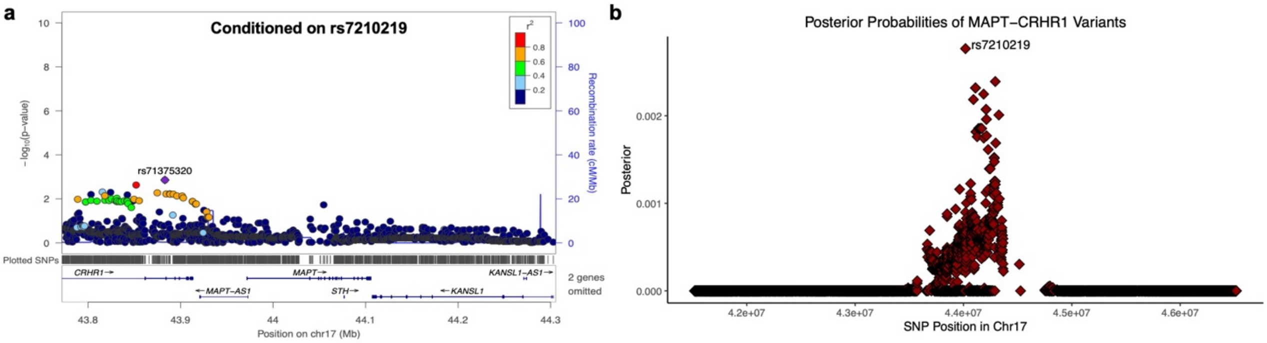

c d

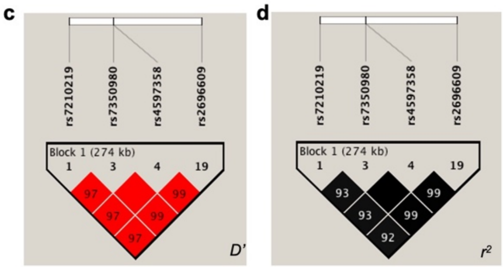

e

rs7210219	rs7350980	rs4597358	rs2696609	Haplotype frequency Overall	Case	Control	χ^2	P
(blue)				0.796	0.820	0.791	25.96	2.07E-07
(red)				0.193	0.170	0.198	24.13	2.47E-07

f

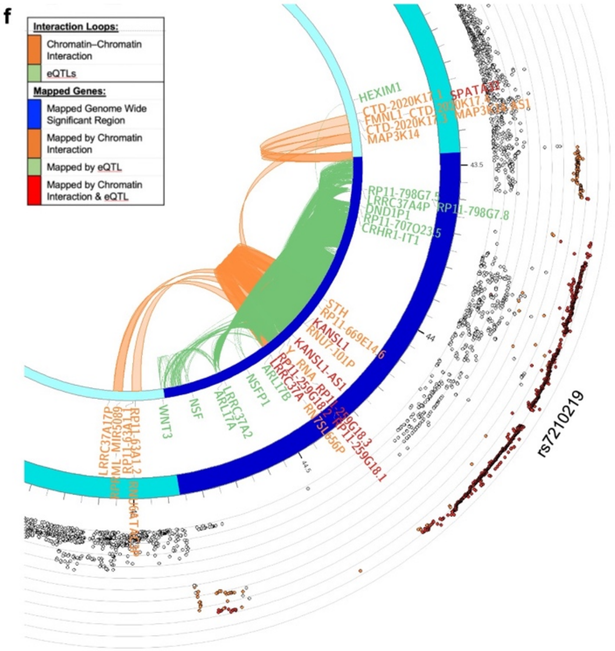

g

EcholocatoR Analyses: rs7210219 (See next page)

CRHR1_rs7210219

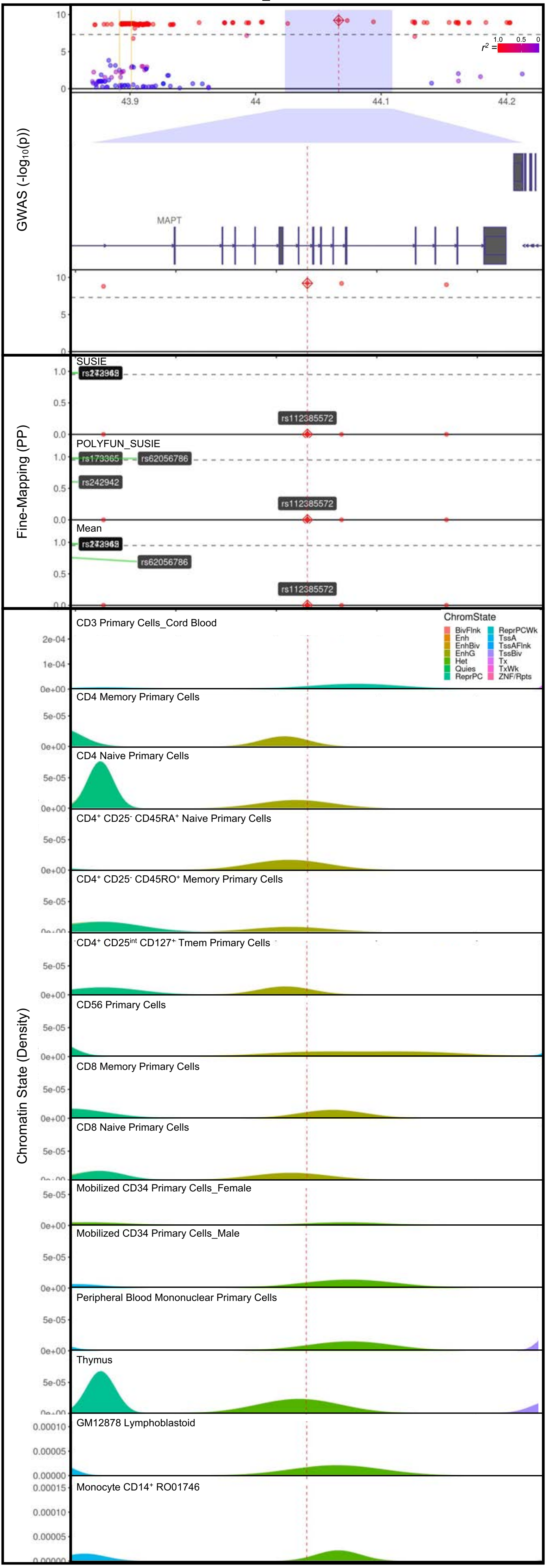

Supplementary Figure 4: Conditional analysis, posterior probability analysis, chromatin looping, and eQTL mapping in the *CD247* locus.

(a) Conditional analysis was performed, adjusting for rs7523907. Residual effect was observed for rs6427098.

(b) Posterior probabilities distribution of variants in *CD247* locus, identifying rs7523907 as the most probable SNP.

(c-e) The pairwise *D'* (**c**), r^2 (**d**), and haplotypes with the frequencies of the top and functional variants in the region are displayed (**e**).

(f) Circos plot mapping the zoom regional Manhattan plot of the imputed GWAS data for the *CD247* association on Chromosome 1 (outer most layer). All SNPs with a $-\log_{10}$(p-value) <0.05 are shown in black, or colored based on r^2 (red: r^2>0.08; orange: r^2>0.06). The index SNP of the *CD247* association, rs7523907, is indicated. The outer circle displays the chromosome coordinate of the *CD247* risk locus highlighted in blue. Genes that are eQTLs or exhibit chromatin interaction by Hi-C in GM12878 Epstein-Barr virus (EBV)-transformed B lymphocytes are reported on the inner circles in green or orange font, respectively, and are shown as links colored green or orange, respectively, on the inner most layer.

(g-i) Sjögren-SNPs rs7523907 (**g**), rs2949661 (**h**), and rs1214595 (**i**), positioned in an intronic enhancer of *CD247*, exhibited strong epigenetic enhancer and promoter marks in several immune cell types (rectangles). Coalescence of chromatin-chromatin interactions (purple triangles) and several common eQTLs (yellow, orange, blue triangles), suggest that the intronic enhancer regulates the promoters of *CD247* (T cell receptor zeta chain) and *CREG1* (adenovirus E1A protein that promotes cell proliferation). rs2949661 (**h**) and rs1214595 (**i**) are also eQTLs for *TIPRL* (inhibitory regulator of protein phosphatase-2A (PP2A) in the pro-apoptotic TOR signaling pathway) or *POGK* in the minor salivary gland, respectively. For details, see Supplementary Table 10.

(j) Sjögren-SNP rs1723018, positioned in the intronic enhancer of *CD247*, has epimarks consistent with enhancer and promoter activity in $CD4^+$ and $CD8^+$ T cells (rectangles). Coalescence of chromatin-chromatin interaction (purple triangles) and eQTL data (top blue and yellow triangles) suggest that the enhancer likely regulates the promoter of *CD247* specifically in T cells. For details, see Supplementary Table 10.

(k-n) EcholocatoR was used to identify, fine-map and annotate the *CD247* region after specifying the index SNPs (indicated by red dotted line): rs7523907 (**k**), rs2949661 (**l**), rs1214595 (**m**), rs1723018 (**n**). Additional SNPs with plausible function were identified and indicated by the dotted yellow lines.

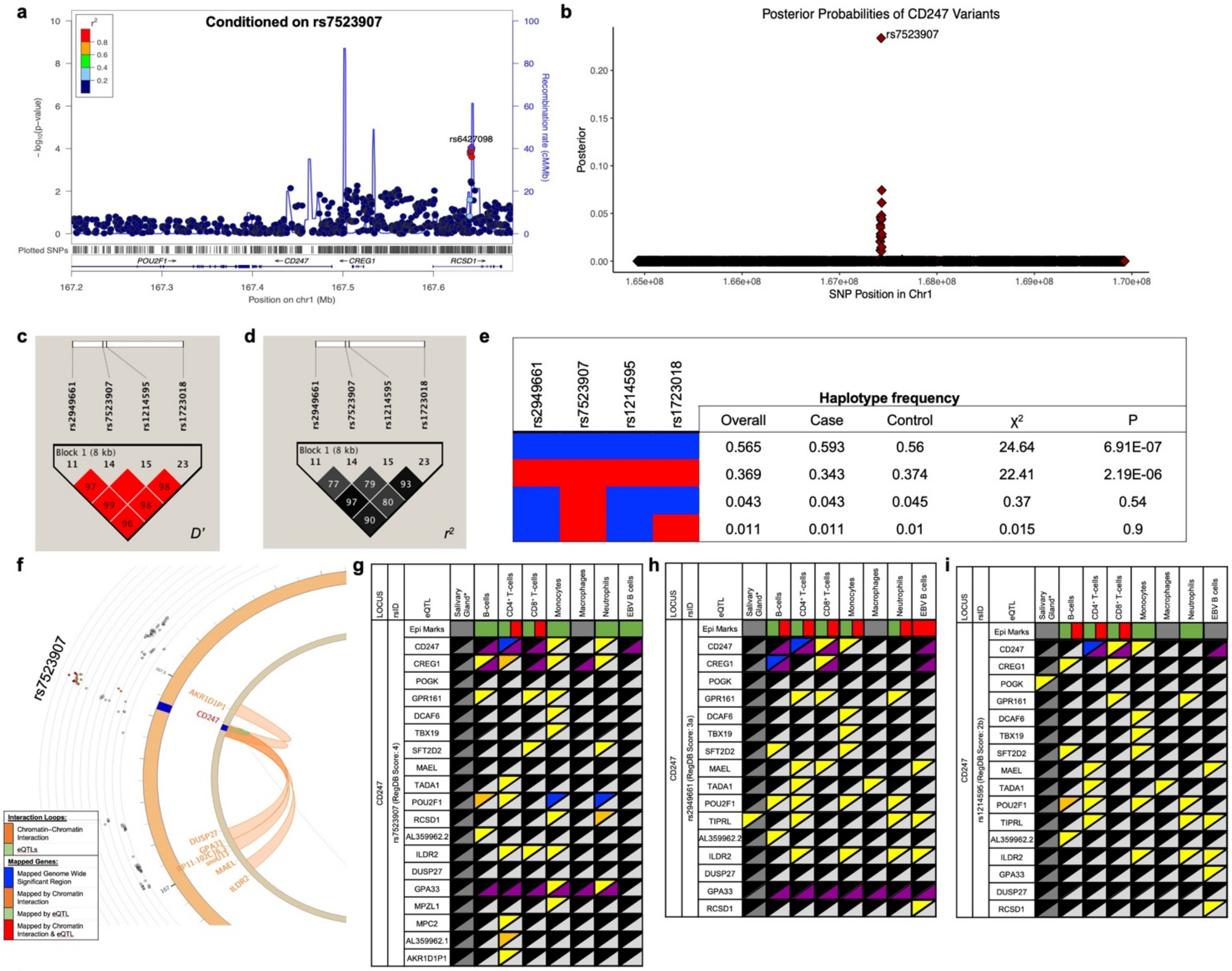

rs2949661 / rs7523907 / rs1214595 / rs1723018	Overall	Case	Control	χ^2	P
	0.565	0.593	0.56	24.64	6.91E-07
	0.369	0.343	0.374	22.41	2.19E-06
	0.043	0.043	0.045	0.37	0.54
	0.011	0.011	0.01	0.015	0.9

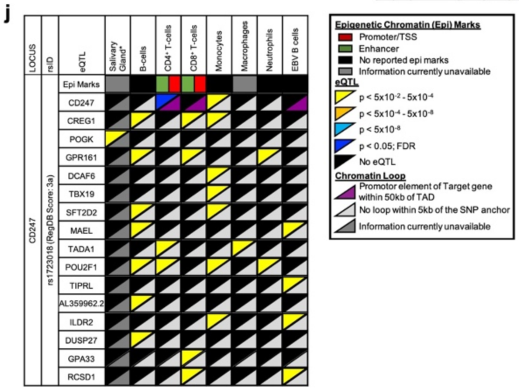

k*) EcholocatoR Analyses: rs7523907
l*) EcholocatoR Analyses: rs2949661
m*) EcholocatoR Analyses: rs1214595
n*) EcholocatoR Analyses: rs1723018

*see next page

CD247_rs7523907

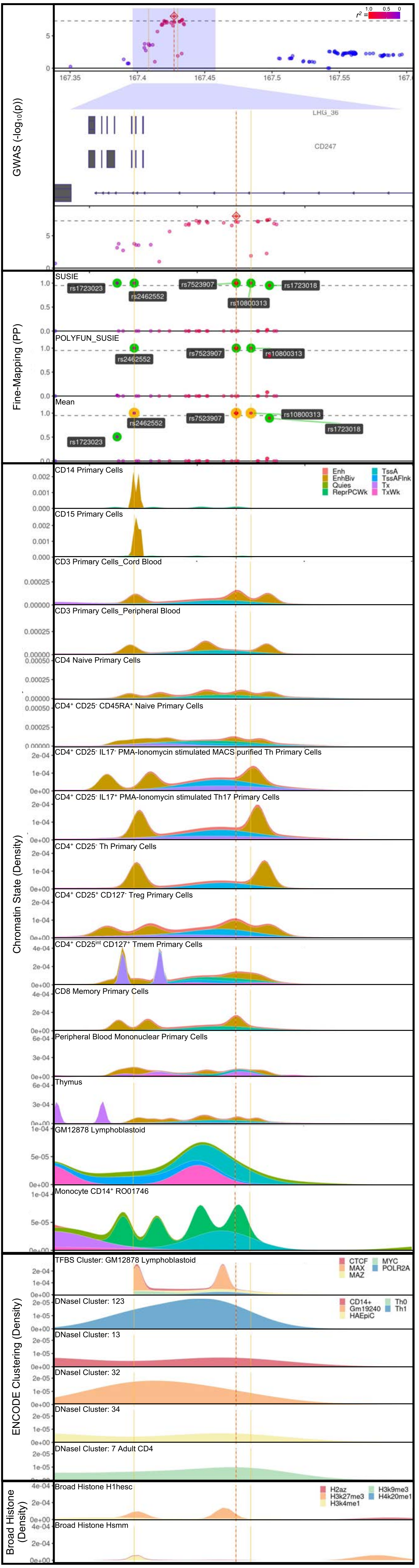

CD247_rs2949661

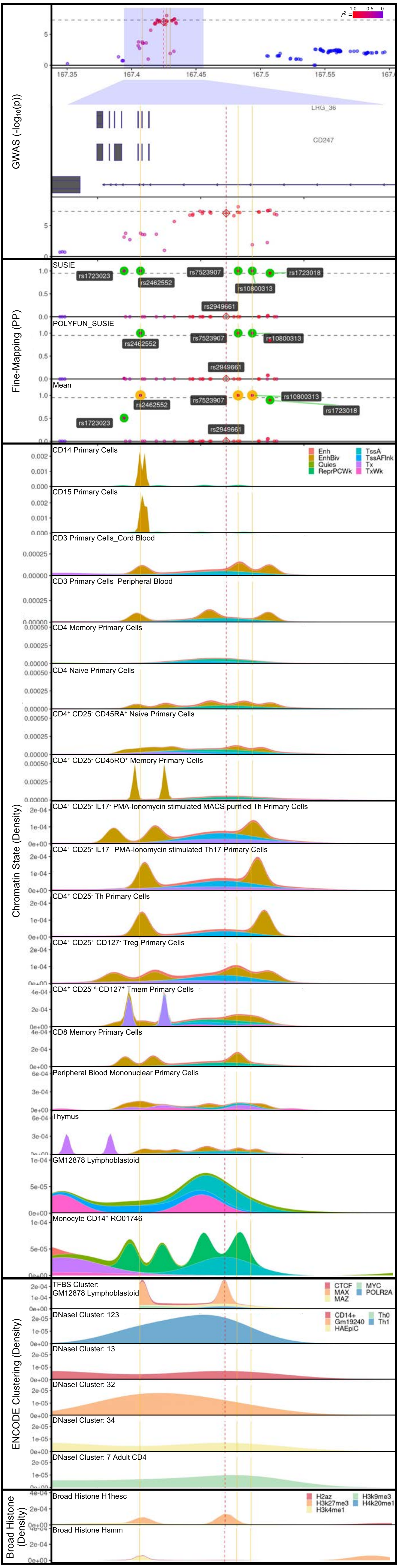

CD247_rs1214595

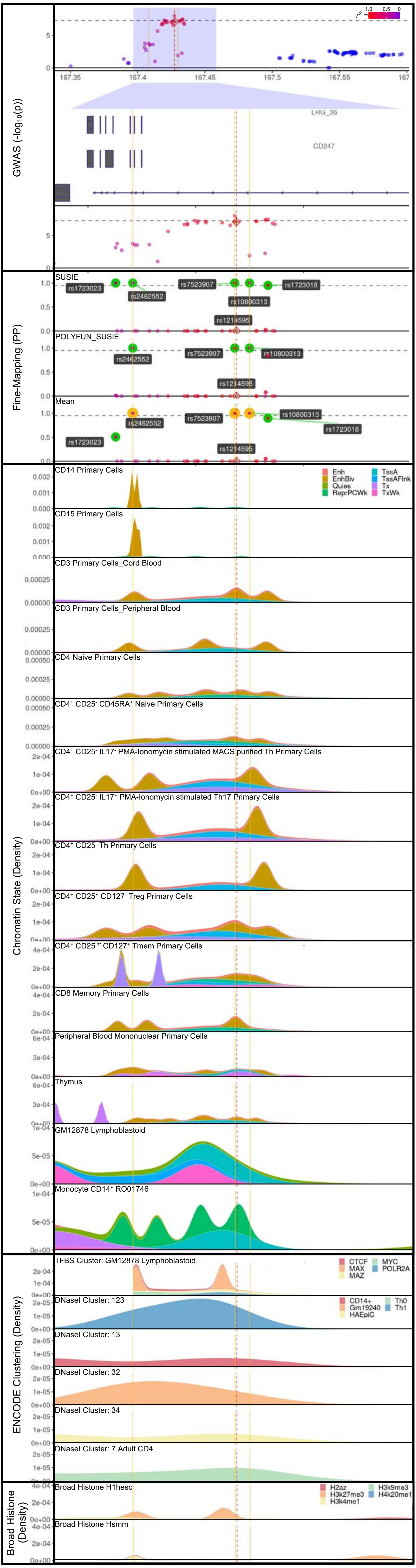

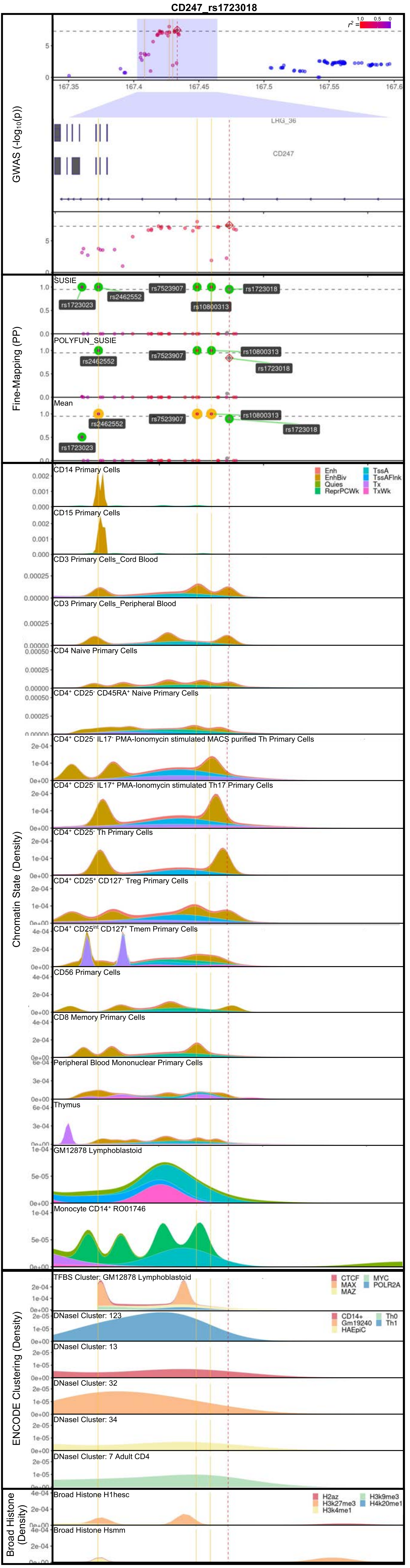
CD247_rs1723018
GWAS (-log10(p))
LRG_36
CD247
Fine-Mapping (PP)
SUSIE
POLYFUN_SUSIE
Mean
rs1723023
rs2462552
rs7523907
rs10800313
rs1723018
Chromatin State (Density)
CD14 Primary Cells
CD15 Primary Cells
CD3 Primary Cells_Cord Blood
CD3 Primary Cells_Peripheral Blood
CD4 Naive Primary Cells
CD4+ CD25- CD45RA+ Naive Primary Cells
CD4+ CD25- IL17- PMA-Ionomycin stimulated MACS purified Th Primary Cells
CD4+ CD25- IL17+ PMA-Ionomycin stimulated Th17 Primary Cells
CD4+ CD25- Th Primary Cells
CD4+ CD25+ CD127- Treg Primary Cells
CD4+ CD25int CD127+ Tmem Primary Cells
CD56 Primary Cells
CD8 Memory Primary Cells
Peripheral Blood Mononuclear Primary Cells
Thymus
GM12878 Lymphoblastoid
Monocyte CD14+ RO01746
Enh
EnhBiv
Quies
ReprPCWk
TssA
TssAFlnk
Tx
TxWk
ENCODE Clustering (Density)
TFBS Cluster: GM12878 Lymphoblastoid
CTCF
MAX
MAZ
MYC
POLR2A
DNaseI Cluster: 123
DNaseI Cluster: 13
DNaseI Cluster: 32
DNaseI Cluster: 34
DNaseI Cluster: 7 Adult CD4
CD14+
Gm19240
HAEpiC
Th0
Th1
Broad Histone (Density)
Broad Histone H1hesc
Broad Histone Hsmm
H2az
H3k27me3
H3k4me1
H3k9me3
H4k20me1

Supplementary Figure 5: Conditional analysis, posterior probability analysis, chromatin looping, and eQTL mapping in the *XKR6* locus.

(a) Conditional analysis adjusting for rs2409780 identified a residual effect for rs3885690 (top panel), while conditional analysis adjusting for rs4841466 identified residual effect for rs2618485 (bottom panel).

(b) Posterior probabilities distribution of variants in *XKR6* locus, identifying rs4841466 as the most probable SNP.

(c-e) The pairwise *D'* (**c**), r^2 (**d**), and haplotypes with their frequencies of the top and functional variants in the region are displayed (**e**).

(f) Circos plot mapping the zoom regional Manhattan plot of the imputed GWAS data for the *XKR6* association on Chromosome 8 (outer most layer). All SNPs with a $-\log_{10}$(p-value) <0.05 are shown in black, or colored based on r^2 (red: r^2>0.08; orange: r^2>0.06). The index SNP of the *XKR6* association, rs4841466, is indicated. The outer circle displays the chromosome coordinate of the *XKR6* risk locus highlighted in blue. Genes that are eQTLs or exhibit chromatin interaction by Hi-C in GM12878 Epstein-Barr virus (EBV)-transformed B lymphocytes are reported on the inner circles in green or orange font, respectively, and are shown as links colored green or orange, respectively, on the inner most layer.

(g-j) Sjögren-SNPs rs11250099 (**g**), rs4841465 (**h**), rs11250098 (**i**), and rs4314618 (**j**) are positioned in an intronic enhancer of *XKR6*. Coalescence of chromatin-chromatin interaction and eQTL indicate that the enhancer loops to the *XKR6* and *MTMR9* promoters to modulate expression. Moreover, eQTL data for these SNPs in minor salivary gland implicate *XKR6*, *RP1L1*, and *SLC35G5*. For details, see Supplementary Table 13.

(k-n) EcholocatoR was used to identify, fine-map and annotate the *XKR6* region after specifying the index SNPs (indicated by red dotted line): rs11250099 (**k**), rs4841465 (**l**), rs11250098 (**m**), rs4314618 (**n**). Additional SNPs with plausible function were identified and indicated by the dotted yellow lines

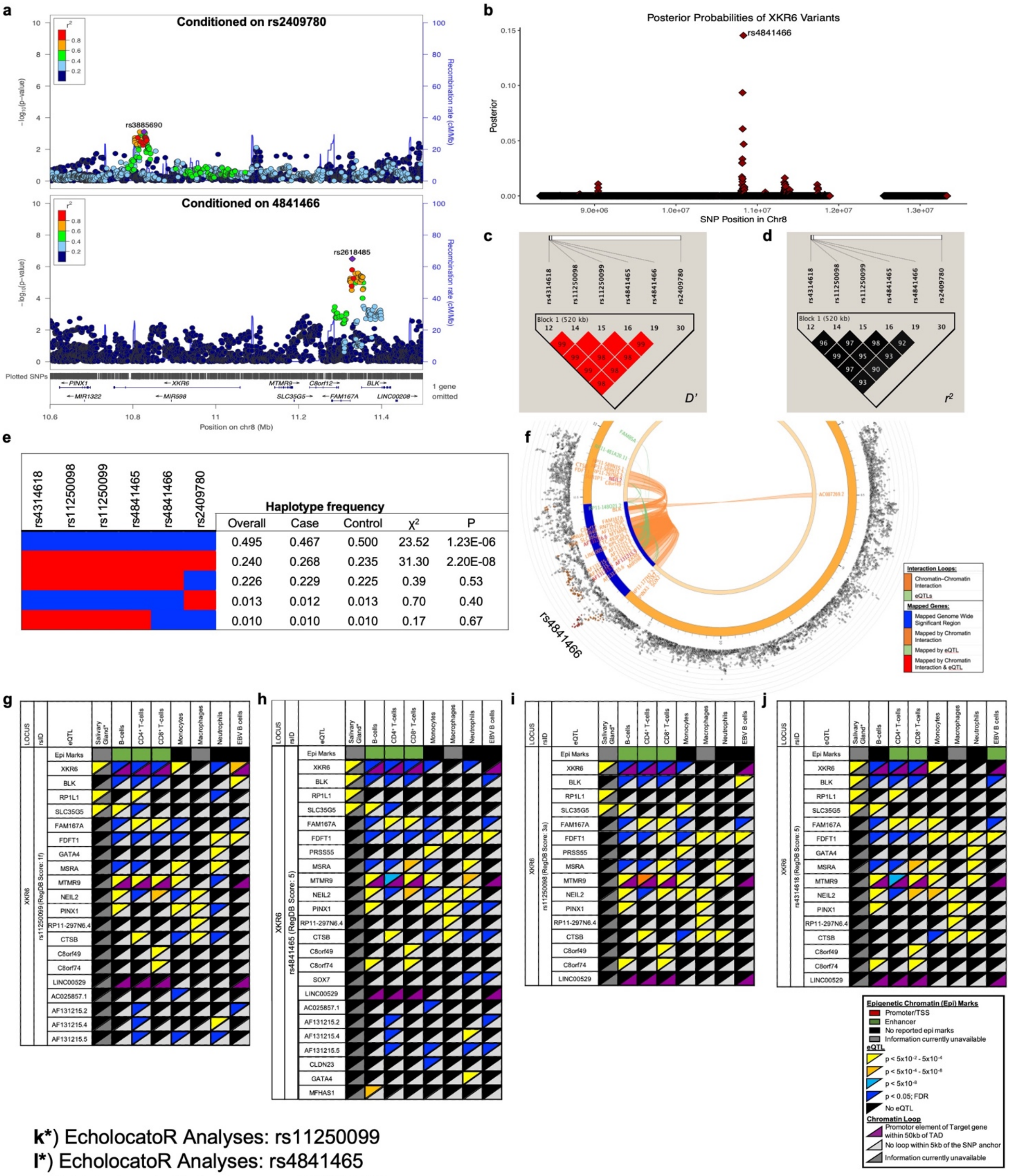

Haplotype frequency				
Overall	Case	Control	χ2	P
0.495	0.467	0.500	23.52	1.23E-06
0.240	0.268	0.235	31.30	2.20E-08
0.226	0.229	0.225	0.39	0.53
0.013	0.012	0.013	0.70	0.40
0.010	0.010	0.010	0.17	0.67

k*) EcholocatoR Analyses: rs11250099
l*) EcholocatoR Analyses: rs4841465
m*) EcholocatoR Analyses: rs11250098
n*) EcholocatoR Analyses: rs4314618
*see next page

XKR6_rs11250099

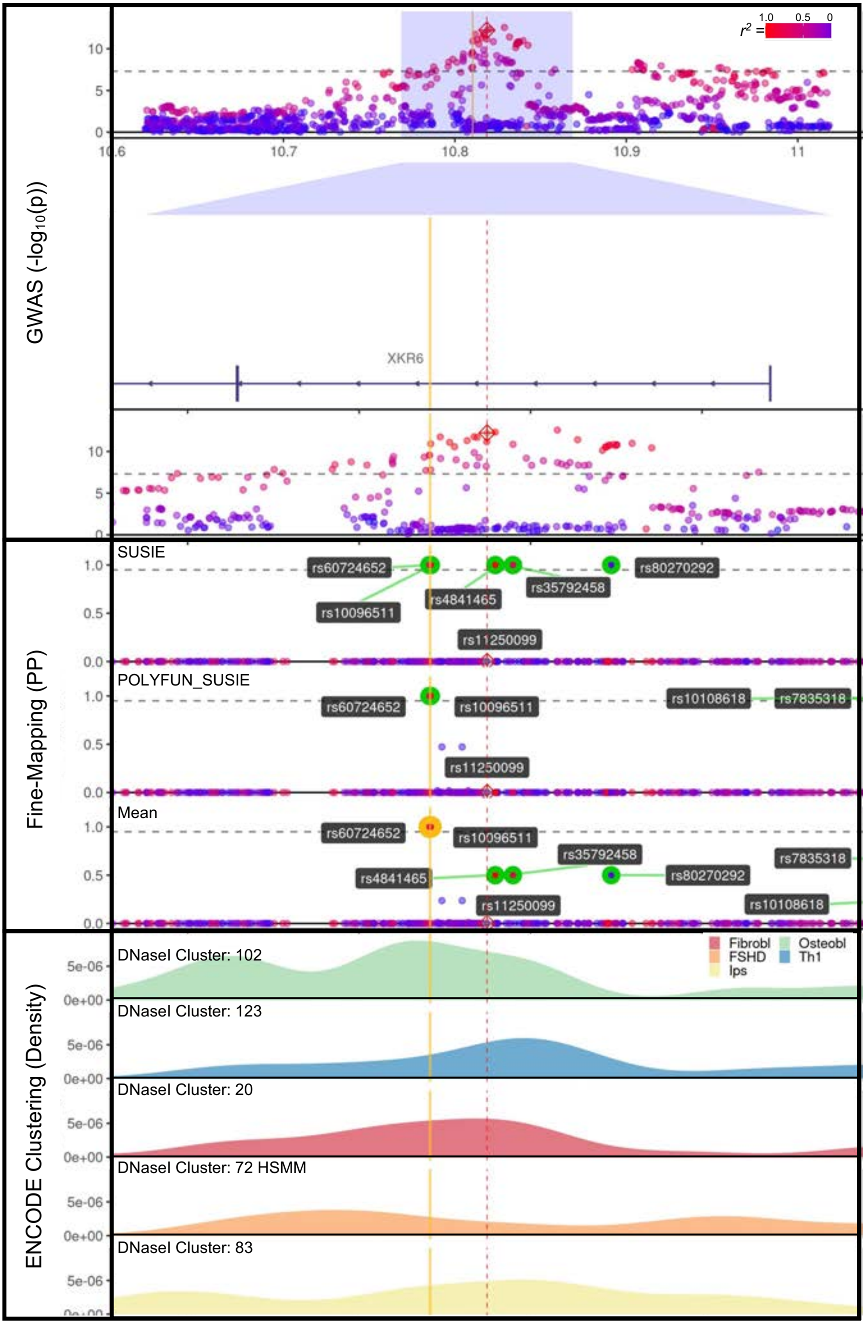

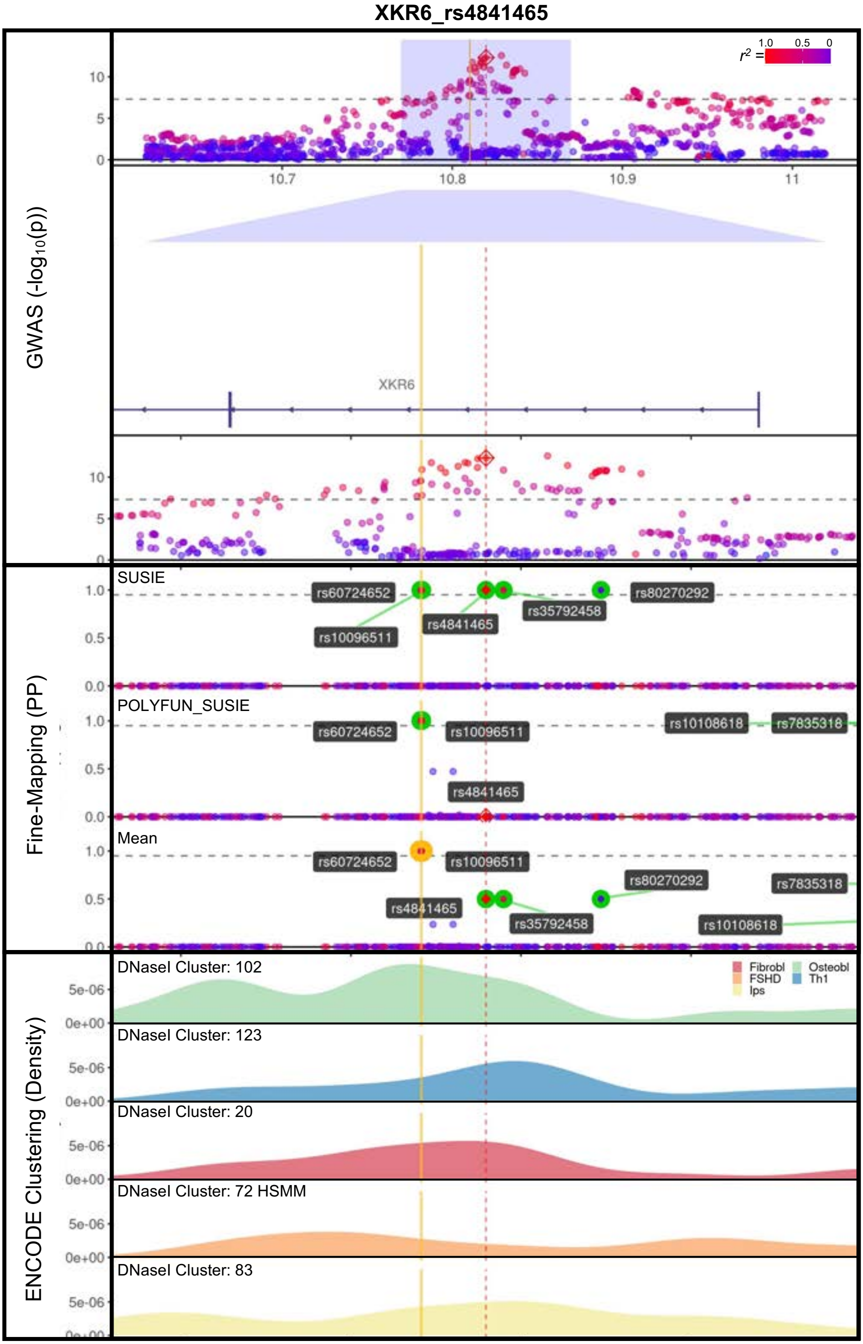
XKR6_rs4841465
GWAS (-log10(p))
r2 = 1.0 0.5 0
10.7
10.8
10.9
11
XKR6
Fine-Mapping (PP)
SUSIE
rs60724652
rs10096511
rs4841465
rs35792458
rs80270292
POLYFUN_SUSIE
rs10108618
rs7835318
Mean
ENCODE Clustering (Density)
DNaseI Cluster: 102
DNaseI Cluster: 123
DNaseI Cluster: 20
DNaseI Cluster: 72 HSMM
DNaseI Cluster: 83
Fibrobl
FSHD
Ips
Osteobl
Th1

XKR6_rs11250098

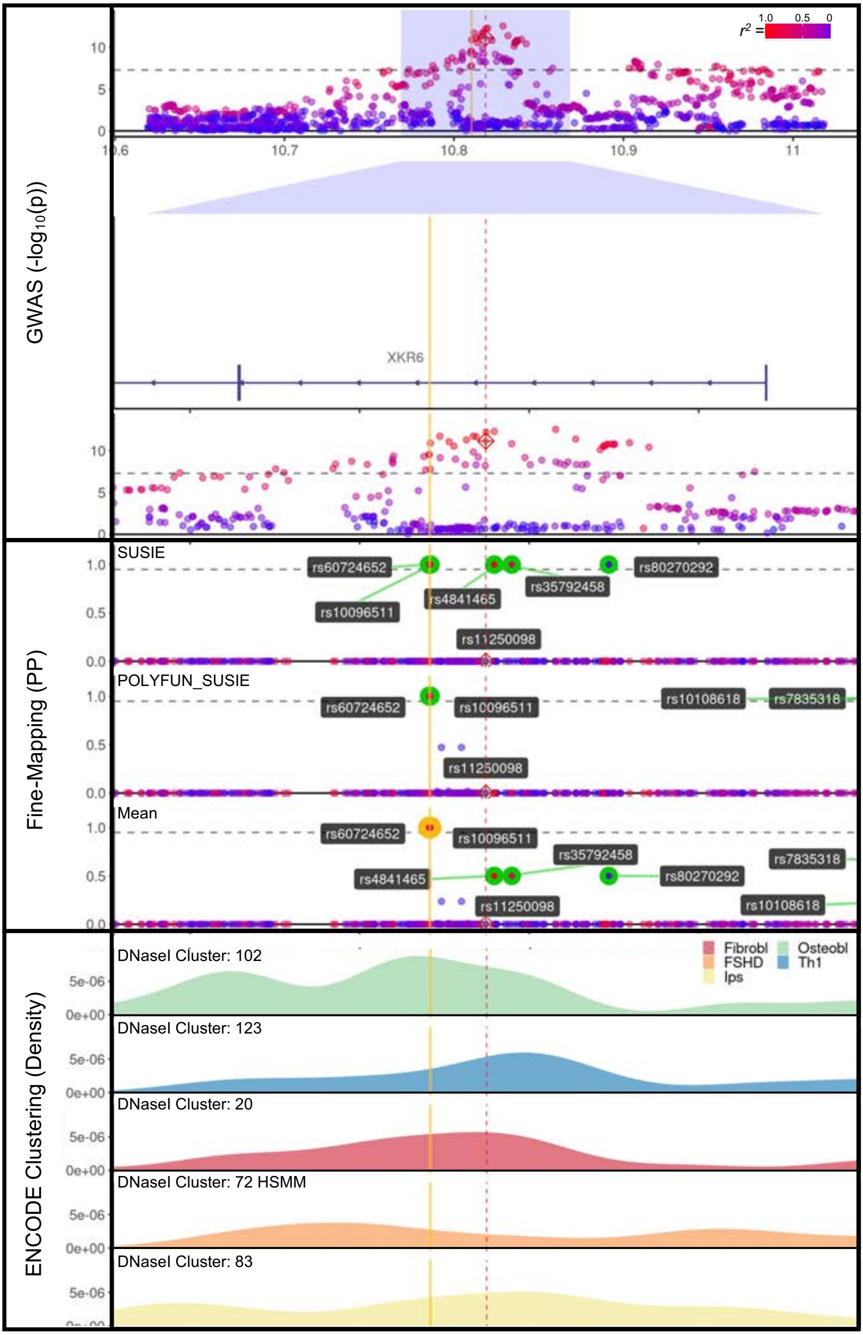

XKR6_rs4314618

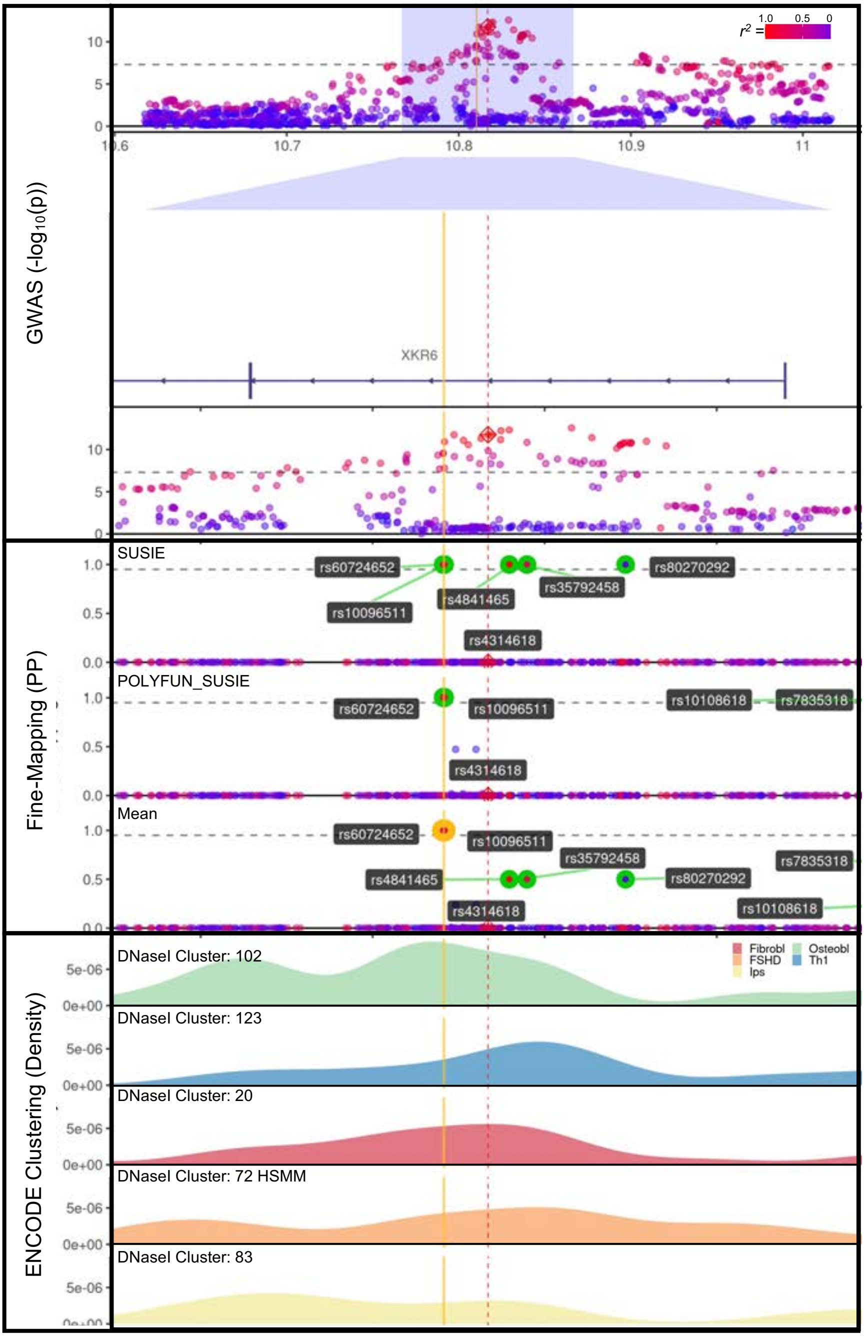

Supplementary Figure 6: Conditional analysis, posterior probability analysis, chromatin looping, and eQTL mapping in the *SYNGR1* locus.

(a) Conditional analysis was performed, adjusting for rs2069235. A residual effect was for the top SNP, rs77806631.

(b) Posterior probabilities distribution of variants in *SYNGR1* locus, identifying rs2069235 as the most probable SNP.

(c-e) The pairwise *D'* (**c**), r^2 (**d**), and haplotypes with their frequencies of the top and functional variants in the region are displayed (**e**).

(f) Circos plot mapping the zoom regional Manhattan plot of the imputed GWAS data for the *SYNGR1* association on Chromosome 22 (outer most layer). All SNPs with a $-\log_{10}$(p-value) <0.05 are shown in black, or colored based on r^2 (red: r^2>0.08; orange: r^2>0.06). The index SNP of the *SYNGR1* association, rs2069235, is indicated. The outer circle displays the chromosome coordinate of the *SYNGR1* risk locus highlighted in blue. Genes that are eQTLs or exhibit chromatin interaction by Hi-C in GM12878 Epstein-Barr virus (EBV)-transformed B lymphocytes are reported on the inner circles in green or orange font, respectively, and are shown as links colored green or orange, respectively, on the inner most layer.

(g-j) Sjögren-SNPs rs2069235 (**g**), rs909685 (**h**), rs2267407 (**i**), and rs3747177 (**j**) are positioned in an intronic regulatory element of *SYNGR1*. Epigenetic enrichment of enhancer and promoter marks, and the coalescence of chromatin-chromatin interactions and eQTL data indicate that this element likely functions as both a promoter to modulate the expression of an alternative *SYNGR1* isoform and/or an enhancer that regulates the promoter of *ATF4* and *CBX7*. For details, see Supplementary Table 16.

(k-m) Sjögren-SNPs rs5757585 (**k**), rs11703434 (**l**), and rs137594 (**m**) are positioned in an intergenic region upstream of *SYNGR1*. Epigenetic marks and eQTL enrichment indicate that both variants modulate enhancer activity in specific immune cell types, predominantly monocytes. For details, see Supplementary Table 16.

(n-t) EcholocatoR was used to identify, fine-map and annotate the *SYNGR1* region after specifying the index SNPs (indicated by red dotted line): rs2069235 (**n**), rs909685 (**o**), rs2267407 (**p**), rs3747177 (**q**), rs5757585 (**r**), rs11703434 (**s**), rs137594 (**t**). Additional SNPs with plausible function were identified and indicated by the dotted yellow lines.

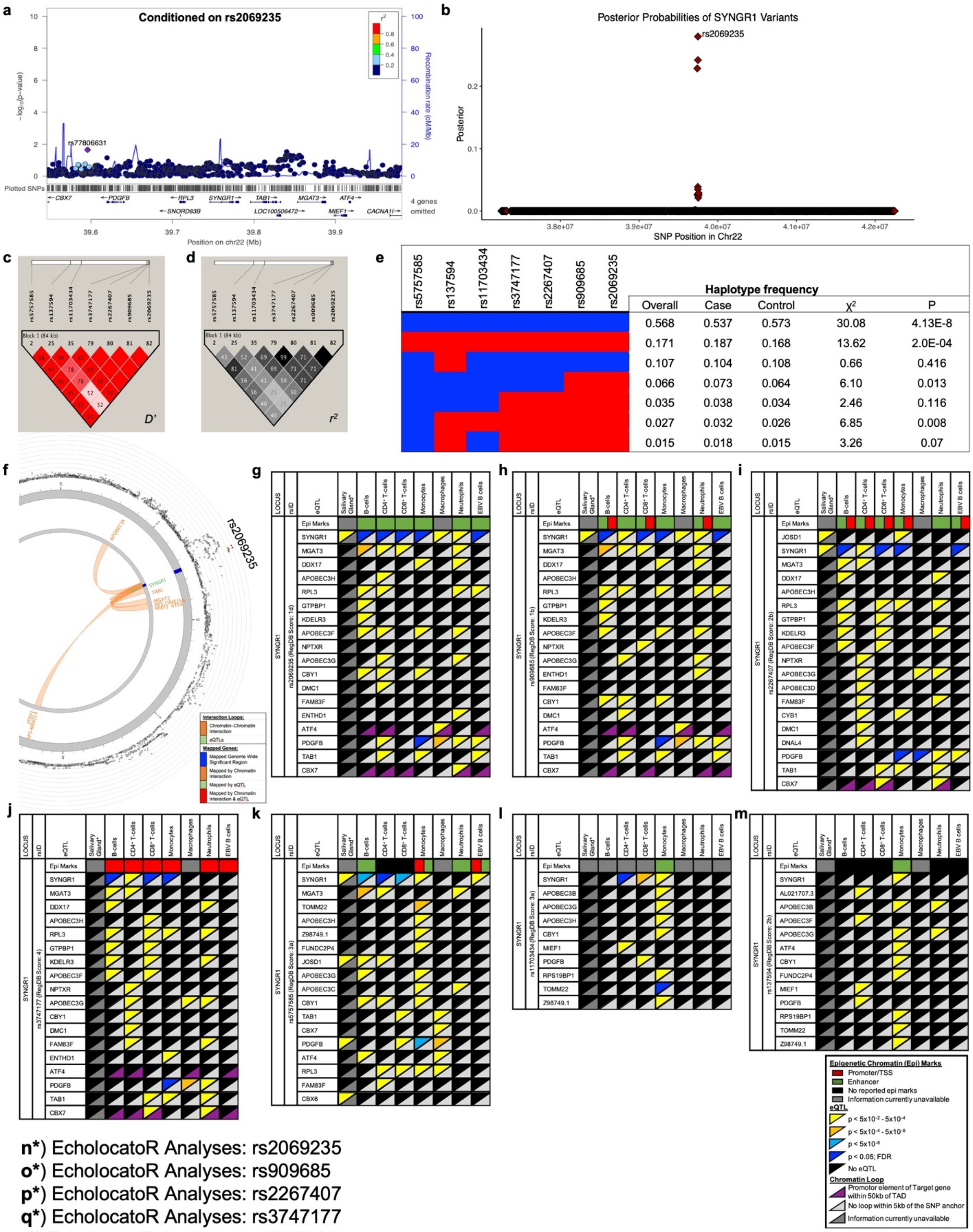

n*) EcholocatoR Analyses: rs2069235

o*) EcholocatoR Analyses: rs909685

p*) EcholocatoR Analyses: rs2267407

q*) EcholocatoR Analyses: rs3747177

r*) EcholocatoR Analyses: rs5757585

s*) EcholocatoR Analyses: rs11703434

t*) EcholocatoR Analyses: rs137594

*see next page

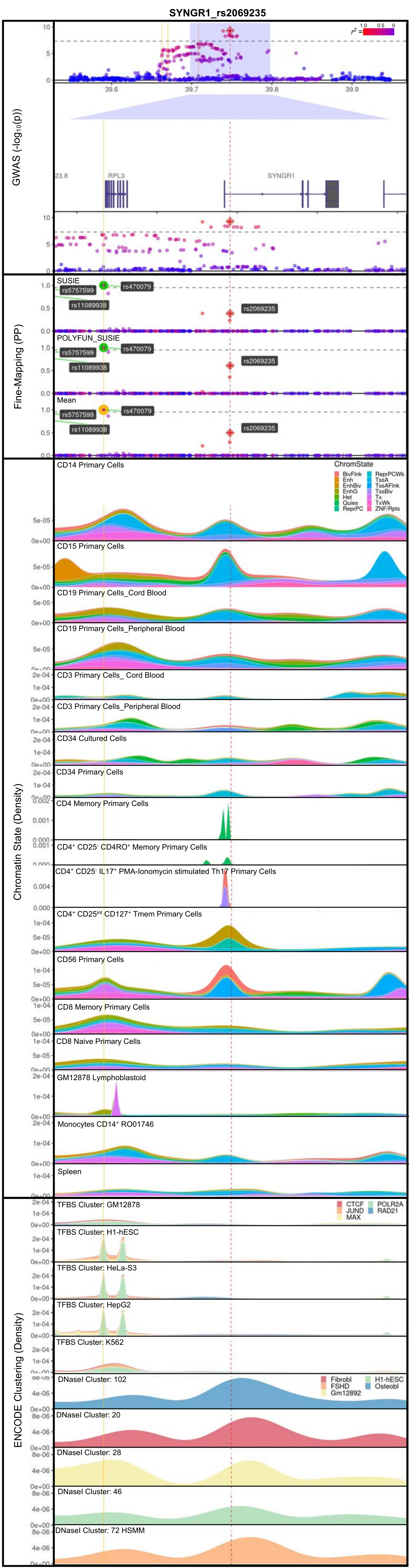
SYNGR1_rs2069235
GWAS (-log10(p))
Fine-Mapping (PP)
SUSIE
POLYFUN_SUSIE
Mean
rs5757599
rs470079
rs11089938
rs2069235
RPL3
SYNGR1
Chromatin State (Density)
ChromState
BivFlnk
Enh
EnhBiv
EnhG
Het
Quies
ReprPC
ReprPCWk
TssA
TssAFlnk
TssBiv
Tx
TxWk
ZNF/Rpts
CD14 Primary Cells
CD15 Primary Cells
CD19 Primary Cells_Cord Blood
CD19 Primary Cells_Peripheral Blood
CD3 Primary Cells_ Cord Blood
CD3 Primary Cells_Peripheral Blood
CD34 Cultured Cells
CD34 Primary Cells
CD4 Memory Primary Cells
CD4+ CD25- CD4RO+ Memory Primary Cells
CD4+ CD25- IL17+ PMA-Ionomycin stimulated Th17 Primary Cells
CD4+ CD25int CD127+ Tmem Primary Cells
CD56 Primary Cells
CD8 Memory Primary Cells
CD8 Naive Primary Cells
GM12878 Lymphoblastoid
Monocytes CD14+ RO01746
Spleen
ENCODE Clustering (Density)
TFBS Cluster: GM12878
CTCF
JUND
MAX
POLR2A
RAD21
TFBS Cluster: H1-hESC
TFBS Cluster: HeLa-S3
TFBS Cluster: HepG2
TFBS Cluster: K562
DNaseI Cluster: 102
Fibrobl
FSHD
Gm12892
H1-hESC
Osteobl
DNaseI Cluster: 20
DNaseI Cluster: 28
DNaseI Cluster: 46
DNaseI Cluster: 72 HSMM

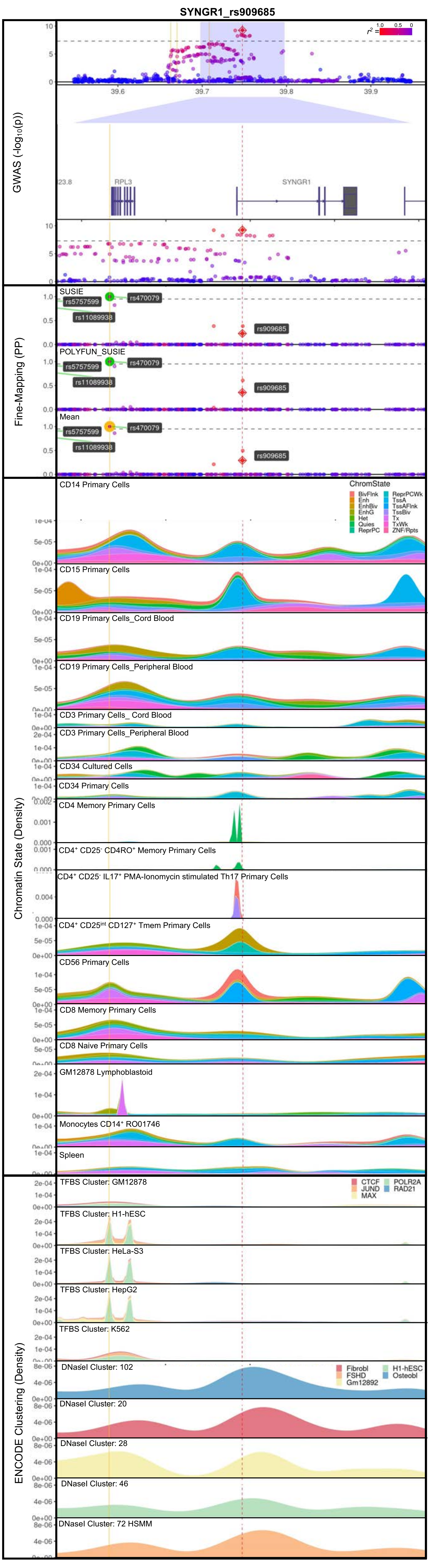

SYNGR1_rs909685
GWAS (-log10(p))
Fine-Mapping (PP)
SUSIE
POLYFUN_SUSIE
Mean
rs5757599
rs470079
rs11089938
rs909685
RPL3
SYNGR1
Chromatin State (Density)
ChromState
BivFlnk
Enh
EnhBiv
EnhG
Het
Quies
ReprPC
ReprPCWk
TssA
TssAFlnk
TssBiv
Tx
TxWk
ZNF/Rpts
CD14 Primary Cells
CD15 Primary Cells
CD19 Primary Cells_Cord Blood
CD19 Primary Cells_Peripheral Blood
CD3 Primary Cells_ Cord Blood
CD3 Primary Cells_Peripheral Blood
CD34 Cultured Cells
CD34 Primary Cells
CD4 Memory Primary Cells
CD4+ CD25- CD4RO+ Memory Primary Cells
CD4+ CD25- IL17+ PMA-Ionomycin stimulated Th17 Primary Cells
CD4+ CD25int CD127+ Tmem Primary Cells
CD56 Primary Cells
CD8 Memory Primary Cells
CD8 Naive Primary Cells
GM12878 Lymphoblastoid
Monocytes CD14+ RO01746
Spleen
ENCODE Clustering (Density)
TFBS Cluster: GM12878
CTCF
JUND
MAX
POLR2A
RAD21
TFBS Cluster: H1-hESC
TFBS Cluster: HeLa-S3
TFBS Cluster: HepG2
TFBS Cluster: K562
DNaseI Cluster: 102
Fibrobl
FSHD
Gm12892
H1-hESC
Osteobl
DNaseI Cluster: 20
DNaseI Cluster: 28
DNaseI Cluster: 46
DNaseI Cluster: 72 HSMM

SYNGR1_rs2267407

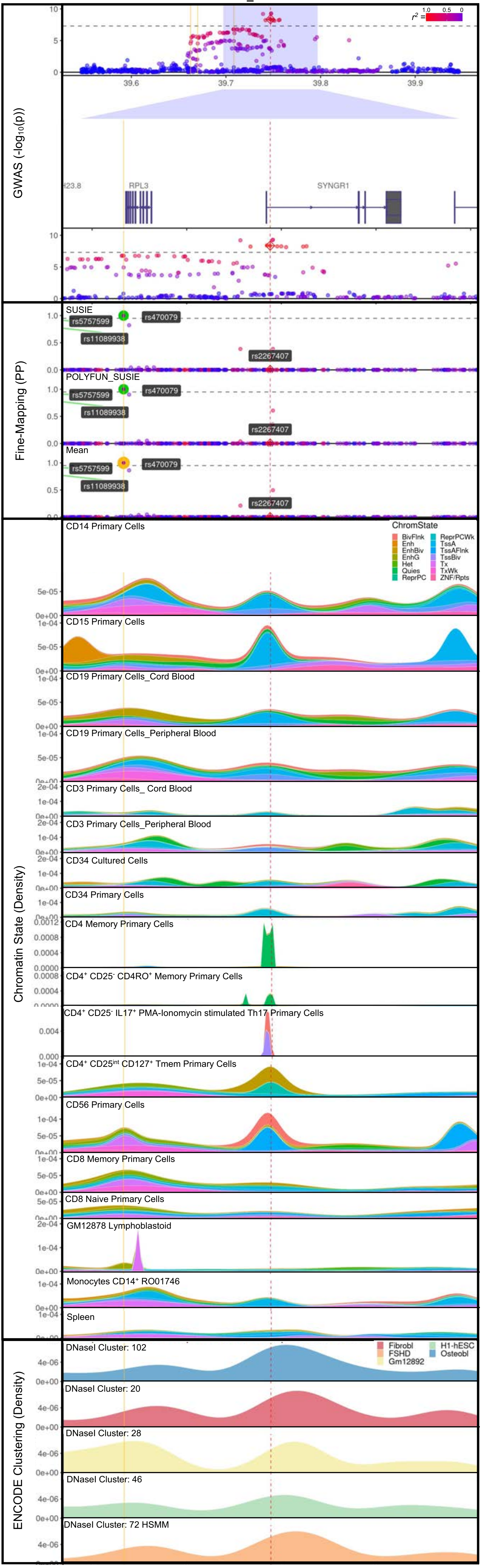

SYNGR1_rs3747177

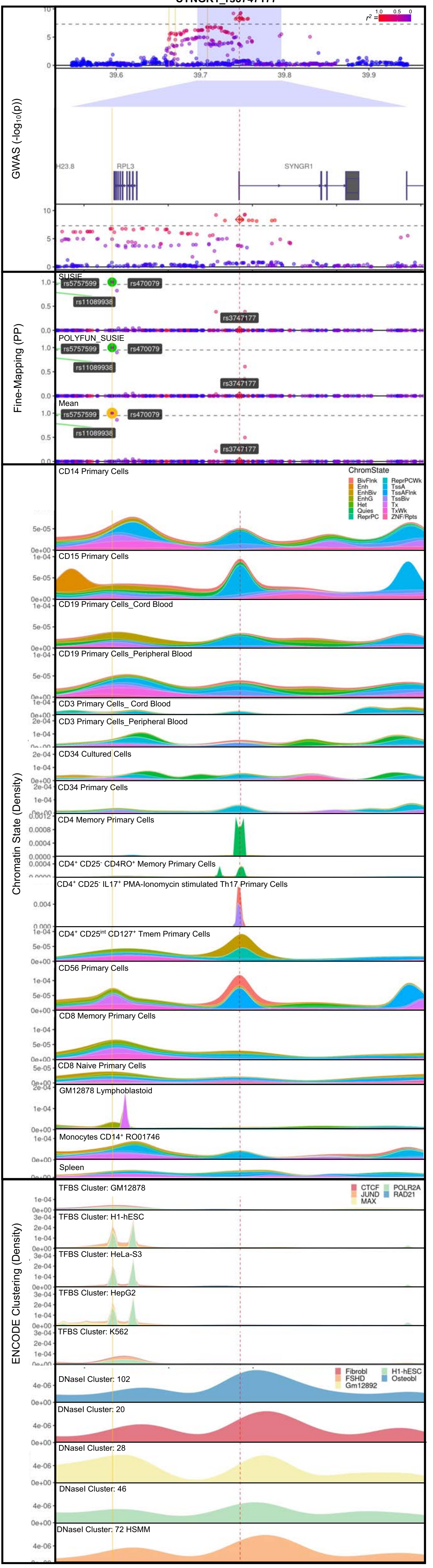

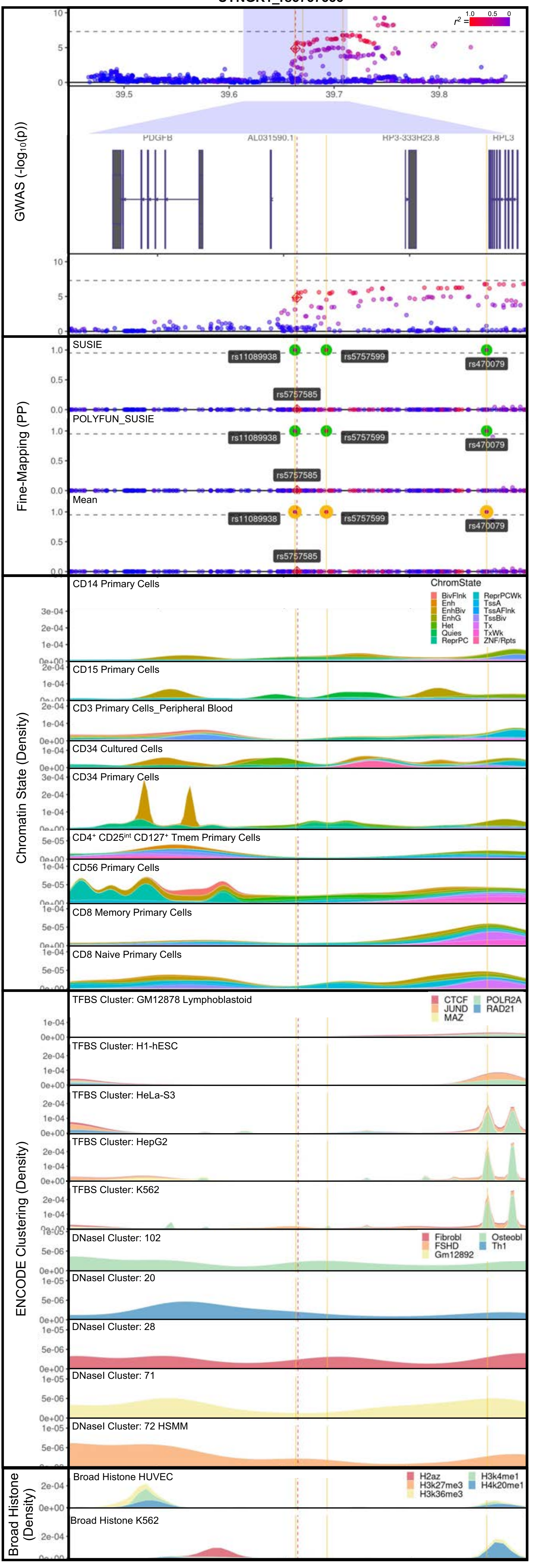
SYNGR1_rs5757585
GWAS (-log10(p))
Fine-Mapping (PP)
Chromatin State (Density)
ENCODE Clustering (Density)
Broad Histone (Density)
PDGFB
AL031590.1
RP3-333H23.8
RPL3
SUSIE
POLYFUN_SUSIE
Mean
rs11089938
rs5757599
rs470079
rs5757585
CD14 Primary Cells
CD15 Primary Cells
CD3 Primary Cells_Peripheral Blood
CD34 Cultured Cells
CD34 Primary Cells
CD4+ CD25int CD127+ Tmem Primary Cells
CD56 Primary Cells
CD8 Memory Primary Cells
CD8 Naive Primary Cells
ChromState
BivFlnk
Enh
EnhBiv
EnhG
Het
Quies
ReprPC
ReprPCWk
TssA
TssAFlnk
TssBiv
Tx
TxWk
ZNF/Rpts
TFBS Cluster: GM12878 Lymphoblastoid
TFBS Cluster: H1-hESC
TFBS Cluster: HeLa-S3
TFBS Cluster: HepG2
TFBS Cluster: K562
CTCF
JUND
MAZ
POLR2A
RAD21
DNaseI Cluster: 102
DNaseI Cluster: 20
DNaseI Cluster: 28
DNaseI Cluster: 71
DNaseI Cluster: 72 HSMM
Fibrobl
FSHD
Gm12892
Osteobl
Th1
Broad Histone HUVEC
Broad Histone K562
H2az
H3k27me3
H3k36me3
H3k4me1
H4k20me1

SYNGR1_rs11703434

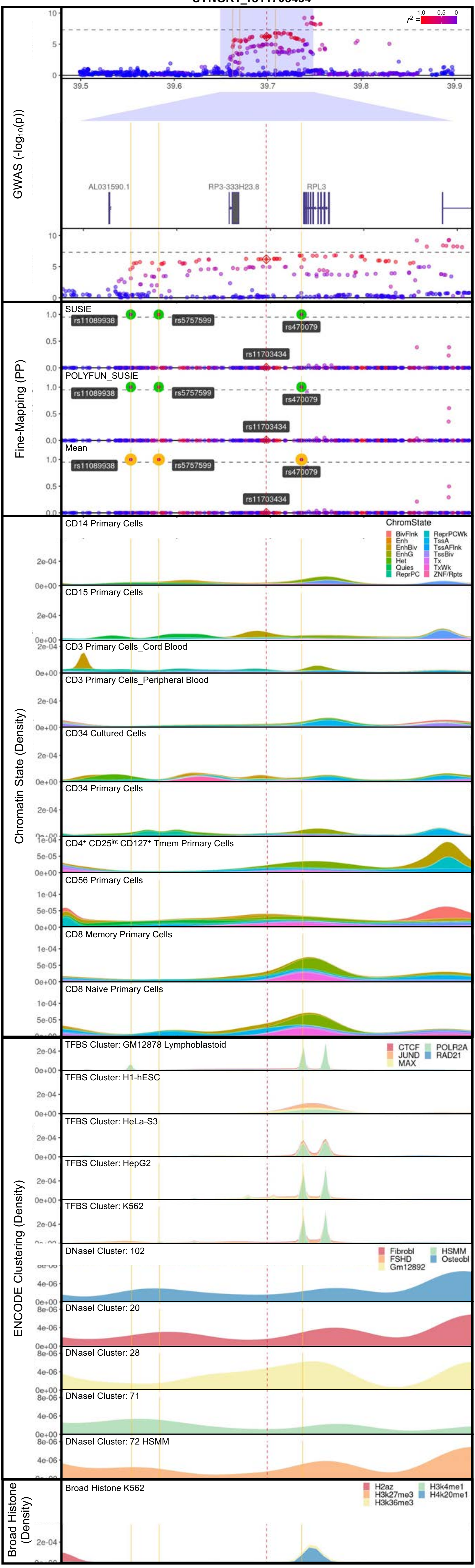

SYNGR1_rs137594

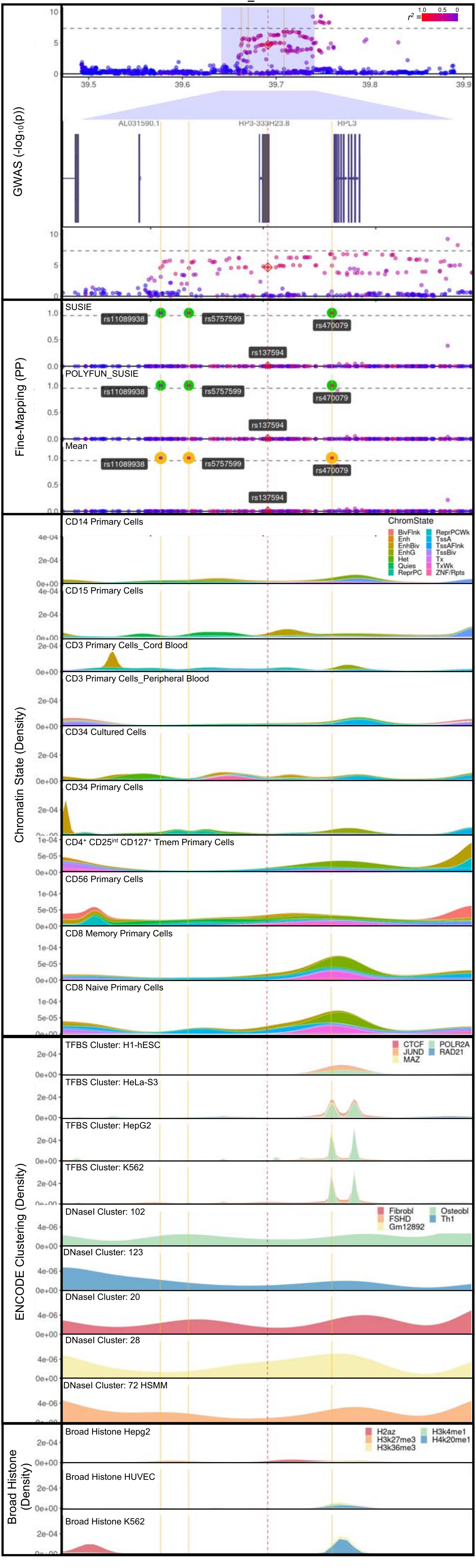

Supplementary Figure 7: Conditional analysis, posterior probability analysis, chromatin looping, and eQTL mapping in the *NAB1* locus.

(a) Conditional analysis adjusting for rs4274624 identified a residual effect for rs4853457 (top panel), while conditional analysis adjusting for rs4274624 and rs4853457 identified a residual effect for rs2293765 (middle panel). Conditional analysis adjusting for rs2293765 identified a residual effect for rs4274624 (bottom panel).

(b) Posterior probabilities distribution of variants in *NAB1* locus, identifying rs2293765 as the most probable SNP.

(c-e) The pairwise *D*' (**c**), r^2 (**d**), and haplotypes with their frequencies of the top and functional variants in the region are displayed (**e**).

(f) Circos plot mapping the zoom regional Manhattan plot of the imputed GWAS data for the *NAB1* association on Chromosome 2 (outer most layer). All SNPs with a $-\log_{10}$(p-value) <0.05 are shown in black, or colored based on r^2 (red: r^2>0.08; orange: r^2>0.06). The index SNP of the *NAB1* association, rs4274624, is indicated. The outer circle displays the chromosome coordinate of the *NAB1* risk locus highlighted in blue. Genes that are eQTLs or exhibit chromatin interaction by Hi-C in GM12878 Epstein-Barr virus (EBV)-transformed B lymphocytes are reported on the inner circles in green or orange font, respectively, and are shown as links colored green or orange, respectively, on the inner most layer.

(g-h) Sjögren-SNPs rs2293765 (**g**) and rs2192008 (**h**), positioned in intronic enhancer elements of *NAB1*, exhibited epigenetic enhancer and promoter marks in several immune cell types (rectangles). Coalescence of chromatin-chromatin interactions (purple triangles) and several common eQTLs (yellow, orange, blue triangles), suggest that the intronic enhancer regulates the promoters of *MFSD6* (Major Facilitator Superfamily Domain Containing 6 likely regulates nutrient uptake across cell membranes) and *TMEM194B/NEMP2* (Nuclear Envelope Integral Membrane Protein 2 has unknown function). For details, see Supplementary Table 19.

(i) Sjögren-SNP rs11900804, positioned in an intronic enhancer element of *NAB1*, has limited epigenetic enhancer and promoter activity in $CD4^+$ T cells, and a coalescence of chromatin-chromatin interaction data and eQTLs for *MFSD6* and *TMEM194B/NEMP2*.

(j) Lack of epigenetic marks suggest that Sjögren-SNP rs744600 is likely not function, despite being a SNP from the 95% credible set for the *NAB1* region. For details, see Supplementary Table 19.

(k-n) EcholocatoR was used to identify, fine-map and annotate the *NAB1* region after specifying the index SNPs (indicated by red dotted line): rs2293765 (**k**), rs2192008 (**l**), rs11900804 (**m**), rs744600 (**n**). Additional SNPs with plausible function were identified and indicated by the dotted yellow lines.

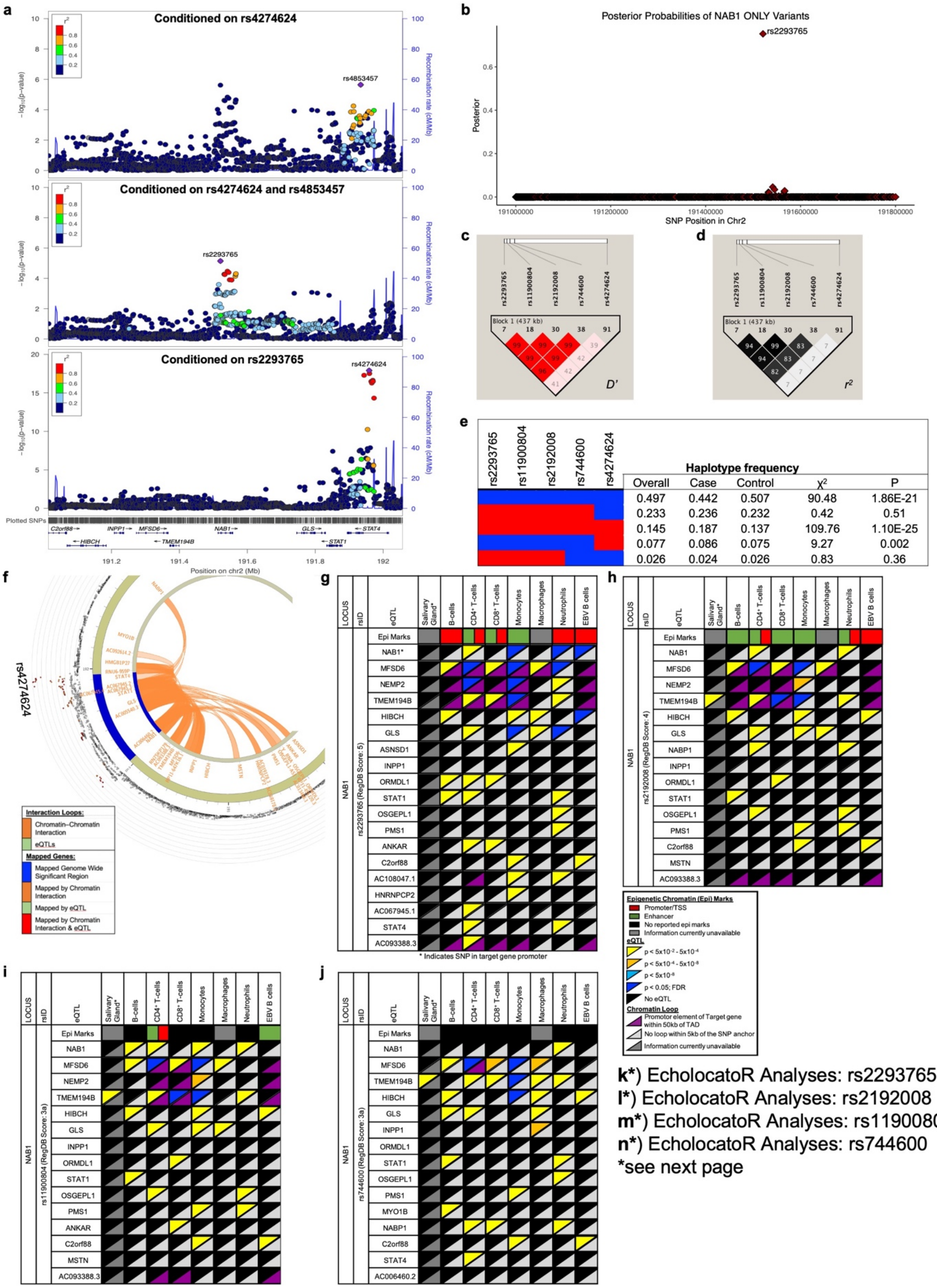

k*) EcholocatoR Analyses: rs2293765
l*) EcholocatoR Analyses: rs2192008
m*) EcholocatoR Analyses: rs11900804
n*) EcholocatoR Analyses: rs744600
*see next page

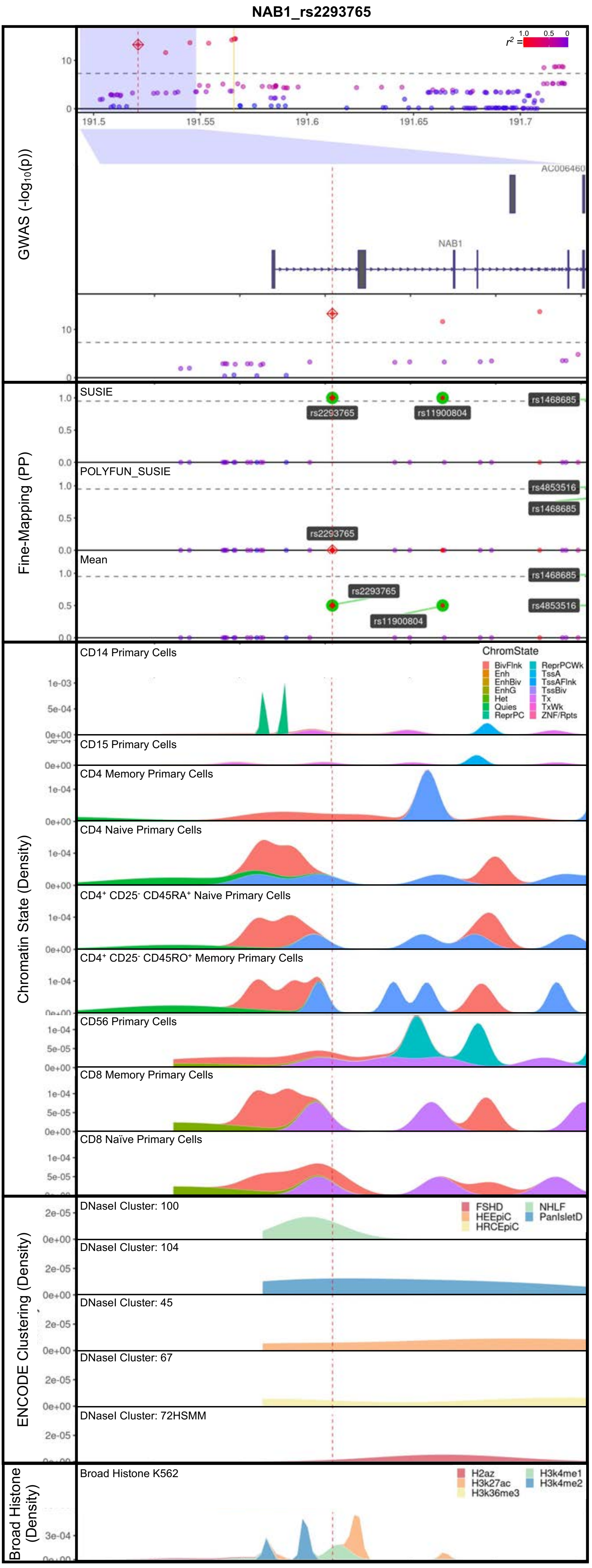

NAB1_rs2293765
GWAS (-log10(p))
Fine-Mapping (PP)
Chromatin State (Density)
ENCODE Clustering (Density)
Broad Histone (Density)
SUSIE
POLYFUN_SUSIE
Mean
rs2293765
rs11900804
rs1468685
rs4853516
NAB1
AC006460
CD14 Primary Cells
CD15 Primary Cells
CD4 Memory Primary Cells
CD4 Naive Primary Cells
CD4+ CD25- CD45RA+ Naive Primary Cells
CD4+ CD25- CD45RO+ Memory Primary Cells
CD56 Primary Cells
CD8 Memory Primary Cells
CD8 Naïve Primary Cells
ChromState
BivFlnk
Enh
EnhBiv
EnhG
Het
Quies
ReprPC
ReprPCWk
TssA
TssAFlnk
TssBiv
Tx
TxWk
ZNF/Rpts
DNaseI Cluster: 100
DNaseI Cluster: 104
DNaseI Cluster: 45
DNaseI Cluster: 67
DNaseI Cluster: 72HSMM
FSHD
HEEpiC
HRCEpiC
NHLF
PanIsletD
Broad Histone K562
H2az
H3k27ac
H3k36me3
H3k4me1
H3k4me2

NAB1_rs2192008

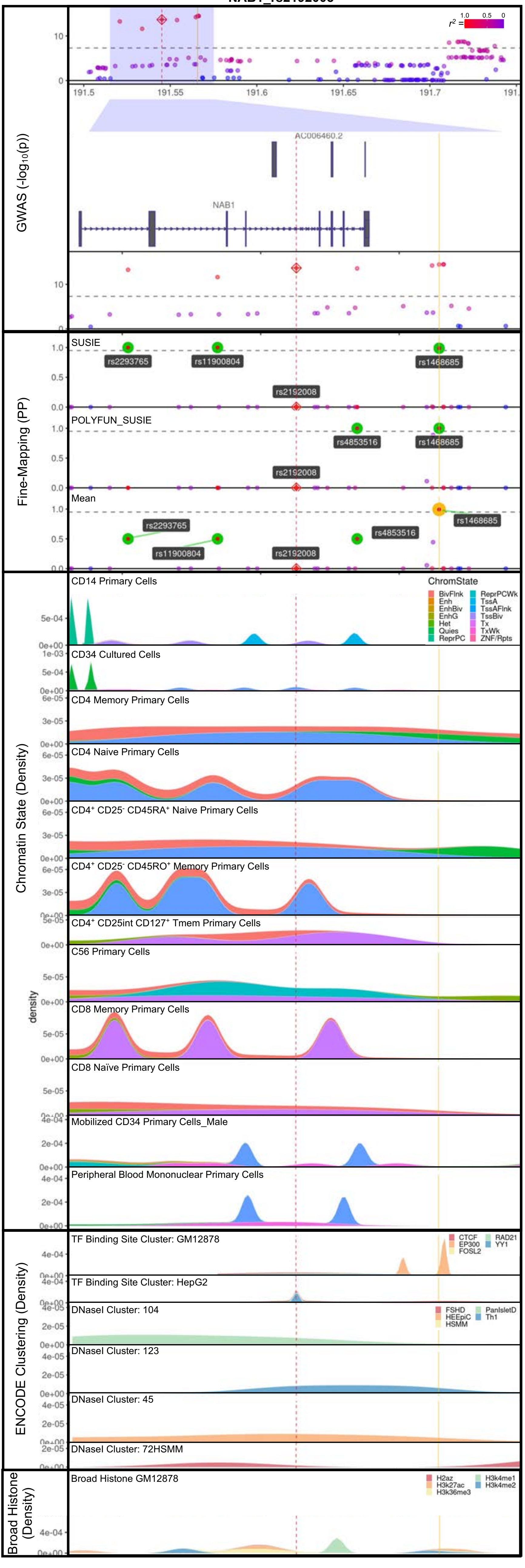

NAB1_rs11900804

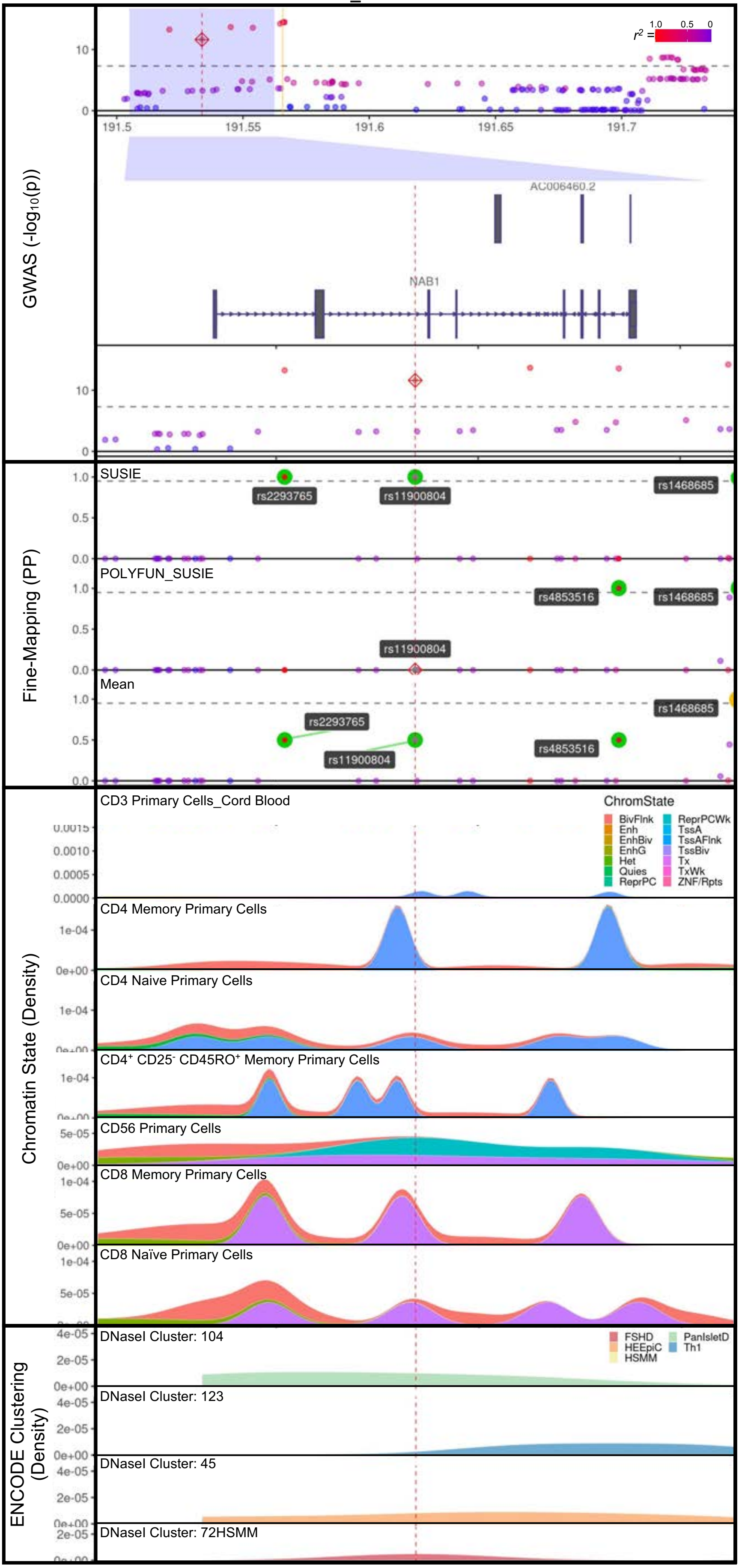

NAB1_rs744600

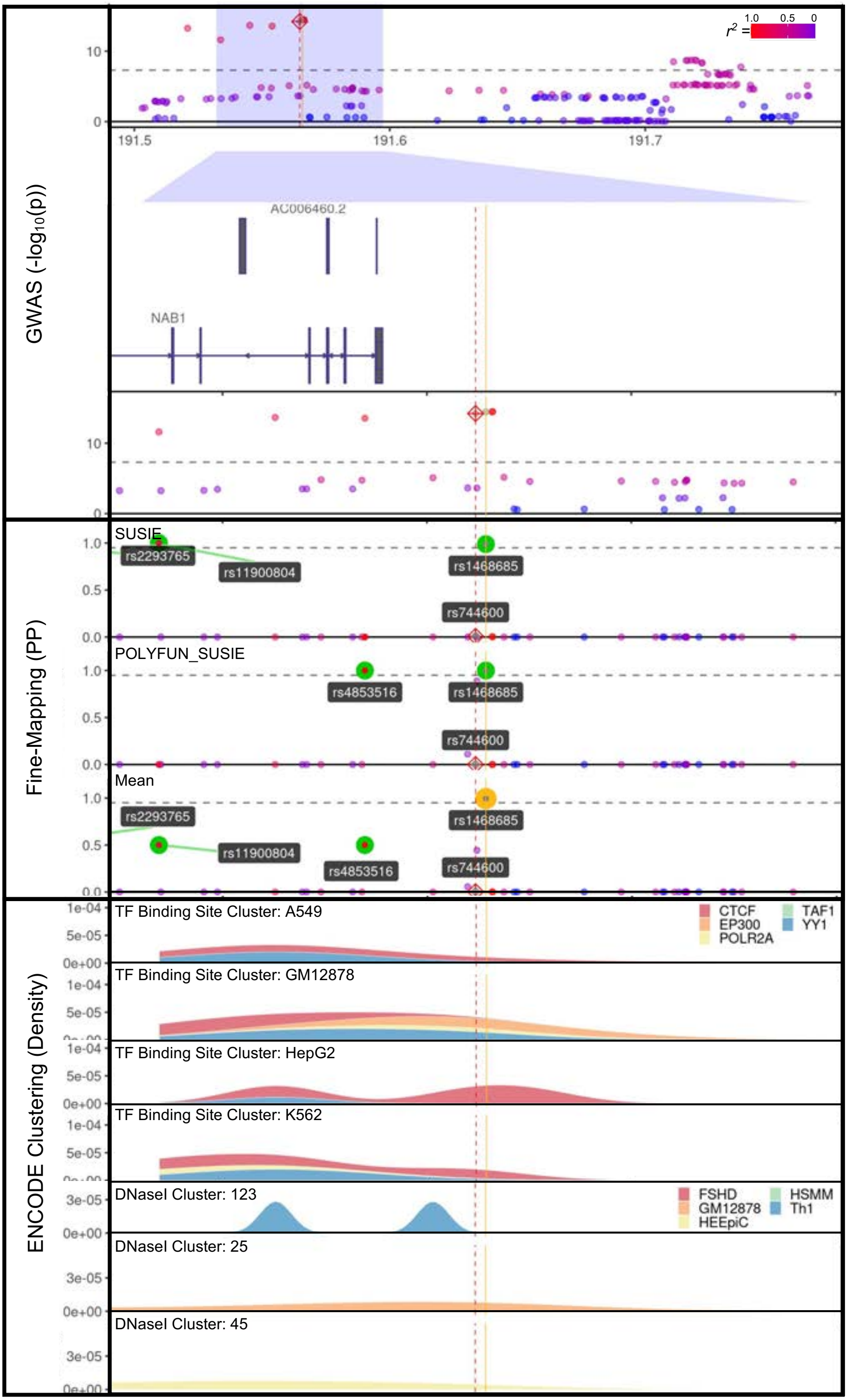

Supplementary Figure 8: Conditional analysis, posterior probability analysis, chromatin looping, and eQTL mapping in the *RPTOR-CHMP6-BAIAP6* locus.

(a) Conditional analysis was performed, adjusting for rs8071514. A residual effect was for the top SNP, rs139722484.

(b) Posterior probabilities distribution of variants in *RPTOR-CHMP6-BAIAP6* locus, identifying rs8071514 as the most probable SNP.

(c-e) The pairwise *D'* (**c**), r^2 (**d**), and haplotypes with their frequencies of the top and functional variants in the region are displayed (**e**).

(f) Circos plot mapping the zoom regional Manhattan plot of the imputed GWAS data for the *RPTOR-CHMP6-BAIAP6* association on Chromosome 17 (outer most layer). All SNPs with a $-\log_{10}$(p-value) <0.05 are shown in black, or colored based on r^2 (red: r^2>0.08; orange: r^2>0.06). The index SNP of the *RPTOR-CHMP6-BAIAP6* association, rs8071514, is indicated. The outer circle displays the chromosome coordinate of the *RPTOR-CHMP6-BAIAP6* risk locus highlighted in blue. Genes that are eQTLs or exhibit chromatin interaction by Hi-C in GM12878 Epstein-Barr virus (EBV)-transformed B lymphocytes are reported on the inner circles in green or orange font, respectively, and are shown as links colored green or orange, respectively, on the inner most layer.

(g, h) Sjögren-SNP rs6565516 (**g**) and rs4969328 (**h**) are positioned in the *CHMP6* promoter region, have epigenetic marks consistent with promoter activity and are reported *CHMP6* eQTLs. *CHMP6* encodes the charged multivesicular body protein 6, an important component of the ESCRT-III complex involved in endosomal sorting for degradation. Additional epigenetic enhancer marks, chromatin-chromatin interactions, and eQTL data suggest that these variants may also modulate enhancer that engages the *RPTOR* promoter. *RPTOR* (Regulatory Associated Protein of MTOR Complex 1) is an important regulator of nutrient sensing and autophagy during nutrient deprivation. The two SNPs are also minor salivary gland eQTLs for *TMEM105* and *FAM165B* ~314 kb and ~816 kb downstream downstream. For details, see Supplementary Table 22.

(i, j) Sjögren-SNP rs4969331 (**i**) and rs6565518 (**j**) are positioned in an intronic element with epigenetic evidence of enhancer and promoter activity. Chromatin-chromatin interaction data indicates potential looping to an upstream region enriched with non-protein-coding genes, however reporting of ncRNAs in eQTL databases is limited, preventing detecting of any coalescence between eQTL and chromatin-chromatin interaction at this region. For details, see Supplementary Table 22.

(k-n) EcholocatoR was used to identify, fine-map and annotate the *RPTOR-CHMP6-BAIAP6* region after specifying the index SNPs (indicated by red dotted line): rs6565516 (**k**), rs4969328 (**l**), rs4969331 (**m**), and rs6565518 (**n**). Additional SNPs with plausible function were identified and indicated by the dotted yellow lines.

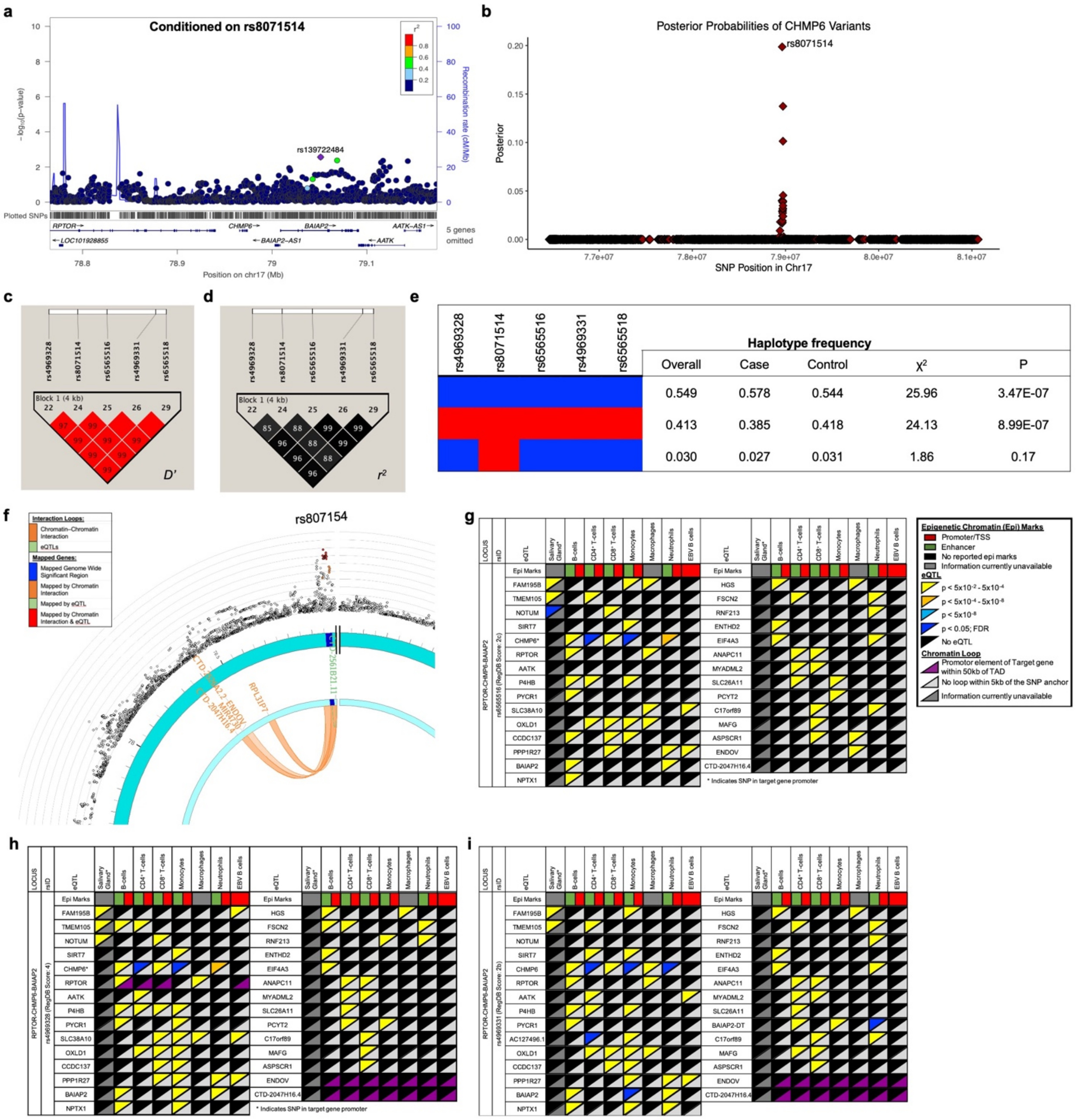

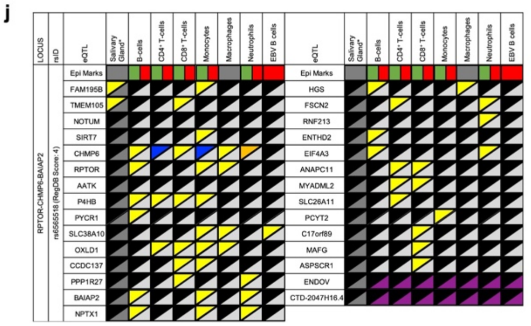

k*) EcholocatoR Analyses: rs6565516

l*) EcholocatoR Analyses: rs4969328

m*) EcholocatoR Analyses: rs4969331

n*) EcholocatoR Analyses: rs6565518

*see next page

CHMP6_rs6565516

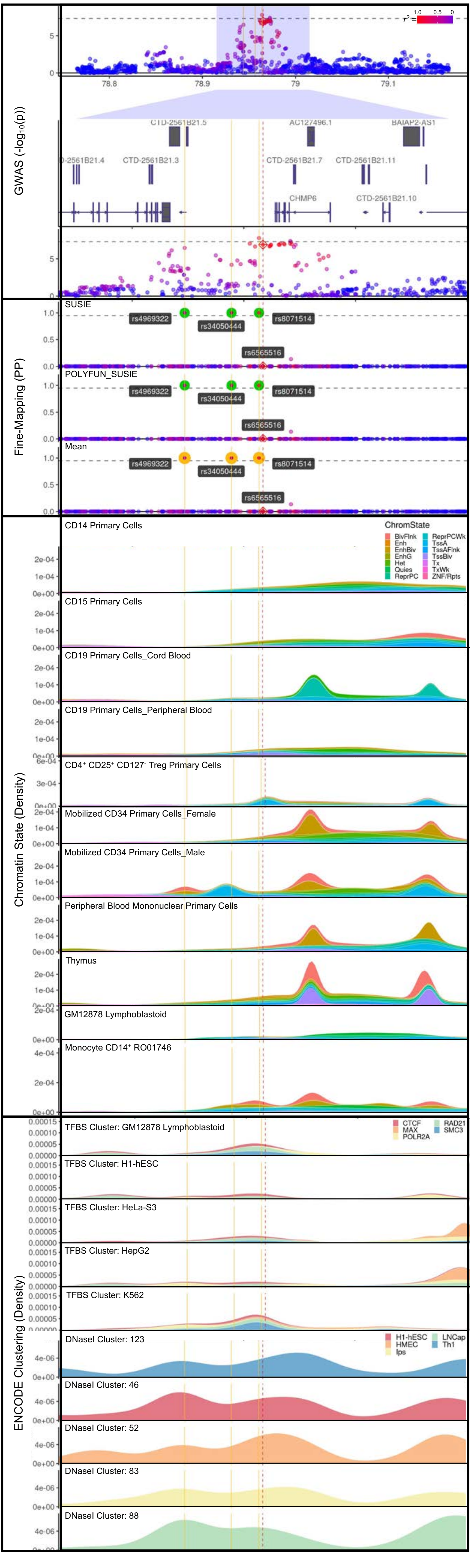

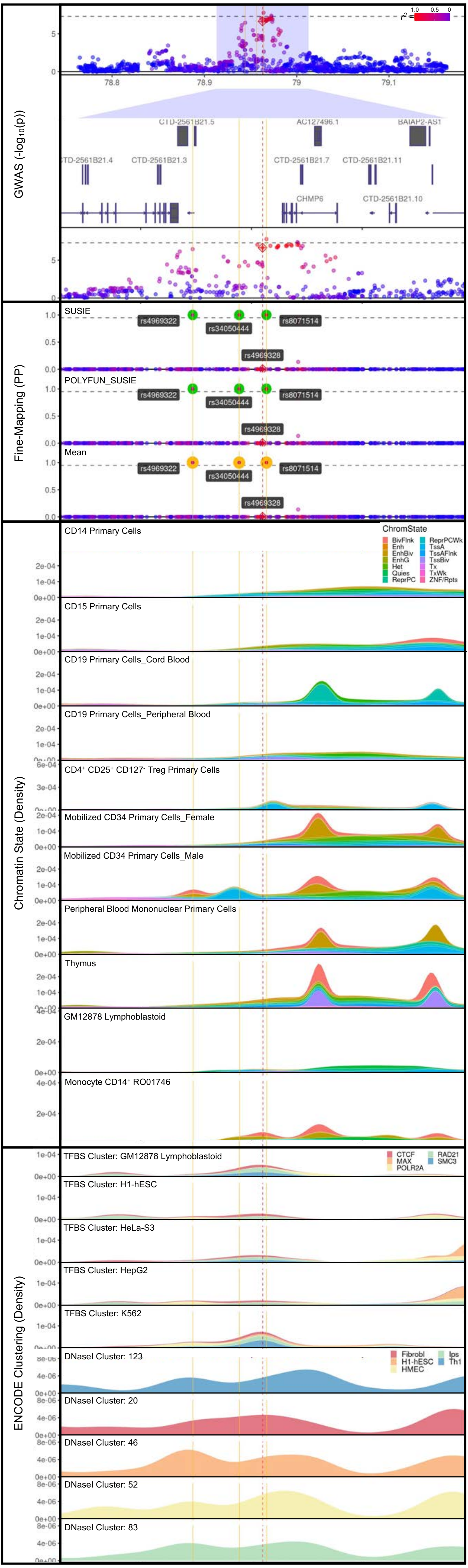

CHMP6_rs4969328
GWAS (-log10(p))
Fine-Mapping (PP)
SUSIE
POLYFUN_SUSIE
Mean
rs4969322
rs34050444
rs8071514
rs4969328
CTD-2561B21.5
AC127496.1
BAIAP2-AS1
CTD-2561B21.4
CTD-2561B21.3
CTD-2561B21.7
CTD-2561B21.11
CHMP6
CTD-2561B21.10
Chromatin State (Density)
ChromState
BivFlnk
Enh
EnhBiv
EnhG
Het
Quies
ReprPC
ReprPCWk
TssA
TssAFlnk
TssBiv
Tx
TxWk
ZNF/Rpts
CD14 Primary Cells
CD15 Primary Cells
CD19 Primary Cells_Cord Blood
CD19 Primary Cells_Peripheral Blood
CD4+ CD25+ CD127- Treg Primary Cells
Mobilized CD34 Primary Cells_Female
Mobilized CD34 Primary Cells_Male
Peripheral Blood Mononuclear Primary Cells
Thymus
GM12878 Lymphoblastoid
Monocyte CD14+ RO01746
ENCODE Clustering (Density)
TFBS Cluster: GM12878 Lymphoblastoid
CTCF
MAX
POLR2A
RAD21
SMC3
TFBS Cluster: H1-hESC
TFBS Cluster: HeLa-S3
TFBS Cluster: HepG2
TFBS Cluster: K562
DNaseI Cluster: 123
Fibrobl
H1-hESC
HMEC
Ips
Th1
DNaseI Cluster: 20
DNaseI Cluster: 46
DNaseI Cluster: 52
DNaseI Cluster: 83

CHMP6_rs4969331

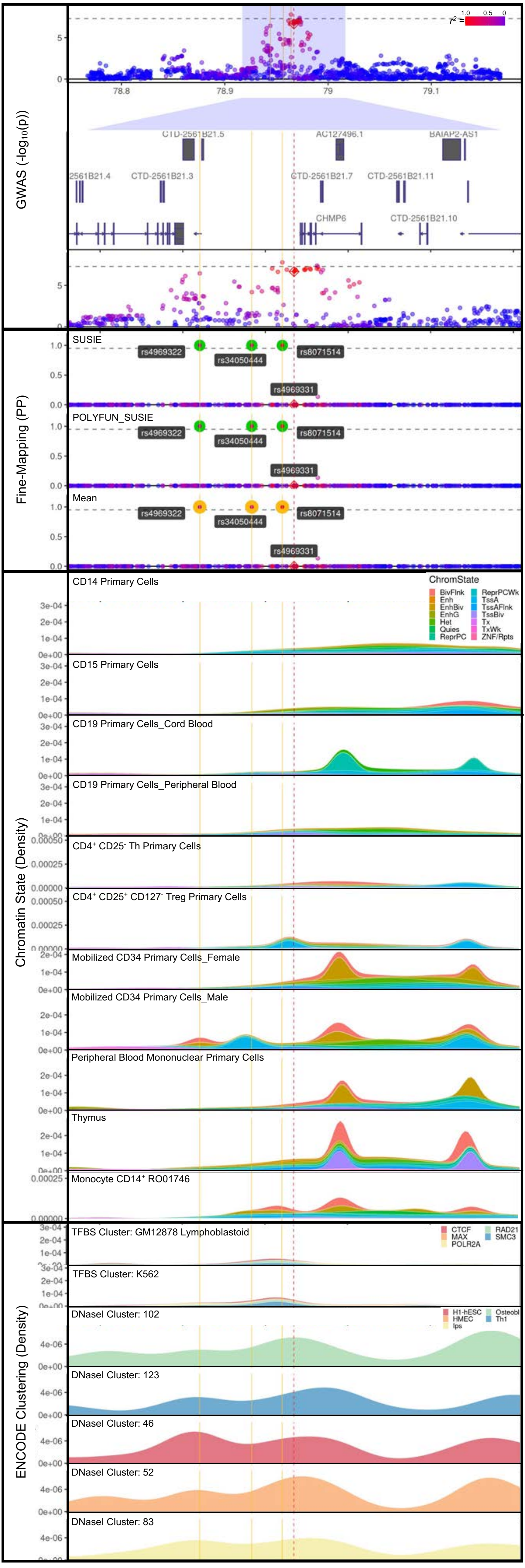

CHMP6_rs6565518

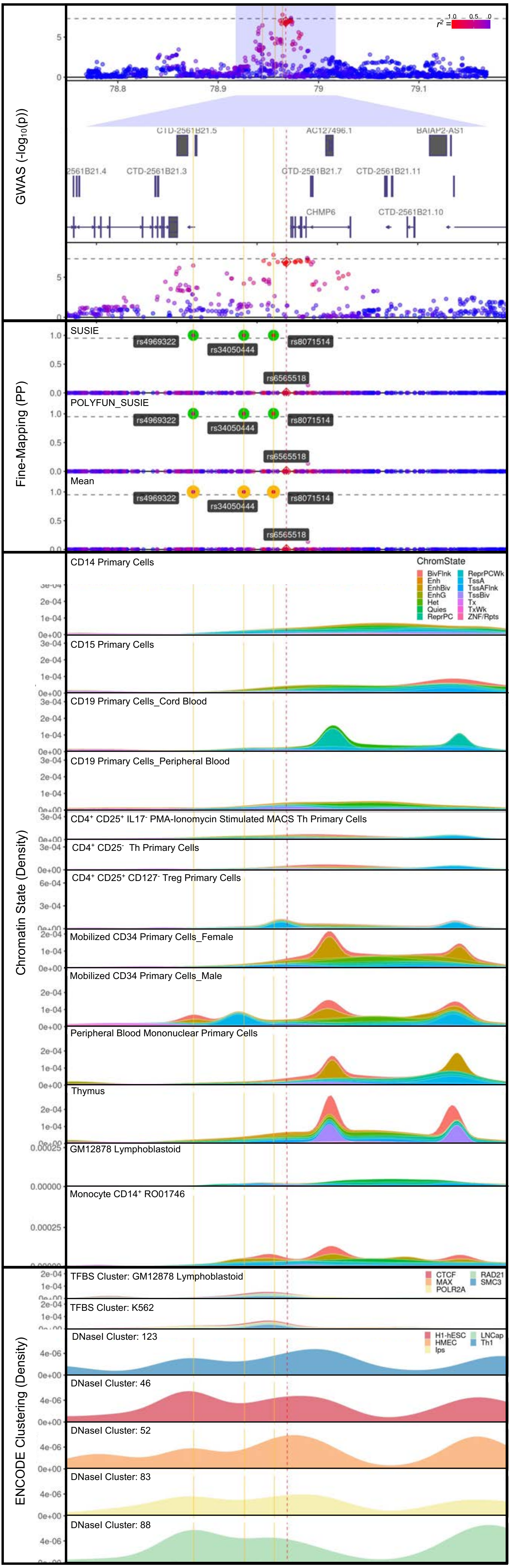

Supplementary Figure 9: Conditional analysis, posterior probability analysis, chromatin looping, and eQTL mapping in the *PRDM1-ATG5* locus.

(a) Conditional analysis was performed, adjusting for rs526531. A residual effect was for the top SNP, rs11152944.

(b) Posterior probabilities distribution of variants in *PRDM1-ATG5* locus, identifying rs548234 as the most probable SNP.

(c-e) The pairwise *D'* (**c**), r^2 (**d**), and haplotypes with their frequencies of the top and functional variants in the region are displayed (**e**).

(f) Circos plot mapping the zoom regional Manhattan plot of the imputed GWAS data for the *PRDM1-ATG5* association on Chromosome 6 (outer most layer). All SNPs with a $-\log_{10}$(p-value) <0.05 are shown in black, or colored based on r^2 (red: r^2>0.08; orange: r^2>0.06). The index SNP of the *PRDM1-ATG5* association, rs526531, is indicated. The outer circle displays the chromosome coordinate of the *PRDM1-ATG5* risk locus highlighted in blue. Genes that are eQTLs or exhibit chromatin interaction by Hi-C in GM12878 Epstein-Barr virus (EBV)-transformed B lymphocytes are reported on the inner circles in green or orange font, respectively, and are shown as links colored green or orange, respectively, on the inner most layer.

(g) Lack of epigenetic marks suggest that the index Sjögren-SNP rs526531 is not likely function. For details, see Supplementary Table 25.

(h-l) Sjögren-SNPs rs11152966 (**h**), rs533733 (**i**), rs548234 (**j**), rs4946728 (**k**), and rs7768653 (**l**), positioned in intergenic space between *PRDM1* and *ATG5*, all exhibit epigenetic enhancer and promoter marks across several immune cell types and share several eQTLs, including *ATG5* and *PRDM1* in several immune cell types and *ATG5* in the minor salivary gland. *PRDM1* encodes B-lymphocyte induced maturation protein 1 (BLIMP1), which has several regulatory roles in innate and adaptive immune responses. *ATG5* is a prominent regulator of autophagy. Lack of reported chromatin-chromatin interactions in all but the EBV B cell types suggest that stimulation may be required for interactions between the enhancer and *PRDM1* or *ATG5* promoters. For details, see Supplementary Table 25.

(m) Lack of epigenetic marks suggest that Sjögren-SNP rs4134466 is likely not function, despite being a SNP from the 95% credible set for the *PRDM1-ATG5* region. For details, see Supplementary Table 25.

(n-t) EcholocatoR was used to identify, fine-map and annotate the *PRDM1-ATG5* region after specifying the index SNPs (indicated by red dotted line): rs526531 (**n**), rs11152966 (**o**), rs533733 (**p**), rs548234 (**q**), rs4946728 (**r**), rs7768653 (**s**), and rs4134466 (**t**). Additional SNPs with plausible function were identified and indicated by the dotted yellow lines.

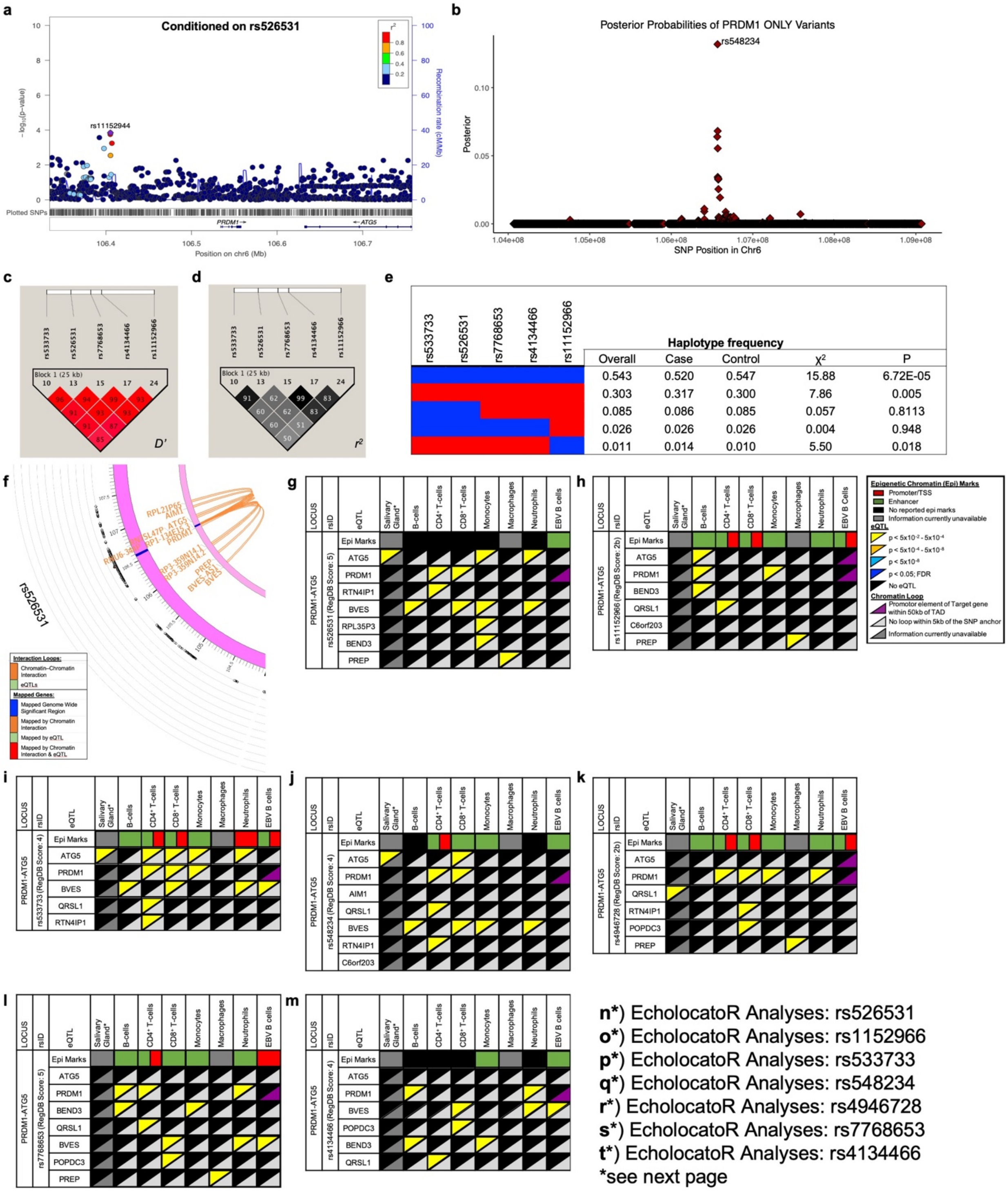

n*) EcholocatoR Analyses: rs526531
o*) EcholocatoR Analyses: rs1152966
p*) EcholocatoR Analyses: rs533733
q*) EcholocatoR Analyses: rs548234
r*) EcholocatoR Analyses: rs4946728
s*) EcholocatoR Analyses: rs7768653
t*) EcholocatoR Analyses: rs4134466
*see next page

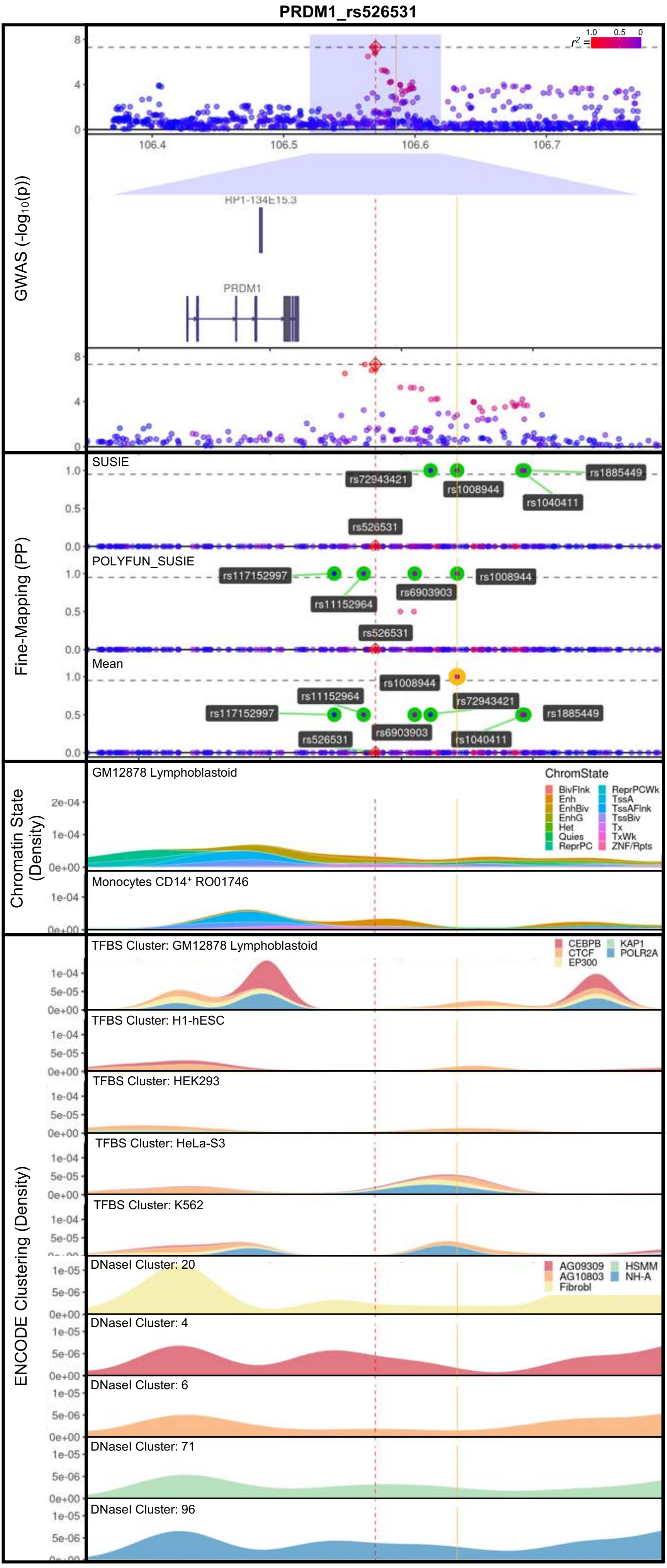

PRDM1_rs526531
GWAS (-log10(p))
Fine-Mapping (PP)
Chromatin State (Density)
ENCODE Clustering (Density)
RP1-134E15.3
PRDM1
SUSIE
POLYFUN_SUSIE
Mean
rs72943421
rs1008944
rs1885449
rs1040411
rs526531
rs117152997
rs11152964
rs6903903
GM12878 Lymphoblastoid
Monocytes CD14+ RO01746
ChromState
BivFlnk
Enh
EnhBiv
EnhG
Het
Quies
ReprPC
ReprPCWk
TssA
TssAFlnk
TssBiv
Tx
TxWk
ZNF/Rpts
TFBS Cluster: GM12878 Lymphoblastoid
TFBS Cluster: H1-hESC
TFBS Cluster: HEK293
TFBS Cluster: HeLa-S3
TFBS Cluster: K562
CEBPB
CTCF
EP300
KAP1
POLR2A
DNaseI Cluster: 20
DNaseI Cluster: 4
DNaseI Cluster: 6
DNaseI Cluster: 71
DNaseI Cluster: 96
AG09309
AG10803
Fibrobl
HSMM
NH-A

PRDM1_rs11152966

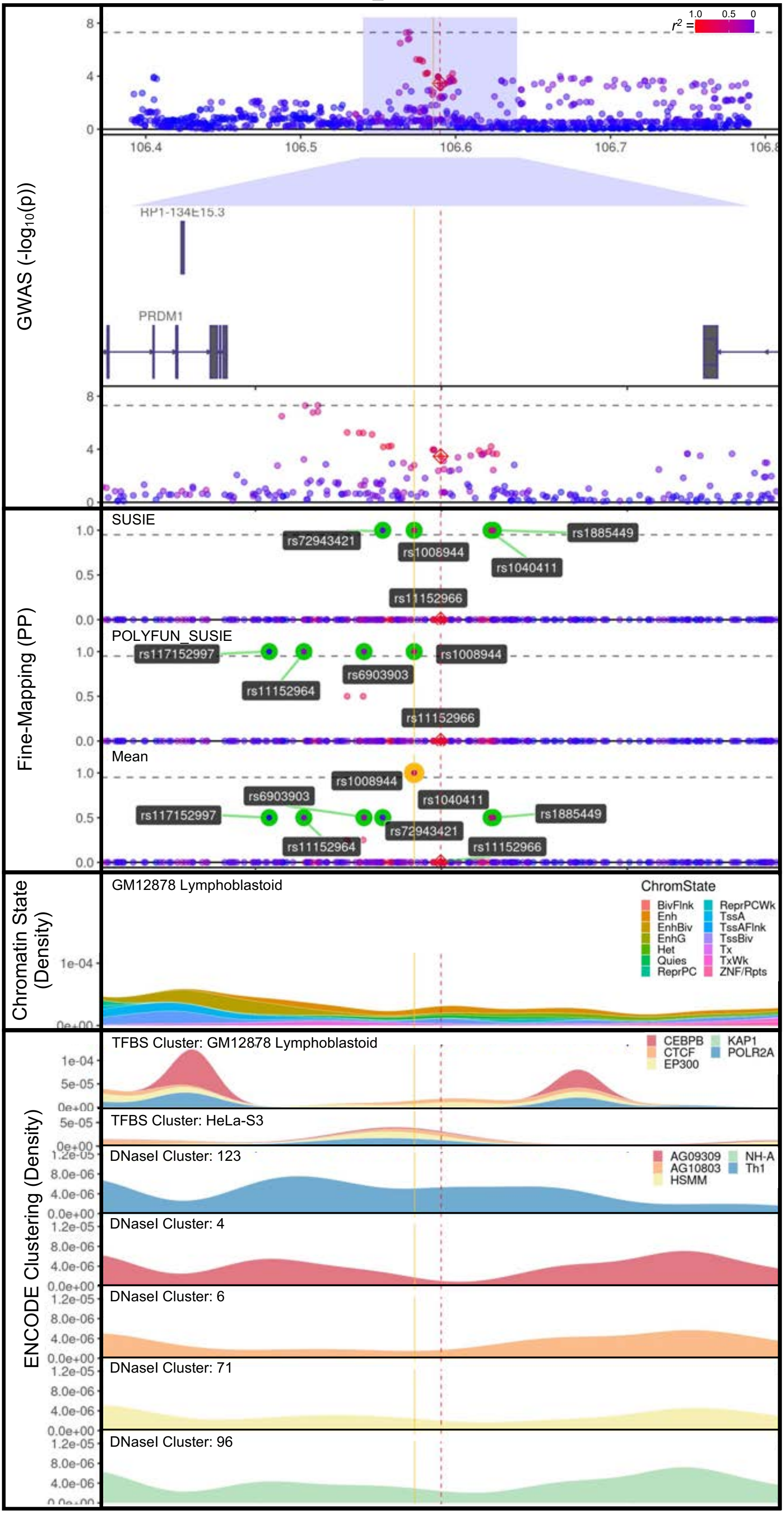

PRDM1_rs533733

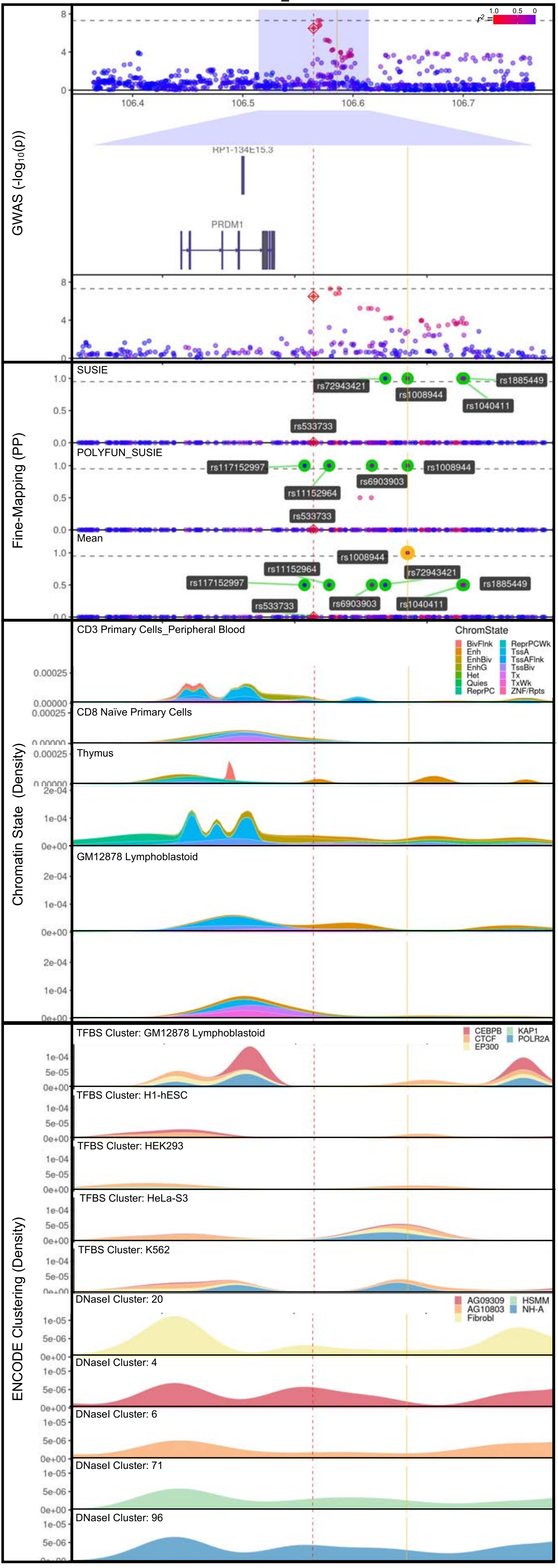

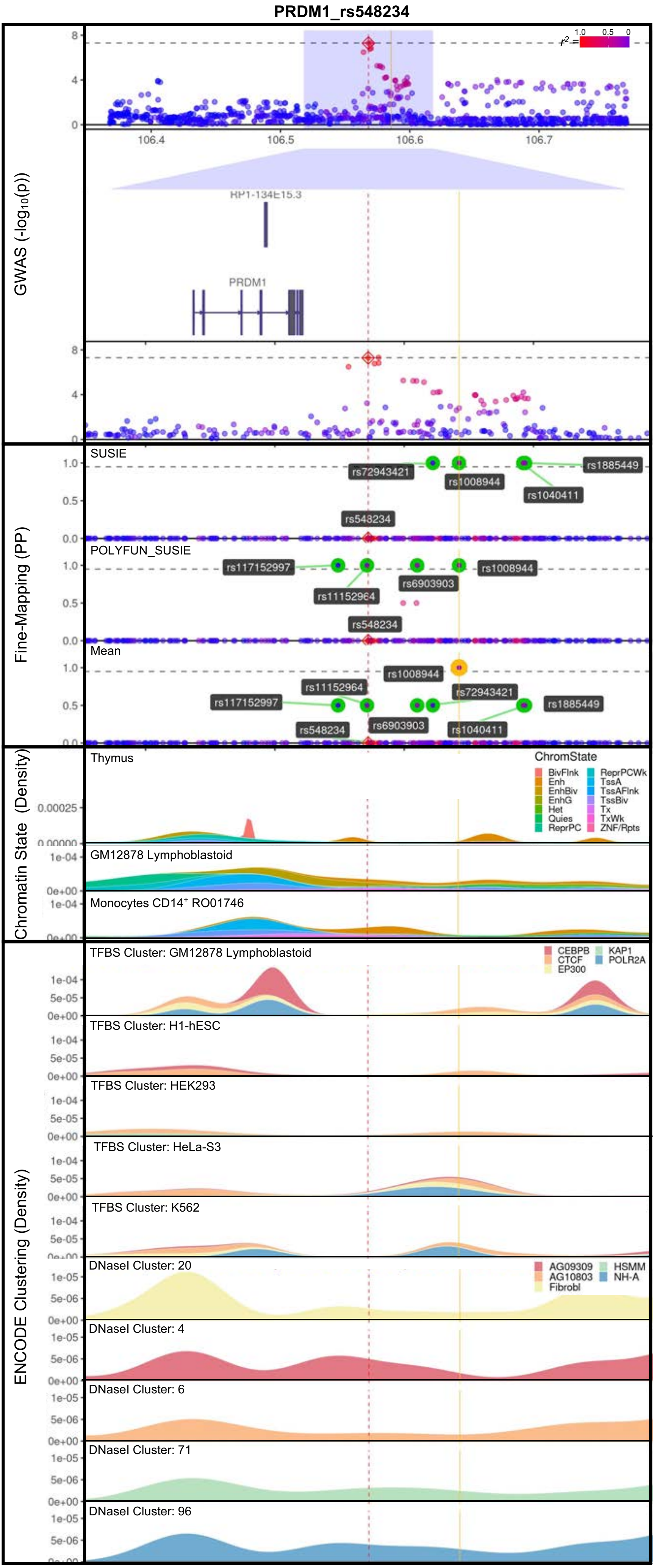
PRDM1_rs548234
GWAS (-log10(p))
106.4
106.5
106.6
106.7
RP1-134E15.3
PRDM1
Fine-Mapping (PP)
SUSIE
rs72943421
rs1008944
rs1040411
rs1885449
rs548234
POLYFUN_SUSIE
rs117152997
rs11152964
rs6903903
rs1008944
rs548234
Mean
rs1008944
rs11152964
rs72943421
rs117152997
rs1885449
rs548234
rs6903903
rs1040411
Chromatin State (Density)
Thymus
ChromState
BivFlnk
Enh
EnhBiv
EnhG
Het
Quies
ReprPC
ReprPCWk
TssA
TssAFlnk
TssBiv
Tx
TxWk
ZNF/Rpts
GM12878 Lymphoblastoid
Monocytes CD14+ RO01746
ENCODE Clustering (Density)
TFBS Cluster: GM12878 Lymphoblastoid
CEBPB
CTCF
EP300
KAP1
POLR2A
TFBS Cluster: H1-hESC
TFBS Cluster: HEK293
TFBS Cluster: HeLa-S3
TFBS Cluster: K562
DNaseI Cluster: 20
AG09309
AG10803
Fibrobl
HSMM
NH-A
DNaseI Cluster: 4
DNaseI Cluster: 6
DNaseI Cluster: 71
DNaseI Cluster: 96

PRDM1_rs4946728

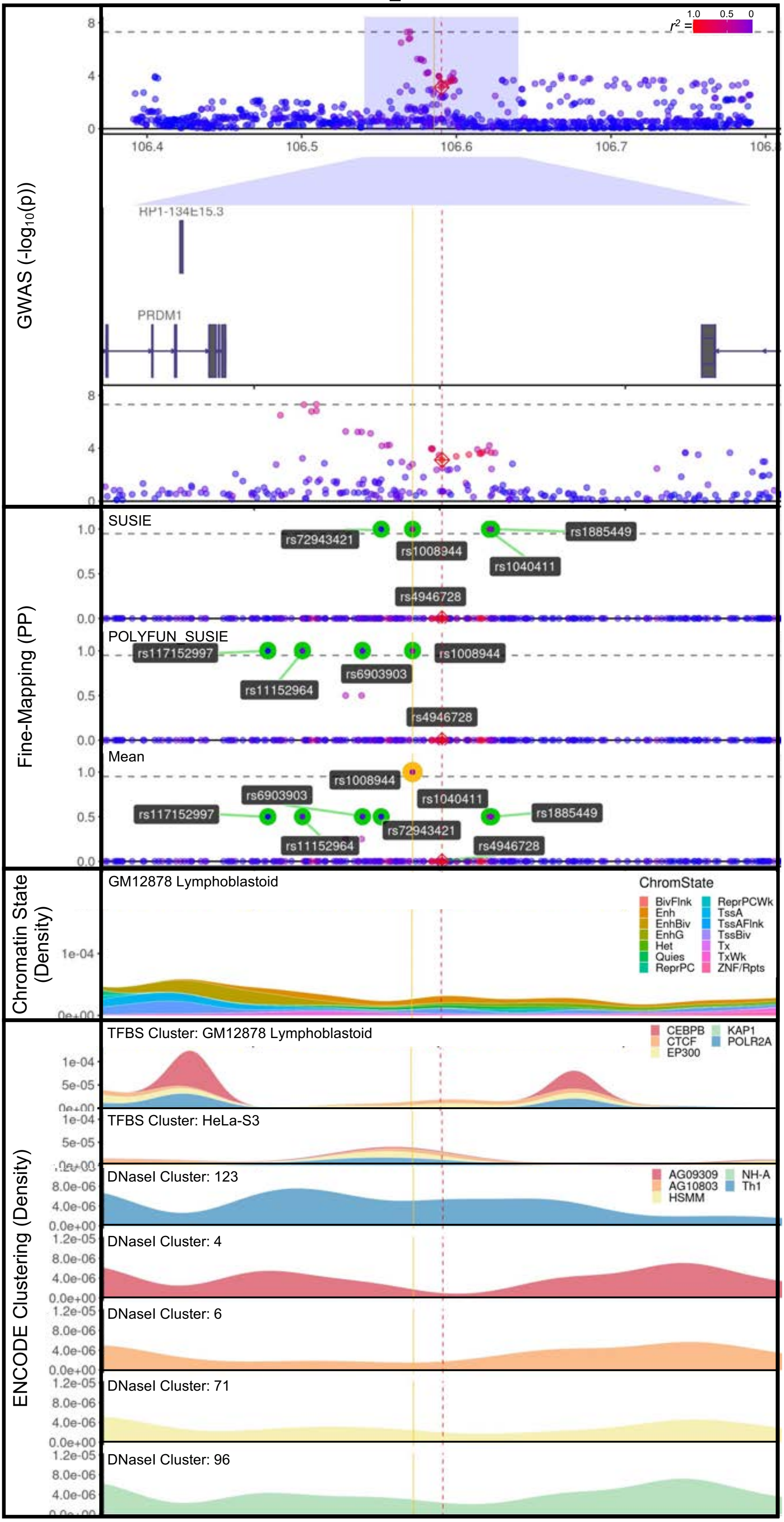

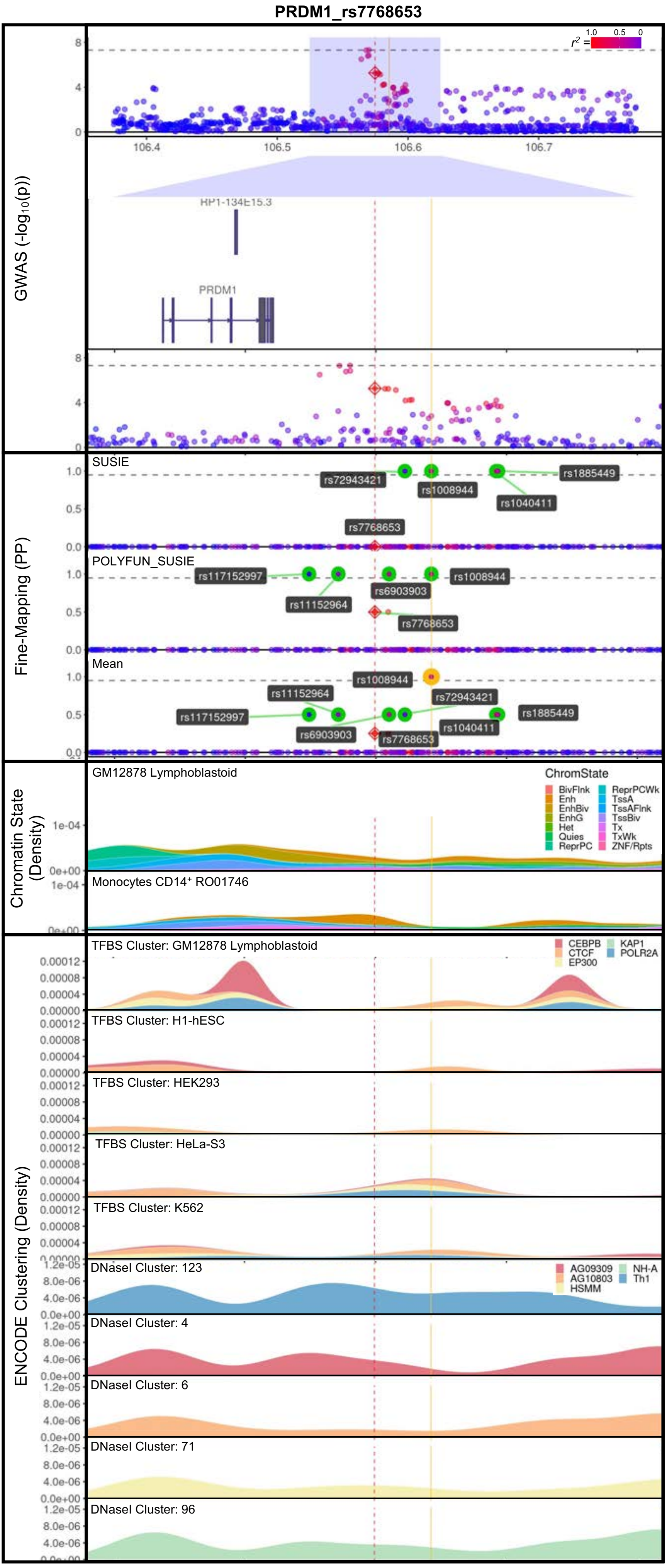
PRDM1_rs7768653
GWAS (-log10(p))
Fine-Mapping (PP)
Chromatin State (Density)
ENCODE Clustering (Density)
106.4
106.5
106.6
106.7
RP1-134E15.3
PRDM1
SUSIE
rs72943421
rs1008944
rs1040411
rs1885449
rs7768653
POLYFUN_SUSIE
rs117152997
rs11152964
rs6903903
Mean
GM12878 Lymphoblastoid
ChromState
BivFlnk
Enh
EnhBiv
EnhG
Het
Quies
ReprPC
ReprPCWk
TssA
TssAFlnk
TssBiv
Tx
TxWk
ZNF/Rpts
Monocytes CD14+ RO01746
TFBS Cluster: GM12878 Lymphoblastoid
CEBPB
CTCF
EP300
KAP1
POLR2A
TFBS Cluster: H1-hESC
TFBS Cluster: HEK293
TFBS Cluster: HeLa-S3
TFBS Cluster: K562
DNaseI Cluster: 123
AG09309
AG10803
HSMM
NH-A
Th1
DNaseI Cluster: 4
DNaseI Cluster: 6
DNaseI Cluster: 71
DNaseI Cluster: 96

PRDM1_rs4134466

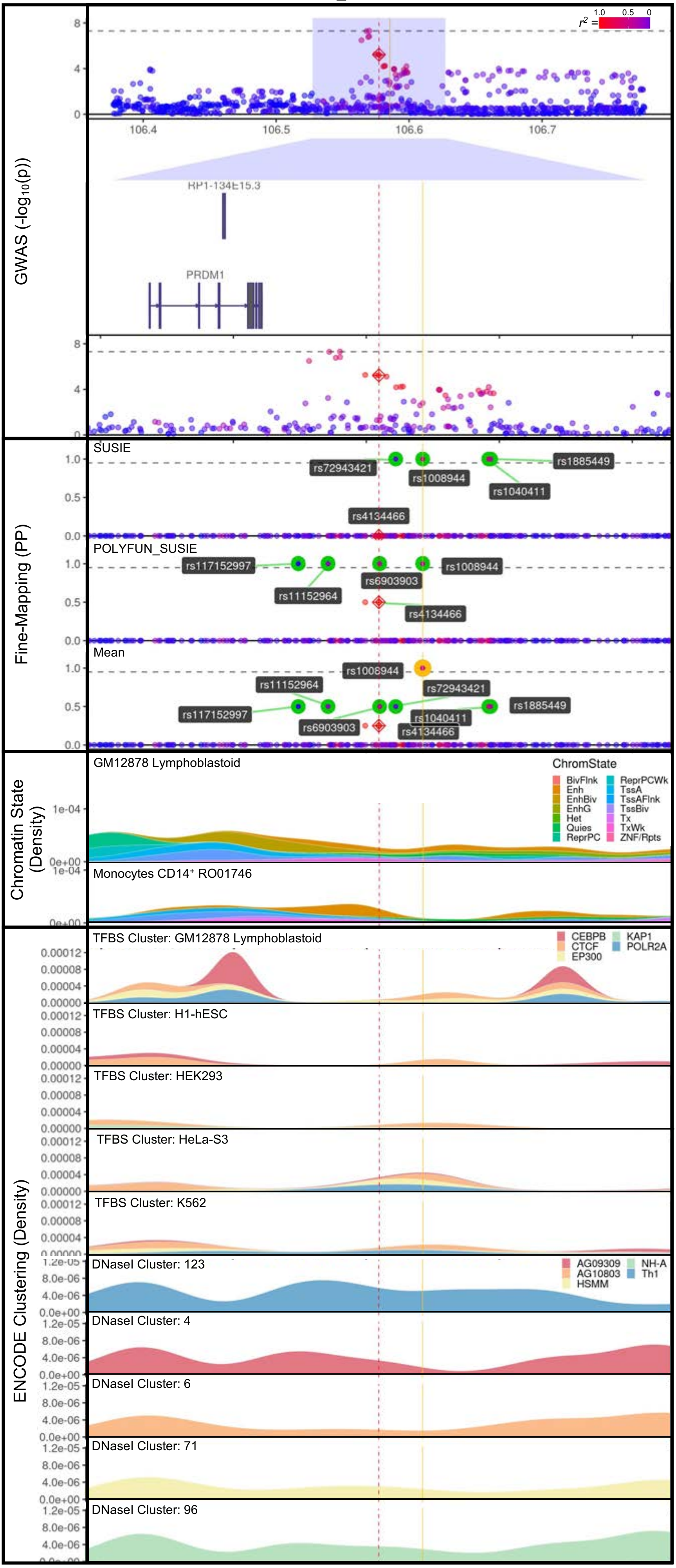

Supplementary Figure 10: Conditional analysis, posterior probability analysis, chromatin looping, and eQTL mapping in the *PTTG1-MIR146A* locus.

(a) Conditional analysis was performed, adjusting for rs2431098. No residual effects were observed in the region of association.

(b) Posterior probabilities distribution of variants in *PTTG1-MIR146A* locus, identifying rs2431098 as the most probable SNP.

(c-e) The pairwise *D’* (**c**), r^2 (**d**), and haplotypes with their frequencies of the top and functional variants in the region are displayed (**e**).

(f) Circos plot mapping the zoom regional Manhattan plot of the imputed GWAS data for the *PTTG1-MIR146A* association on Chromosome 5 (outer most layer). All SNPs with a -$\log_{10}$(p-value) <0.05 are shown in black, or colored based on r^2 (red: r^2>0.08; orange: r^2>0.06). The index SNP of the *PTTG1-MIR146A* association, rs2431697, is indicated. The outer circle displays the chromosome coordinate of the *PTTG1-MIR146A* risk locus highlighted in blue. Genes that are eQTLs or exhibit chromatin interaction by Hi-C in GM12878 Epstein-Barr virus (EBV)-transformed B lymphocytes are reported on the inner circles in green or orange font, respectively, and are shown as links colored green or orange, respectively, on the inner most layer. The index gene for the *PTTG1-MIR146A* association are colored in blue.

(g, h) Sjögren-SNPs rs2431697 (**g**) and rs2431099 (**h**), positioned in an intergenic region 3’ of *PTTG1*, exhibited limited cell type-specific epigenetic enhancer marks in $CD4^+$ T cells (green rectangles) and several eQTLs, including *PWWP2A* in salivary gland (top yellow triangles), and upstream *PTTG1* and *SLU7* in CD4+ T cells. Chromatin-chromatin interactions in GM12878 EBV B lymphocytes (bottom purple triangle) revealed interactions between this enhancer and the promoter of *NUDCD2*, *HMMR*, and *RP11-541P9.3*, but not with the promoters of *PTTG1* or *SLU7*. For details, see Supplementary Table 28.

(i) Lack of epigenetic marks suggest that Sjögren-SNPs rs2431698 is likely not function, despite being a SNP from the 95% credible set for the region. For details, see Supplementary Table 28.

(j) Annotation of Sjögren-SNPs shown in b-d using the IMPACT model to quantify SNP position in 700 cell-type specific active transcription factor binding sites. Top panel depicts SNP position (blue lines) relative to genomic coordinates (Mb) of the *PTTG1-MIR146A* locus. Bottom panel shows the total number of active transcription factor binding sites detected at each SNP.

(k-m) EcholocatoR was used to identify, fine-map and annotate the *PTTG1-MIR146A* region after specifying the index SNPs (indicated by red dotted line): rs2431697 (**k**), rs2431099 (**l**), and rs2431698 (**m**). Additional SNPs with plausible function were identified and indicated by the dotted yellow lines.

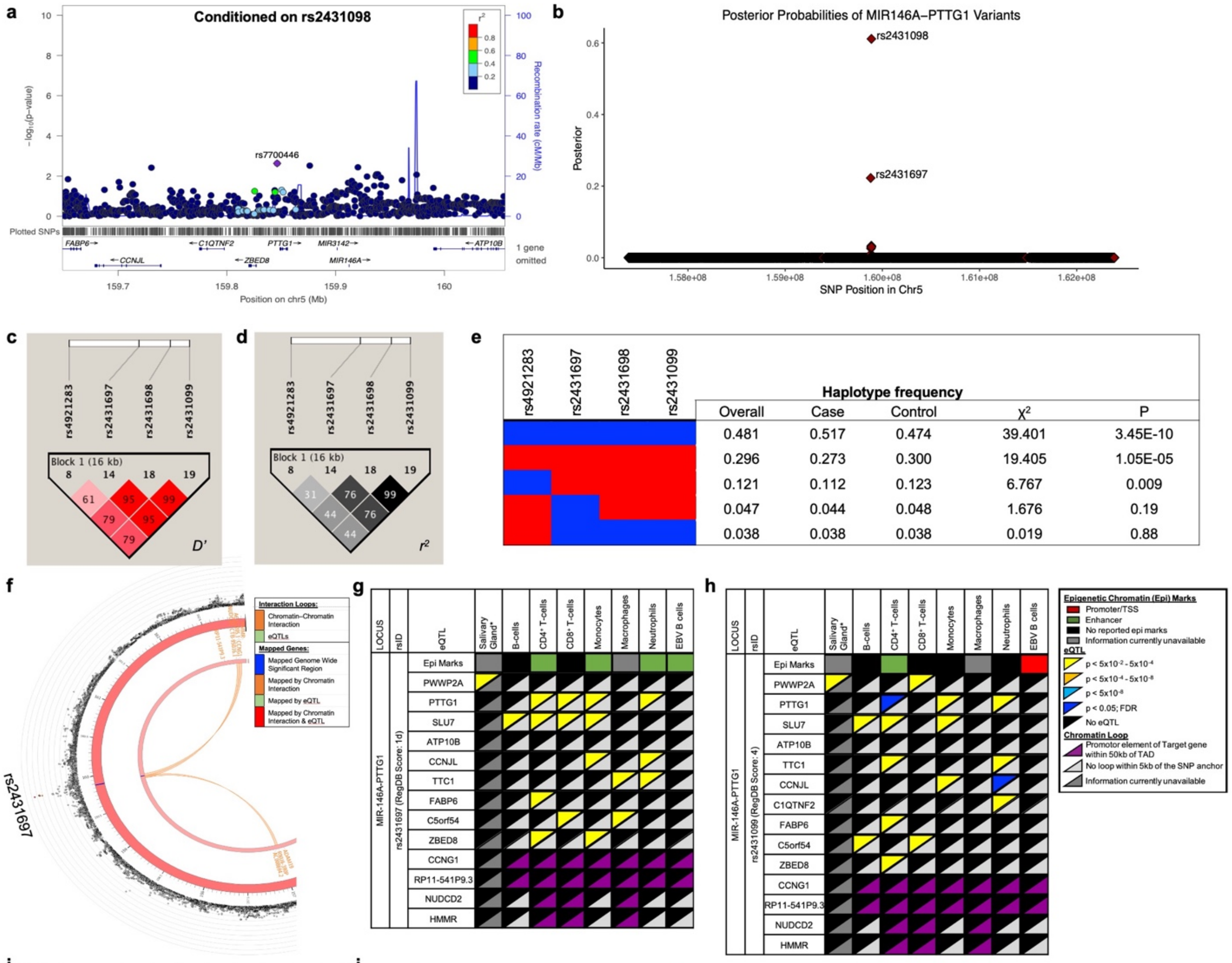

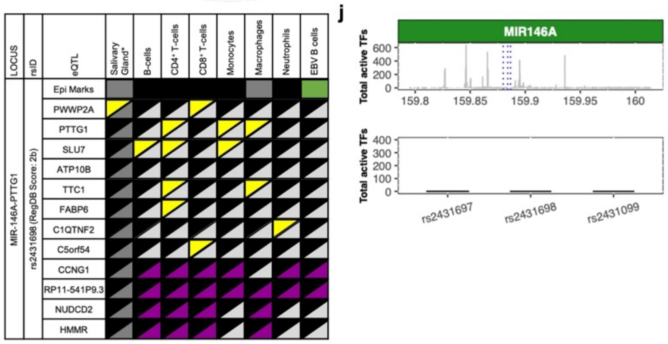

k*) EchoIocatoR Analyses: rs2431697
l*) EchoIocatoR Analyses: rs2431099
m*) EchoIocatoR Analyses: rs2431698
*see next page

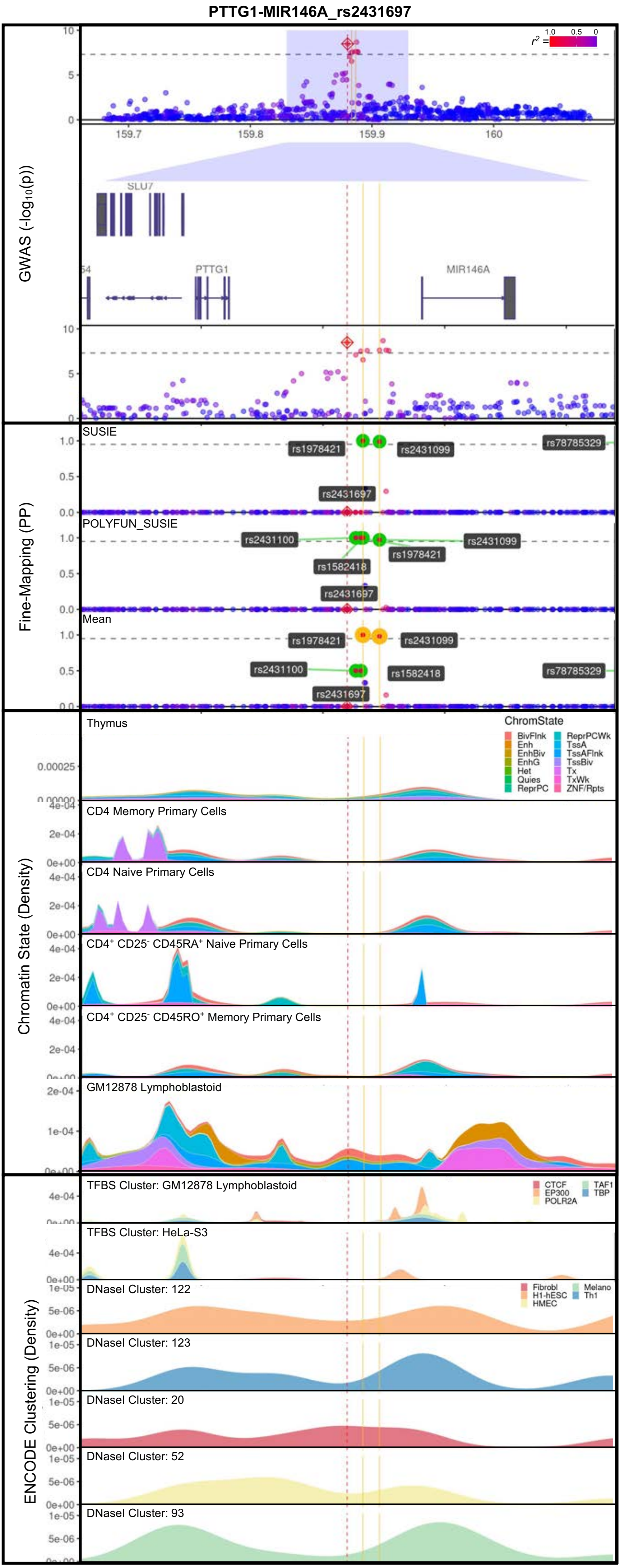

PTTG1-MIR146A_rs2431697
GWAS (-log10(p))
SLU7
PTTG1
MIR146A
Fine-Mapping (PP)
SUSIE
POLYFUN_SUSIE
Mean
rs1978421
rs2431099
rs78785329
rs2431697
rs2431100
rs1582418
Chromatin State (Density)
ChromState
Thymus
CD4 Memory Primary Cells
CD4 Naive Primary Cells
CD4+ CD25- CD45RA+ Naive Primary Cells
CD4+ CD25- CD45RO+ Memory Primary Cells
GM12878 Lymphoblastoid
ENCODE Clustering (Density)
TFBS Cluster: GM12878 Lymphoblastoid
TFBS Cluster: HeLa-S3
DNaseI Cluster: 122
DNaseI Cluster: 123
DNaseI Cluster: 20
DNaseI Cluster: 52
DNaseI Cluster: 93

PTTG1-MIR146A_rs2431099

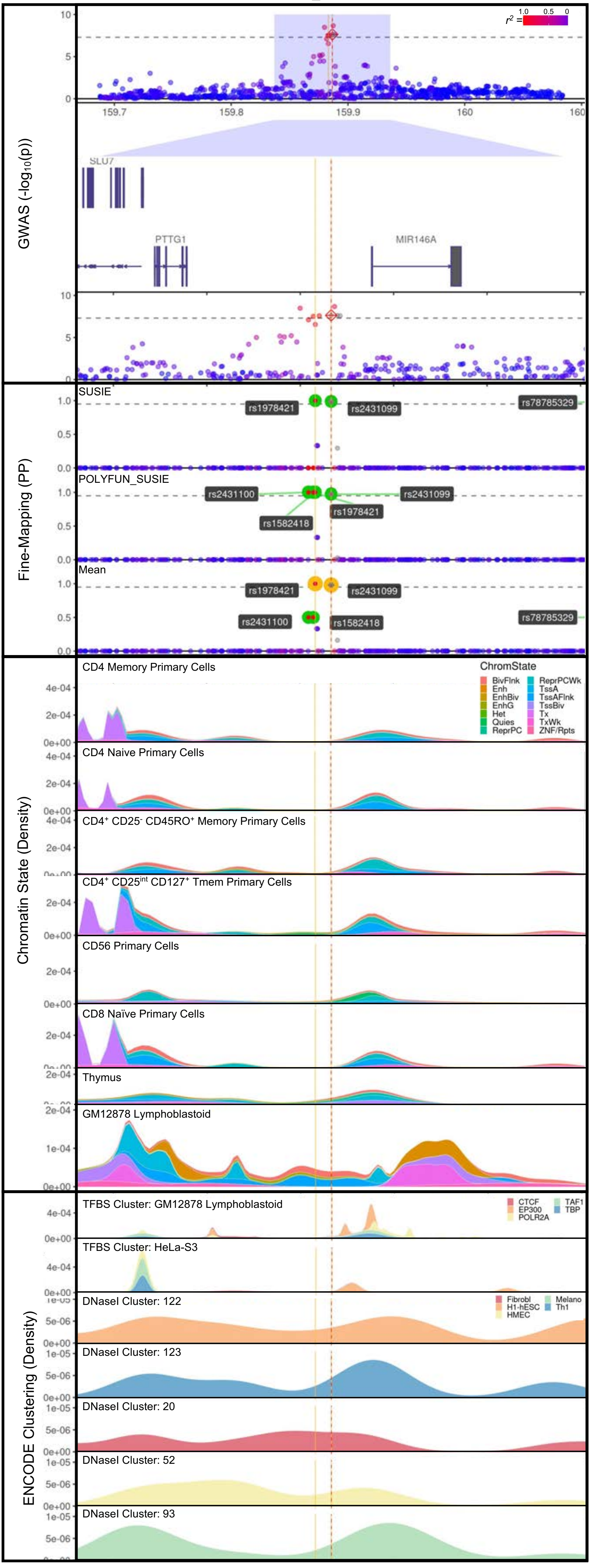

PTTG1-MIR146A_rs2431698

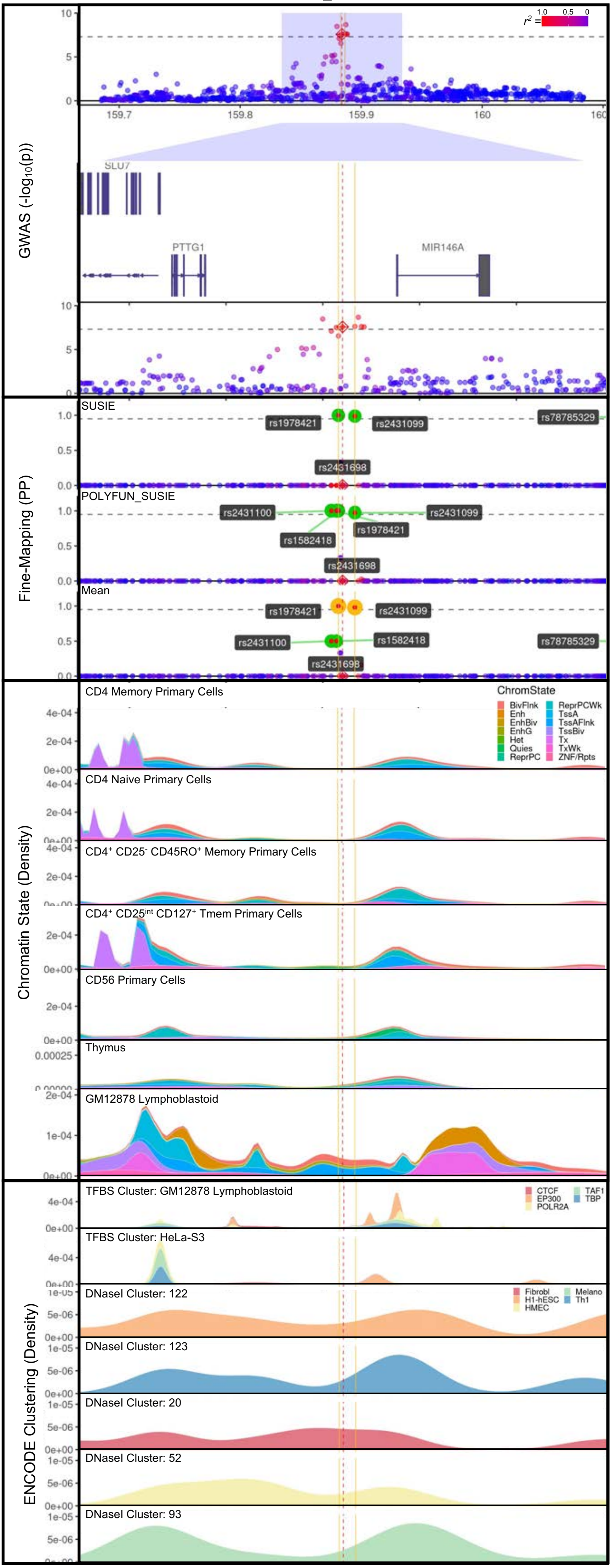

Supplementary Figure 11: Conditional analysis, posterior probability analysis, chromatin looping, and eQTL mapping in the *TNFAIP3* locus.

(a) Conditional analysis was performed, adjusting for rs61117627. A residual effect was for the top SNP, rs17264332.

(b) Posterior probabilities distribution of variants in *TNFAIP3* locus, identifying rs61117627 as the most probable SNP.

(c-e) The pairwise *D'* (**c**), r^2 (**d**), and haplotypes with their frequencies of the top and functional variants in the region are displayed (**e**).

(f) Circos plot mapping the zoom regional Manhattan plot of the imputed GWAS data for the *TNFAIP3* association on Chromosome 6 (outer most layer). All SNPs with a $-\log_{10}$(p-value) <0.05 are shown in black, or colored based on r^2 (red: r^2>0.08; orange: r^2>0.06). The index SNP of the *TNFAIP3* association, rs61117627, is indicated. The outer circle displays the chromosome coordinate of the *TNFAIP3* risk locus highlighted in blue. Genes that are eQTLs or exhibit chromatin interaction by Hi-C in GM12878 Epstein-Barr virus (EBV)-transformed B lymphocytes are reported on the inner circles in green or orange font, respectively, and are shown as links colored green or orange, respectively, on the inner most layer.

(g) Sjögren-SNP rs7749323 is the proxy SNP for the previously described TT>A enhancer 3' of the *TNFAIP3* gene. Consistent with reports, rs7749323 exhibited epigenetic enhancer marks, but minimal chromatin-chromatin interactions or eQTLs for *TNFAIP3*. rs7749323 is an eQTL for *IFNGR1* in the minor salivary gland. Looping data is currently unavailable for the salivary gland but looping data in EBV B cells revealed an interaction between the TT>A enhancer region and the *IFNGR1* promoter upstream. IFNγ signaling plays a prominent role in the inflammatory pathways that drive SS pathogenesis in the salivary gland. For details, see Supplementary Table 31.

(h, i) Sjögren-SNPs rs10499197 (**h**) and rs58905141 (**i**), positioned in an intergenic enhancer 5' of the TNFAIP3 promoter, exhibit epigenetic enhancer and promoter marks across several immune cell types and share several eQTLs, including *CCDC28A* in the minor salivary gland. SNP rs10499197 (**h**) also has coalescence of eQTL and chromatin-chromatin interactions with the *TNFAIP3* promoter in several immune cell types, suggesting that this enhancer may function to modulate expression of *TNFAIP3*, an important regulator of inflammatory signaling. For details, see Supplementary Table 31.

(j) Sjögren-SNP rs5029937, positioned in an intronic region of *TNFAIP3*, exhibit epigenetic enhancer and promoter marks and is an eQTL for several genes across several cell types. For details, see Supplementary Table 31.

(k) Sjögren-SNP rs5029924 is positioned in the *TNFAIP3* promoter region and exhibits epigenetic promoter marks across several immune cell types. For details, see Supplementary Table 31.

(l) The coding variant, rs2230926, in *TNFAIP3* is also a reported eQTL for *TNFAIP3* in several immune cell types. For details, see Supplementary Table 30.

(m-s) EcholocatoR was used to identify, fine-map and annotate the *TNFAIP3* region after specifying the index SNPs (indicated by red dotted line): rs7749323 (**m**), rs10499197 (**n**) rs58905141 (**o**), rs5029937 (**p**), rs5029924(**q**), rs2230926 (**r**), and rs61117627 (**s**). Additional SNPs with plausible function were identified and indicated by the dotted yellow lines.

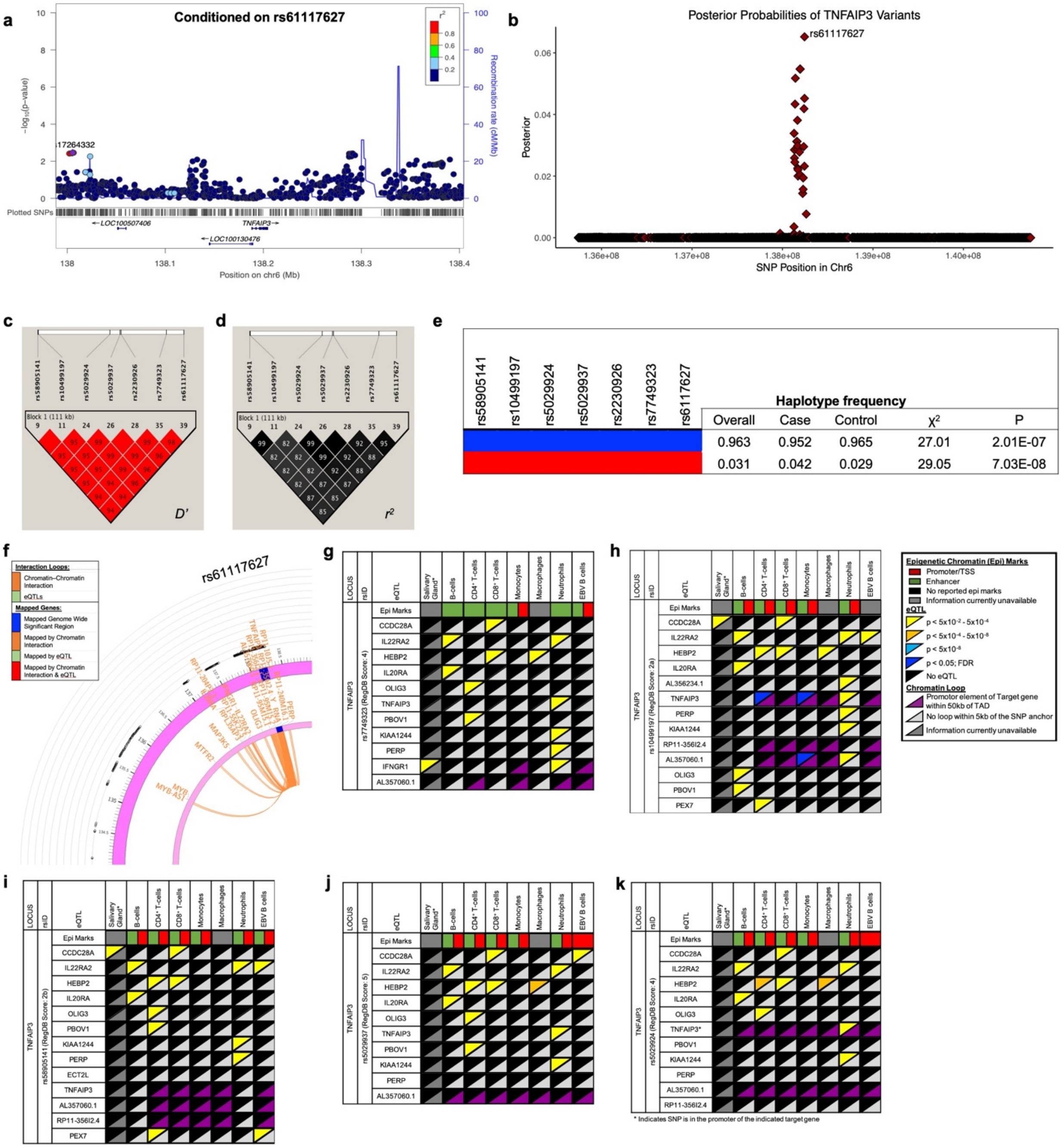

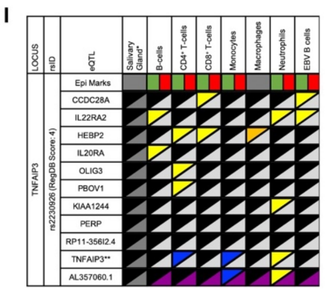

m*) EcholocatoR Analyses: rs7749323
n*) EcholocatoR Analyses: rs10499197
o*) EcholocatoR Analyses: rs58905141
p*) EcholocatoR Analyses: rs5029937
q*) EcholocatoR Analyses: rs5029924
r*) EcholocatoR Analyses: rs2230926
s*) EcholocatoR Analyses: rs61117627
*see next page

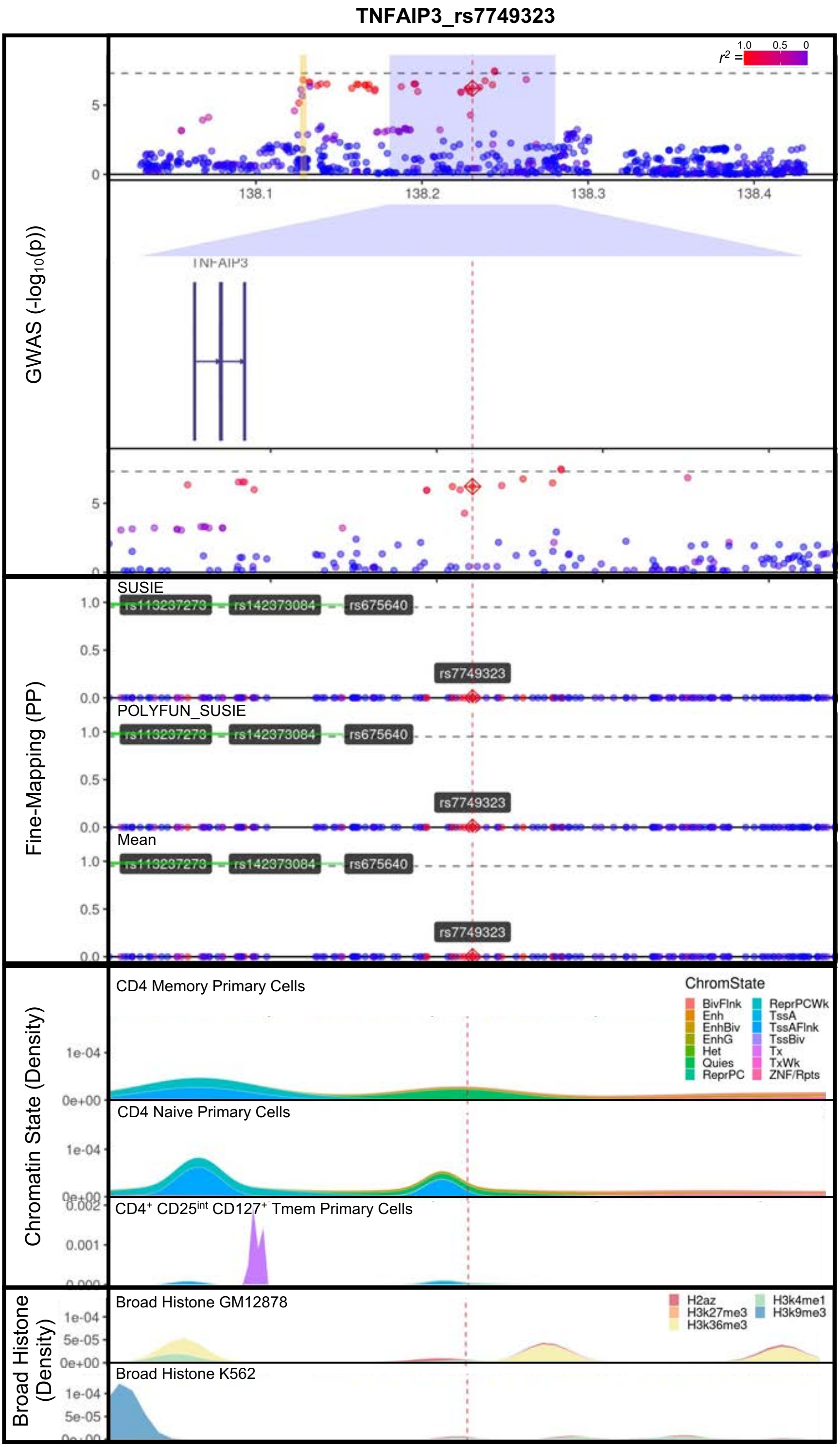
TNFAIP3_rs7749323
GWAS (-log10(p))
Fine-Mapping (PP)
SUSIE
POLYFUN_SUSIE
Mean
rs113237273
rs142373084
rs675640
rs7749323
TNFAIP3
138.1
138.2
138.3
138.4
Chromatin State (Density)
CD4 Memory Primary Cells
CD4 Naive Primary Cells
CD4+ CD25int CD127+ Tmem Primary Cells
ChromState
BivFlnk
Enh
EnhBiv
EnhG
Het
Quies
ReprPC
ReprPCWk
TssA
TssAFlnk
TssBiv
Tx
TxWk
ZNF/Rpts
Broad Histone (Density)
Broad Histone GM12878
Broad Histone K562
H2az
H3k27me3
H3k36me3
H3k4me1
H3k9me3

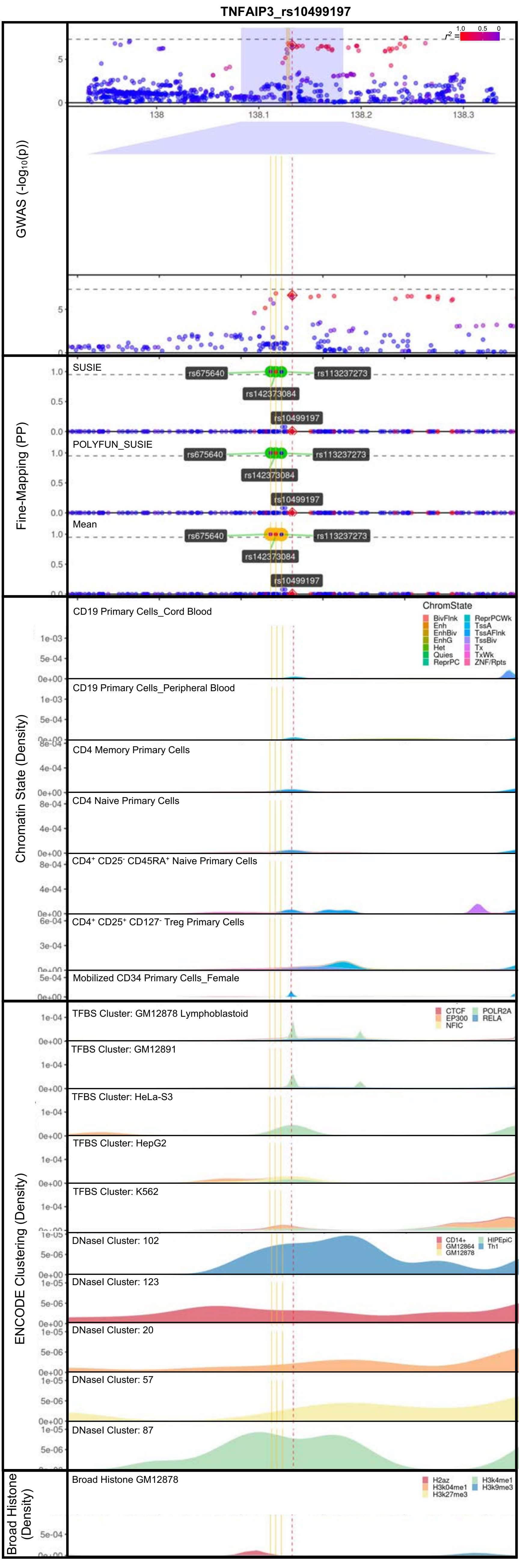

TNFAIP3_rs10499197
GWAS (-log10(p))
Fine-Mapping (PP)
SUSIE
POLYFUN_SUSIE
Mean
rs675640
rs113237273
rs142373084
rs10499197
Chromatin State (Density)
CD19 Primary Cells_Cord Blood
CD19 Primary Cells_Peripheral Blood
CD4 Memory Primary Cells
CD4 Naive Primary Cells
CD4+ CD25- CD45RA+ Naive Primary Cells
CD4+ CD25+ CD127- Treg Primary Cells
Mobilized CD34 Primary Cells_Female
ChromState
BivFlnk
Enh
EnhBiv
EnhG
Het
Quies
ReprPC
ReprPCWk
TssA
TssAFlnk
TssBiv
Tx
TxWk
ZNF/Rpts
ENCODE Clustering (Density)
TFBS Cluster: GM12878 Lymphoblastoid
TFBS Cluster: GM12891
TFBS Cluster: HeLa-S3
TFBS Cluster: HepG2
TFBS Cluster: K562
CTCF
EP300
NFIC
POLR2A
RELA
DNaseI Cluster: 102
DNaseI Cluster: 123
DNaseI Cluster: 20
DNaseI Cluster: 57
DNaseI Cluster: 87
CD14+
GM12864
GM12878
HIPEpiC
Th1
Broad Histone (Density)
Broad Histone GM12878
H2az
H3k04me1
H3k27me3
H3k4me1
H3k9me3

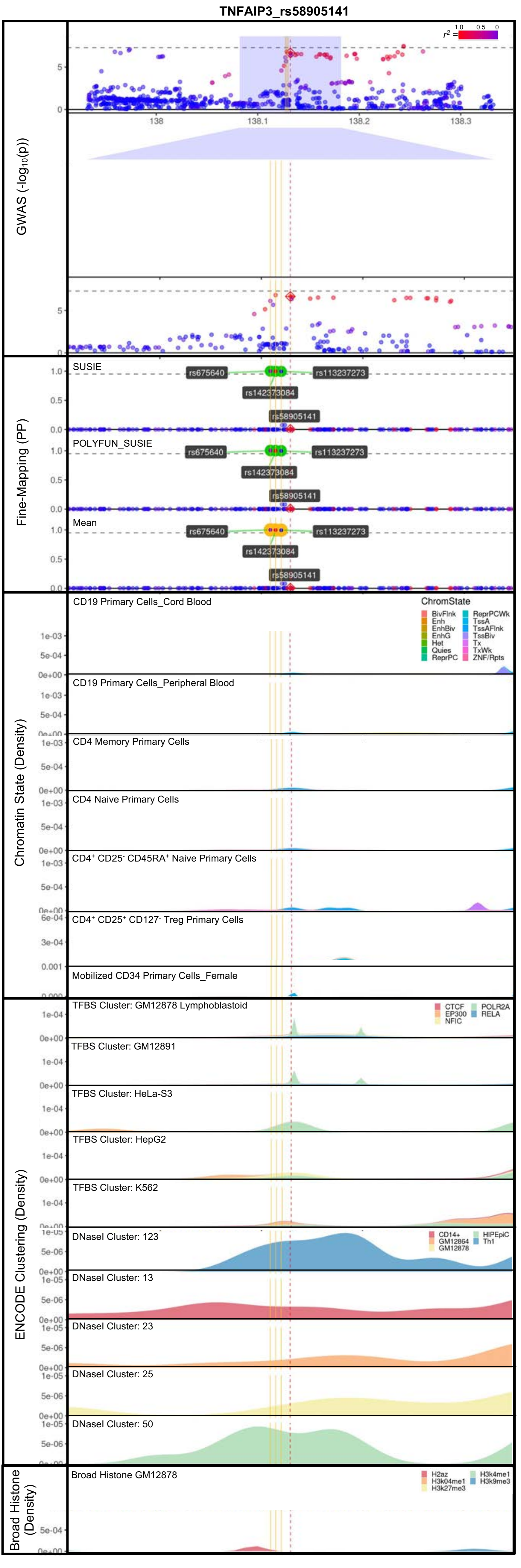

TNFAIP3_rs58905141
GWAS (-log10(p))
Fine-Mapping (PP)
SUSIE
POLYFUN_SUSIE
Mean
rs675640
rs113237273
rs142373084
rs58905141
Chromatin State (Density)
CD19 Primary Cells_Cord Blood
CD19 Primary Cells_Peripheral Blood
CD4 Memory Primary Cells
CD4 Naive Primary Cells
CD4+ CD25- CD45RA+ Naive Primary Cells
CD4+ CD25+ CD127- Treg Primary Cells
Mobilized CD34 Primary Cells_Female
ChromState
BivFlnk
Enh
EnhBiv
EnhG
Het
Quies
ReprPC
ReprPCWk
TssA
TssAFlnk
TssBiv
Tx
TxWk
ZNF/Rpts
ENCODE Clustering (Density)
TFBS Cluster: GM12878 Lymphoblastoid
TFBS Cluster: GM12891
TFBS Cluster: HeLa-S3
TFBS Cluster: HepG2
TFBS Cluster: K562
CTCF
EP300
NFIC
POLR2A
RELA
DNaseI Cluster: 123
DNaseI Cluster: 13
DNaseI Cluster: 23
DNaseI Cluster: 25
DNaseI Cluster: 50
CD14+
GM12864
GM12878
HIPEpiC
Th1
Broad Histone (Density)
Broad Histone GM12878
H2az
H3k04me1
H3k27me3
H3k4me1
H3k9me3

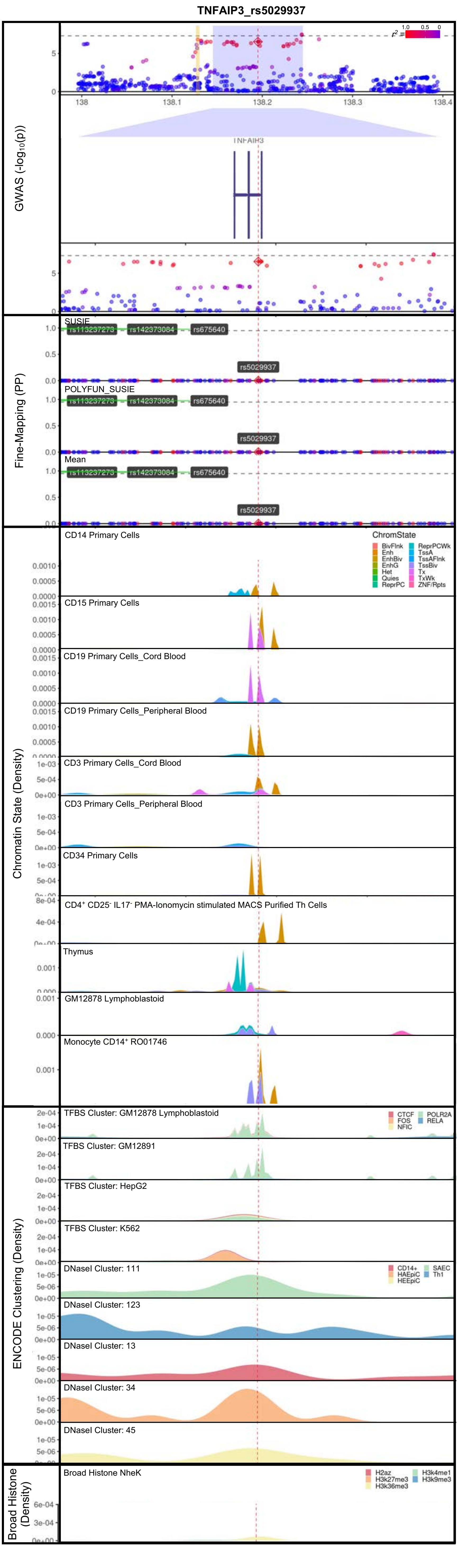

TNFAIP3_rs5029937
GWAS (-log10(p))
TNFAIP3
Fine-Mapping (PP)
SUSIE
POLYFUN_SUSIE
Mean
rs113237273
rs142373084
rs675640
rs5029937
Chromatin State (Density)
ChromState
BivFlnk
Enh
EnhBiv
EnhG
Het
Quies
ReprPC
ReprPCWk
TssA
TssAFlnk
TssBiv
Tx
TxWk
ZNF/Rpts
CD14 Primary Cells
CD15 Primary Cells
CD19 Primary Cells_Cord Blood
CD19 Primary Cells_Peripheral Blood
CD3 Primary Cells_Cord Blood
CD3 Primary Cells_Peripheral Blood
CD34 Primary Cells
CD4+ CD25- IL17- PMA-Ionomycin stimulated MACS Purified Th Cells
Thymus
GM12878 Lymphoblastoid
Monocyte CD14+ RO01746
ENCODE Clustering (Density)
TFBS Cluster: GM12878 Lymphoblastoid
CTCF
FOS
NFIC
POLR2A
RELA
TFBS Cluster: GM12891
TFBS Cluster: HepG2
TFBS Cluster: K562
DNaseI Cluster: 111
CD14+
HAEpiC
HEEpiC
SAEC
Th1
DNaseI Cluster: 123
DNaseI Cluster: 13
DNaseI Cluster: 34
DNaseI Cluster: 45
Broad Histone (Density)
Broad Histone NheK
H2az
H3k27me3
H3k36me3
H3k4me1
H3k9me3

TNFAIP3_rs5029924

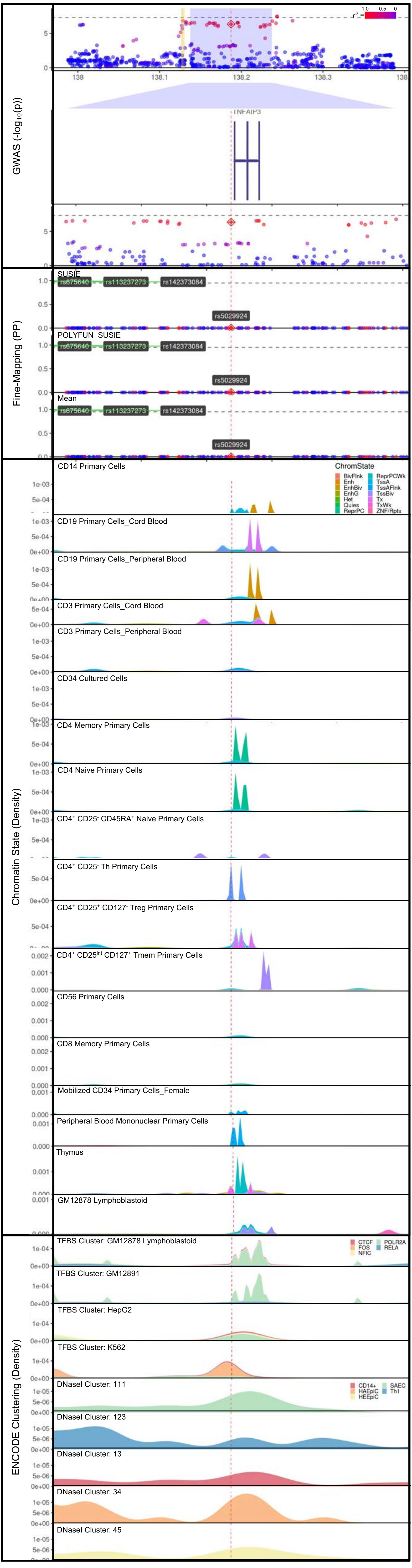

TNFAIP3_rs2230926

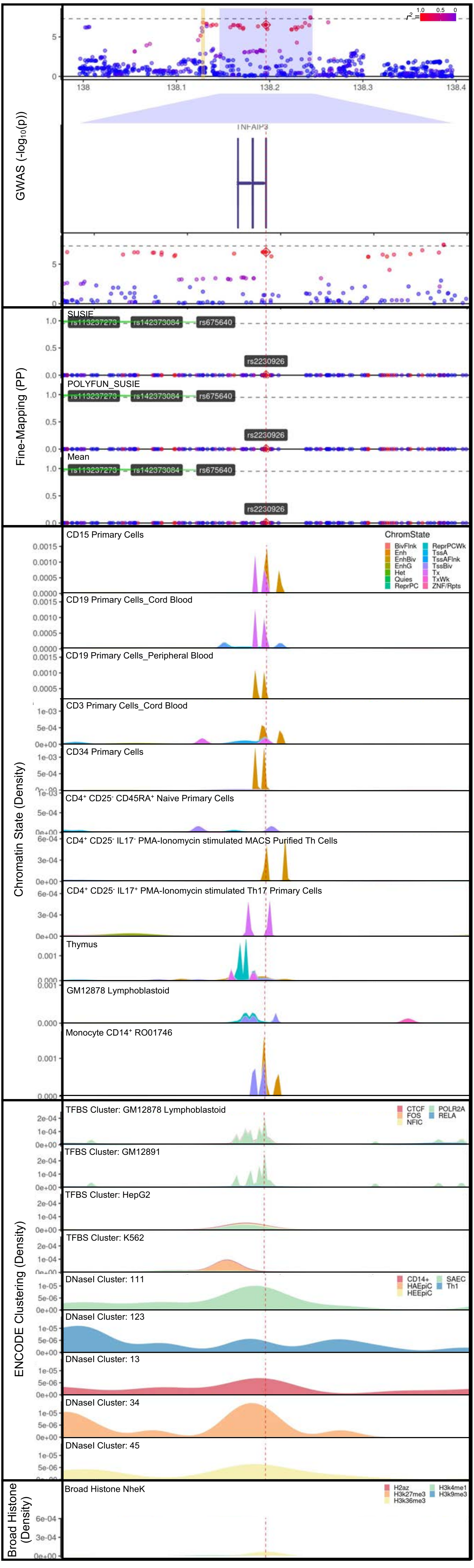

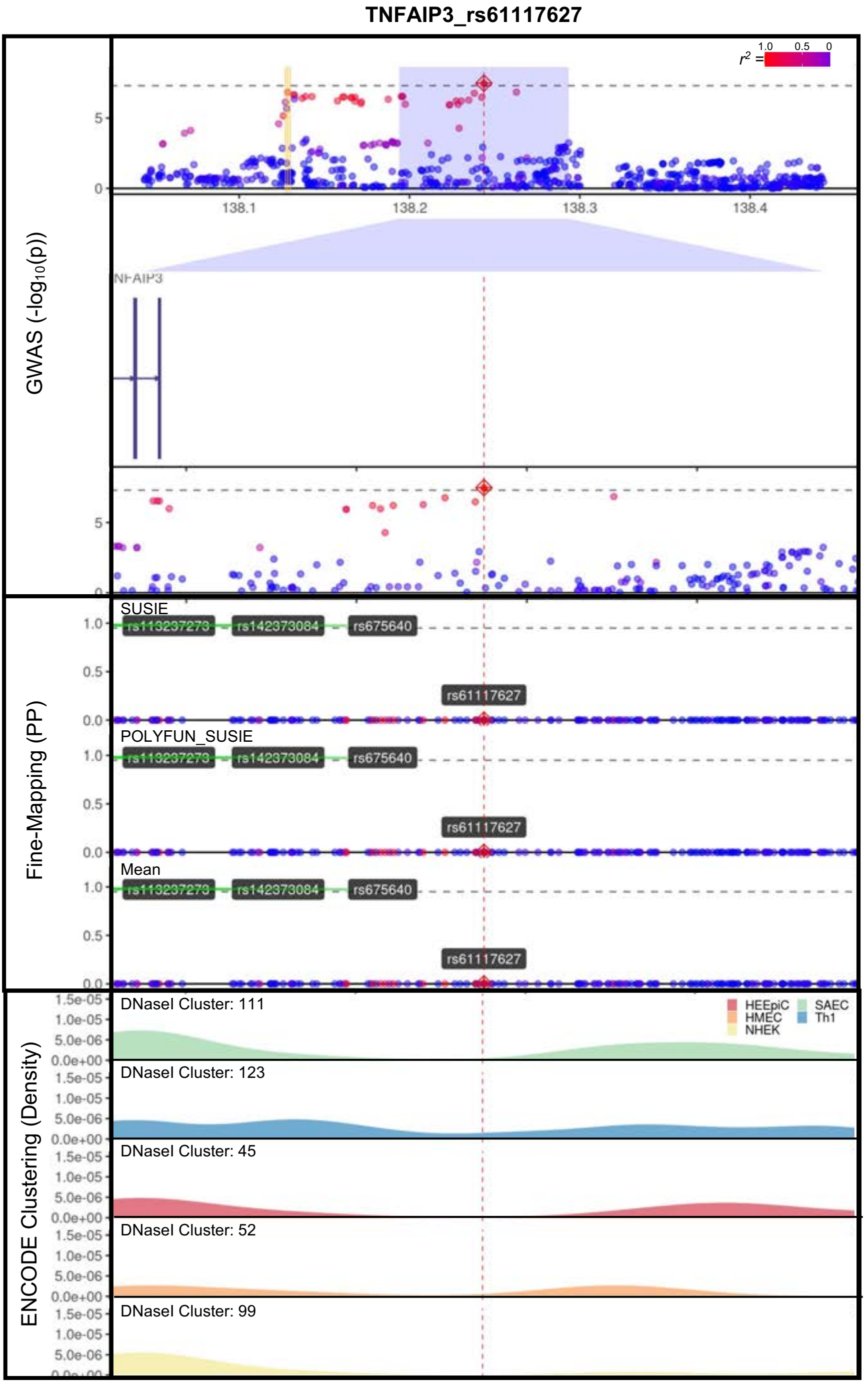
TNFAIP3_rs61117627
GWAS (-log10(p))
r2 =
1.0
0.5
0
138.1
138.2
138.3
138.4
TNFAIP3
Fine-Mapping (PP)
SUSIE
POLYFUN_SUSIE
Mean
rs113237273
rs142373084
rs675640
rs61117627
ENCODE Clustering (Density)
DNaseI Cluster: 111
DNaseI Cluster: 123
DNaseI Cluster: 45
DNaseI Cluster: 52
DNaseI Cluster: 99
HEEpiC
HMEC
NHEK
SAEC
Th1

Supplementary Figure 12: Conditional analysis, posterior probability analysis, chromatin looping, and eQTL mapping in the *TYK2* locus.

(a) Conditional analysis was performed, adjusting for rs11085725. A residual effect was for the top SNP, rs2278442.

(b) Posterior probabilities distribution of variants in *TYK2* locus, identifying rs11085725 and rs35251378 as the most probable SNPs.

(c-e) The pairwise *D'* (**c**), r^2 (**d**), and haplotypes with their frequencies of the top and functional variants in the region are displayed (**e**).

(f) Circos plot mapping the zoom regional Manhattan plot of the imputed GWAS data for the *TYK2* association on Chromosome 19 (outer most layer). All SNPs with a $-\log_{10}$(p-value) <0.05 are shown in black, or colored based on r^2 (red: r^2>0.08; orange: r^2>0.06). The index SNP of the *TYK2* association, rs11085725, is indicated. The outer circle displays the chromosome coordinate of the *TYK2* risk locus highlighted in blue. Genes that are eQTLs or exhibit chromatin interaction by Hi-C in GM12878 Epstein-Barr virus (EBV)-transformed B lymphocytes are reported on the inner circles in green or orange font, respectively, and are shown as links colored green or orange, respectively, on the inner most layer.

(g) Sjögren-SNP rs2304256 is a previously characterized missense variant in *TYK2*. For details, see Supplementary Table 34.

(h) Sjögren-SNP rs11879191, positioned in an intronic region of *CDC37* downstream of *TYK2*, exhibits epigenetic promoter and enhancer marks in all immune cell types presented (rectangles). The intronic regulatory element engages a broad regulatory network including the promoters of several eQTLs including *ICAM5* and *CDC37* in monocytes, and *OLFM2* and *EIF3G* in the salivary gland. For details, see Supplementary Table 34.

(i) Sjögren-SNP rs2278442 is positioned in intronic region of *ICAM3* that is enriched with epigenetic promoter and enhancer marks across most immune cell types shown. Coalescence of chromatin interactions and eQTL data suggest that this regulatory region may also modulate the expression of *ICAM5*, *ICAM4*, *MRPL4*, *TYK2*, and *ICAM1*. For details, see Supplementary Table 34.

(j-n) EcholocatoR was used to identify, fine-map and annotate the *TYK2* region after specifying the index SNPs (indicated by red dotted line): rs2304256 (**j**), rs11879191 (**k**), rs2278442 (**l**), rs34953890 (**m**), and rs753859 (**n**). Additional SNPs with plausible function were identified and indicated by the dotted yellow lines.

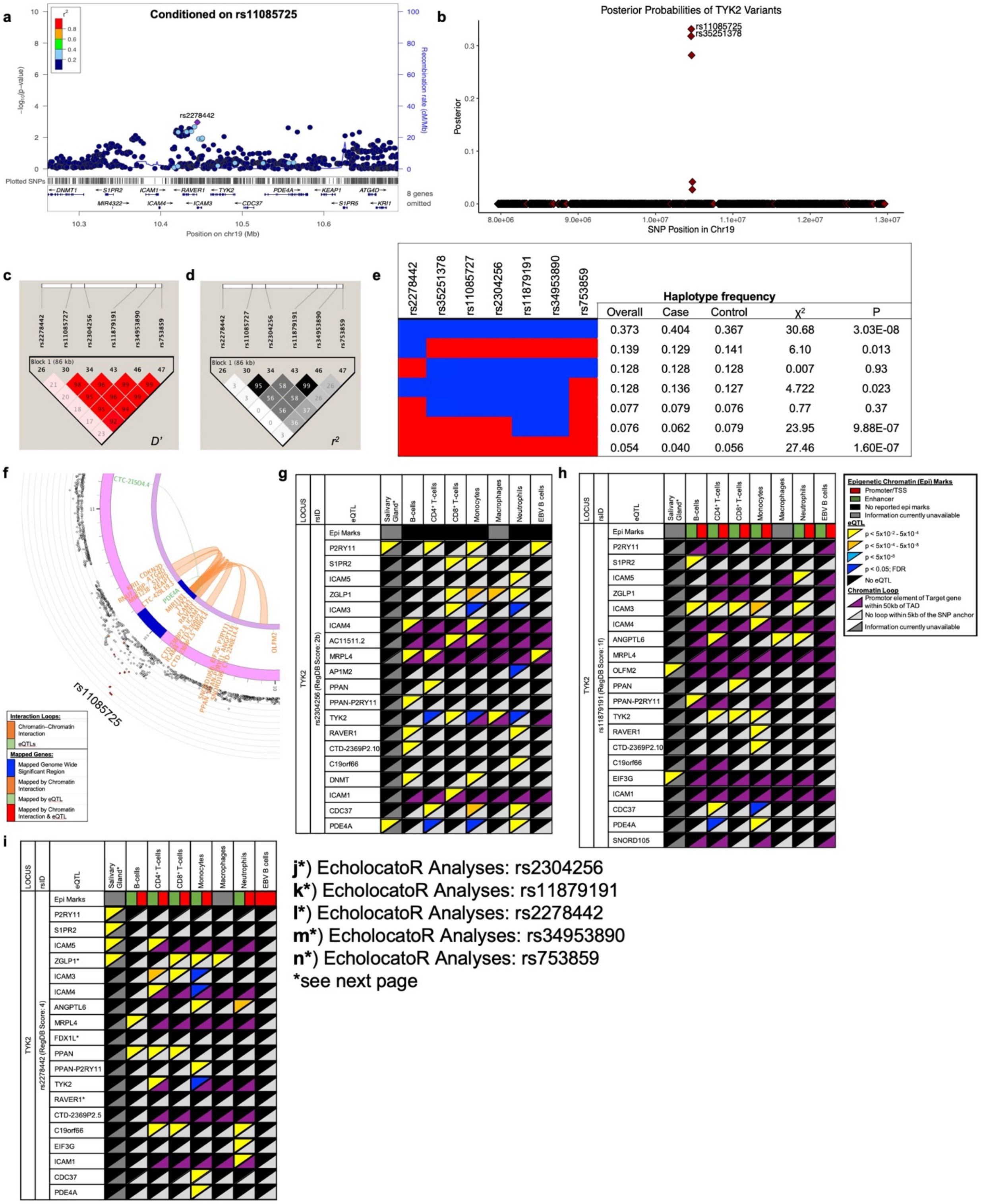

Overall	Case	Control	χ²	P
0.373	0.404	0.367	30.68	3.03E-08
0.139	0.129	0.141	6.10	0.013
0.128	0.128	0.128	0.007	0.93
0.128	0.136	0.127	4.722	0.023
0.077	0.079	0.076	0.77	0.37
0.076	0.062	0.079	23.95	9.88E-07
0.054	0.040	0.056	27.46	1.60E-07

j*) EcholocatoR Analyses: rs2304256
k*) EcholocatoR Analyses: rs11879191
l*) EcholocatoR Analyses: rs2278442
m*) EcholocatoR Analyses: rs34953890
n*) EcholocatoR Analyses: rs753859
*see next page

TYK2_rs2304256

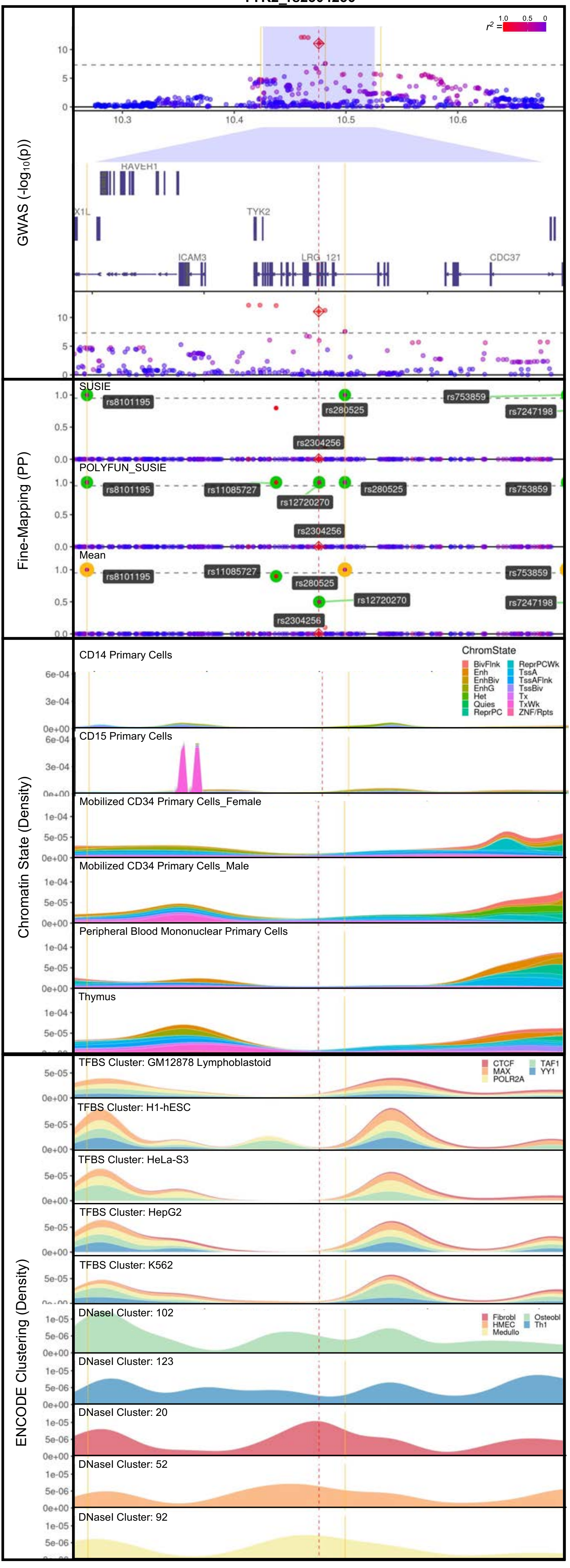

TYK2_rs11879191

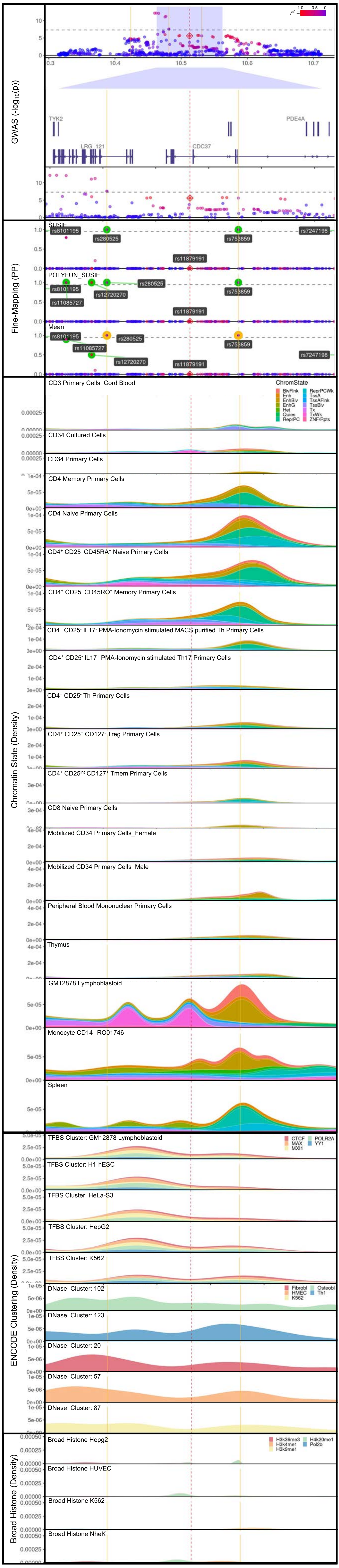

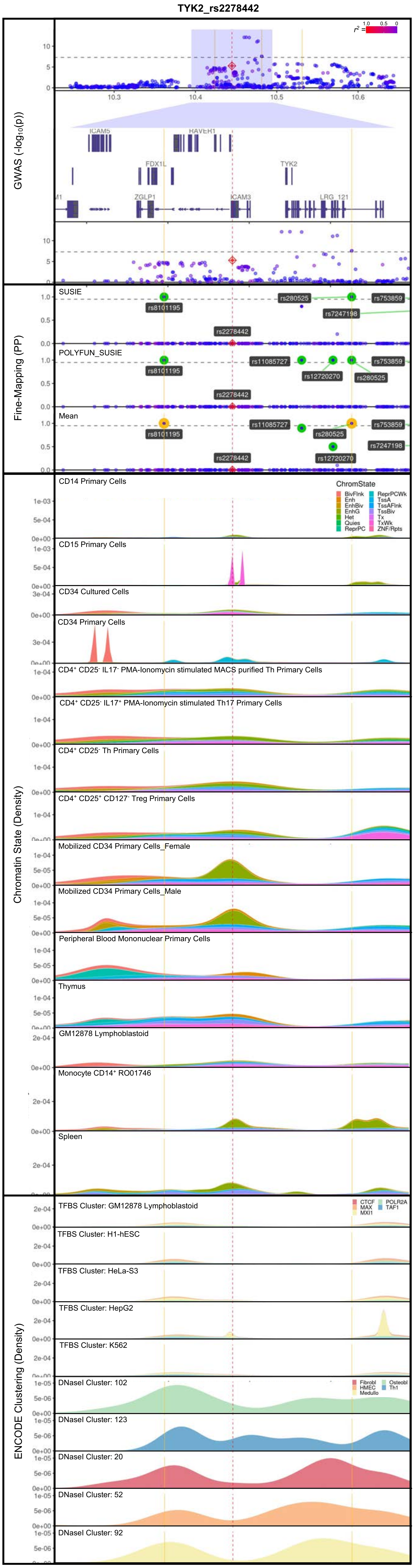

TYK2_rs2278442
GWAS (-log10(p))
ICAM5
RAVER1
FDX1L
TYK2
ZGLP1
ICAM3
LRG_121
Fine-Mapping (PP)
SUSIE
POLYFUN_SUSIE
Mean
rs8101195
rs2278442
rs280525
rs753859
rs7247198
rs11085727
rs12720270
Chromatin State (Density)
CD14 Primary Cells
ChromState
BivFlnk
Enh
EnhBiv
EnhG
Het
Quies
ReprPC
ReprPCWk
TssA
TssAFlnk
TssBiv
Tx
TxWk
ZNF/Rpts
CD15 Primary Cells
CD34 Cultured Cells
CD34 Primary Cells
CD4+ CD25- IL17- PMA-Ionomycin stimulated MACS purified Th Primary Cells
CD4+ CD25- IL17+ PMA-Ionomycin stimulated Th17 Primary Cells
CD4+ CD25- Th Primary Cells
CD4+ CD25+ CD127- Treg Primary Cells
Mobilized CD34 Primary Cells_Female
Mobilized CD34 Primary Cells_Male
Peripheral Blood Mononuclear Primary Cells
Thymus
GM12878 Lymphoblastoid
Monocyte CD14+ RO01746
Spleen
ENCODE Clustering (Density)
TFBS Cluster: GM12878 Lymphoblastoid
CTCF
MAX
MXI1
POLR2A
TAF1
TFBS Cluster: H1-hESC
TFBS Cluster: HeLa-S3
TFBS Cluster: HepG2
TFBS Cluster: K562
DNaseI Cluster: 102
Fibrobl
HMEC
Medullo
Osteobl
Th1
DNaseI Cluster: 123
DNaseI Cluster: 20
DNaseI Cluster: 52
DNaseI Cluster: 92

TYK2_rs34953890

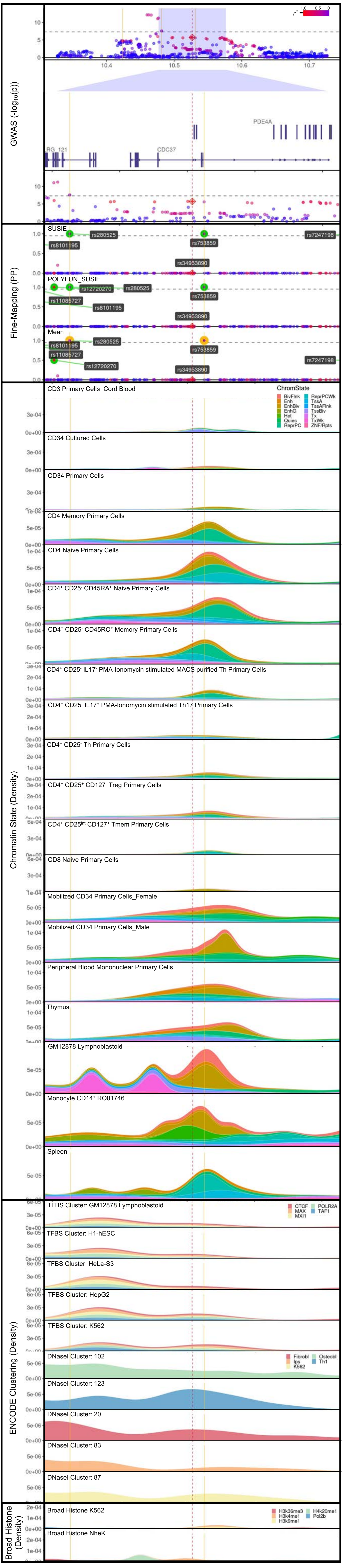

TYK2_rs753859

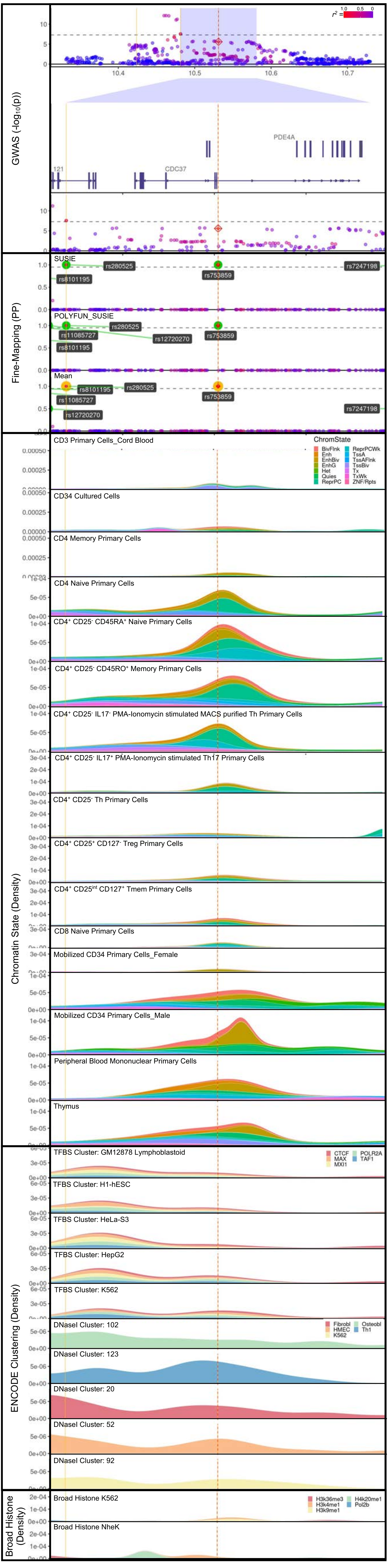

Supplementary Figure 13: Pathway, function, and disease enrichment of the index genes of each novel Sjögren's-associated risk locus and genes that share a predicted regulatory network with the index gene.
Ingenuity Pathway Analysis (IPA) Canonical Pathway and Disease and Function analyses were performed to assess the functional potential of the index gene, as well as genes that share a regulatory network with the index genes. Blue color indicates tested genes (top row) that were associated with specified cell functions, signaling pathways, or diseases (first two columns).

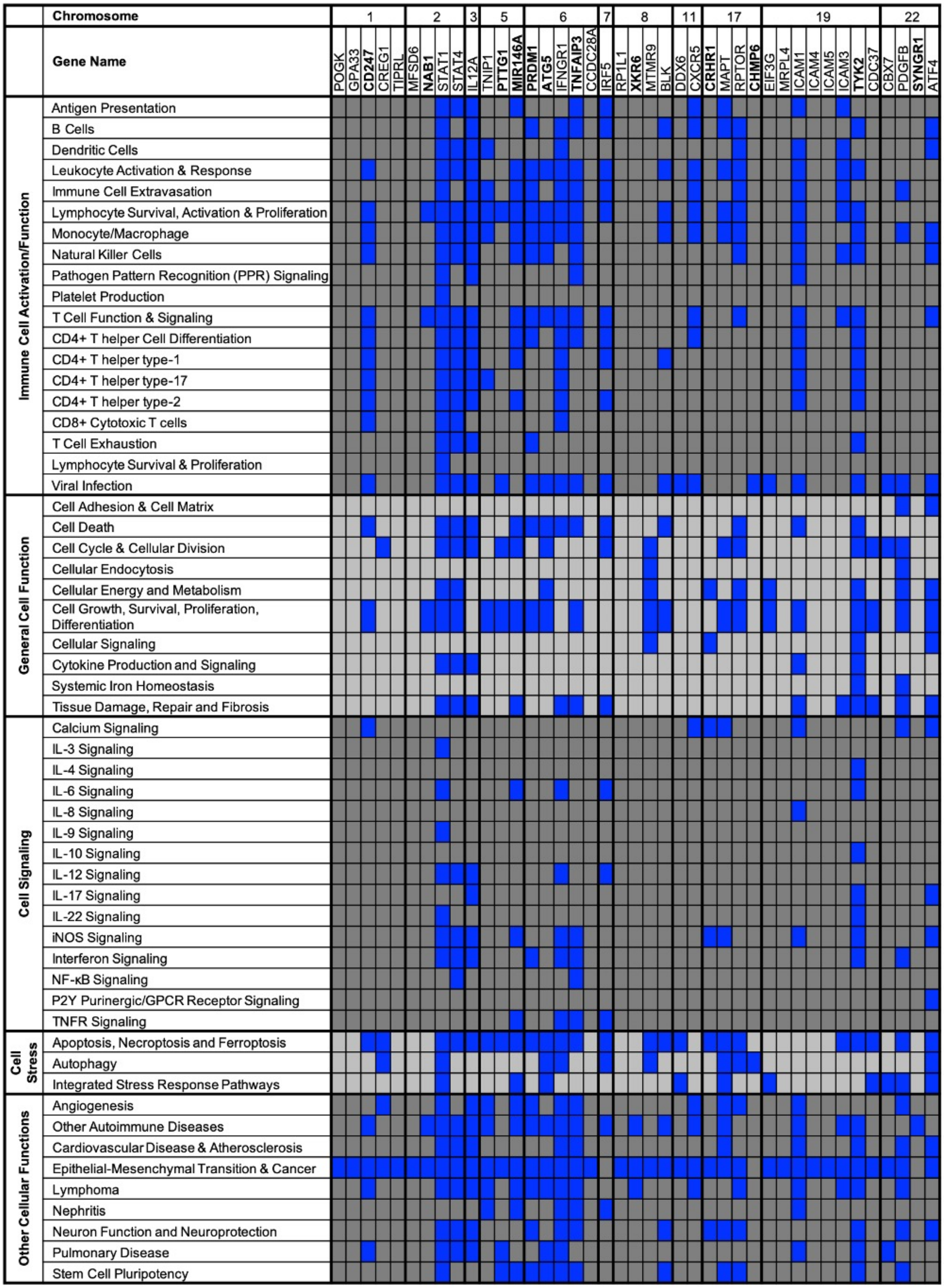
Chromosome
1
2
3
5
6
7
8
11
17
19
22
Gene Name
POGK
GPA33
CD247
CREG1
TIPRL
MFSD6
NAB1
STAT1
STAT4
IL12A
TNIP1
PTTG1
MIR146A
PRDM1
ATG5
IFNGR1
TNFAIP3
CCDC28A
IRF5
RP1L1
XKR6
MTMR9
BLK
DDX6
CXCR5
CRHR1
MAPT
RPTOR
CHMP6
EIF3G
MRPL4
ICAM1
ICAM4
ICAM5
ICAM3
TYK2
CDC37
CBX7
PDGFB
SYNGR1
ATF4
Immune Cell Activation/Function
Antigen Presentation
B Cells
Dendritic Cells
Leukocyte Activation & Response
Immune Cell Extravasation
Lymphocyte Survival, Activation & Proliferation
Monocyte/Macrophage
Natural Killer Cells
Pathogen Pattern Recognition (PPR) Signaling
Platelet Production
T Cell Function & Signaling
CD4+ T helper Cell Differentiation
CD4+ T helper type-1
CD4+ T helper type-17
CD4+ T helper type-2
CD8+ Cytotoxic T cells
T Cell Exhaustion
Lymphocyte Survival & Proliferation
Viral Infection
General Cell Function
Cell Adhesion & Cell Matrix
Cell Death
Cell Cycle & Cellular Division
Cellular Endocytosis
Cellular Energy and Metabolism
Cell Growth, Survival, Proliferation, Differentiation
Cellular Signaling
Cytokine Production and Signaling
Systemic Iron Homeostasis
Tissue Damage, Repair and Fibrosis
Cell Signaling
Calcium Signaling
IL-3 Signaling
IL-4 Signaling
IL-6 Signaling
IL-8 Signaling
IL-9 Signaling
IL-10 Signaling
IL-12 Signaling
IL-17 Signaling
IL-22 Signaling
iNOS Signaling
Interferon Signaling
NF-κB Signaling
P2Y Purinergic/GPCR Receptor Signaling
TNFR Signaling
Cell Stress
Apoptosis, Necroptosis and Ferroptosis
Autophagy
Integrated Stress Response Pathways
Other Cellular Functions
Angiogenesis
Other Autoimmune Diseases
Cardiovascular Disease & Atherosclerosis
Epithelial-Mesenchymal Transition & Cancer
Lymphoma
Nephritis
Neuron Function and Neuroprotection
Pulmonary Disease
Stem Cell Pluripotency
