# Supplementary material for: Genome-wide association study identifies Sjögren’s risk loci with functional implications in immune and glandular cells
Source: Nat Commun. 2022 Jul 27;13:4287. doi: 10.1038/s41467-022-30773-y (PMC9329286; doi:10.1038/s41467-022-30773-y)
Supplement: Supplementary file 2 — Description of Additional Supplementary Files [file 41467_2022_30773_MOESM2_ESM.pdf]

Description of Additional Supplementary Files

File Name: Supplementary Data 1
Description: Replication of previously established GWS regions outside of the HLA in Sjogren's disease.

File Name: Supplementary Data 2
Description: Summary of samples contributed by each collaborating institution/cohort

File Name: Supplementary Data 3
Description: Suggestive GWS regions in Sjogren's disease of European Ancestry

File Name: Supplementary Data 4
Description: Epigenetic Enrichment Analysis of GWS Sjögren-SNPs in different cells and tissues

File Name: Supplementary Data 5
Description: Association analysis in CRHR1 locus.

File Name: Supplementary Data 6
Description: Bayesian Analysis in CRHR1 locus.

File Name: Supplementary Data 7
Description: CRHR1 Blood Traits

File Name: Supplementary Data 8
Description: Association analysis in CD247 locus.

File Name: Supplementary Data 9
Description: Bayesian Analysis in CD247 locus.

File Name: Supplementary Data 10
Description: eQTL, Looping and Regulatory chromatin states for functional variants in CD247 locus.

File Name: Supplementary Data 11
Description: Association analysis in XKR6 locus.

File Name: Supplementary Data 12
Description: Bayesian Analysis in XKR6 locus.

File Name: Supplementary Data 13
Description: eQTL, Looping and Regulatory chromatin states for functional variants in XKR6 locus.

File Name: Supplementary Data 14

Description: Association analysis in SYNGR1 locus.

File Name: Supplementary Data 15
Description: Bayesian Analysis in SYNGR1 locus.

File Name: Supplementary Data 16
Description: eQTL, Looping and Regulatory chromatin states for functional variants in SYNGR1 locus.

File Name: Supplementary Data 17
Description: Association analysis in NAB1 locus.

File Name: Supplementary Data 18
Description: Bayesian Analysis in NAB1 locus.

File Name: Supplementary Data 19
Description: eQTL, Looping and Regulatory chromatin states for functional variants in NAB1 locus.

File Name: Supplementary Data 20
Description: Association analysis in CHMP6 locus.

File Name: Supplementary Data 21
Description: Bayesian Analysis in CHMP6 locus.

File Name: Supplementary Data 22
Description: eQTL, Looping and Regulatory chromatin states for functional variants in CHMP6 locus.

File Name: Supplementary Data 23
Description: Association analysis in PRDM1-ATG5 locus.

File Name: Supplementary Data 24
Description: Bayesian Analysis in PRDM1-ATG5 locus.

File Name: Supplementary Data 25
Description: eQTL, Looping and Regulatory chromatin states for functional variants in PRDM1-ATG5 locus.

File Name: Supplementary Data 26
Description: Association analysis in MIR146A-PTTG1 locus.

File Name: Supplementary Data 27
Description: Bayesian Analysis in MIR146A-PTTG1 locus.

File Name: Supplementary Data 28

Description: eQTL, Looping and Regulatory chromatin states for functional variants in MIR146A-PTTG1 locus.

File Name: Supplementary Data 29
Description: Association analysis in TNFAIP3 locus.

File Name: Supplementary Data 30
Description: Bayesian Analysis in TNFAIP3 locus.

File Name: Supplementary Data 31
Description: eQTL, Looping and Regulatory chromatin states for functional variants in TNFAIP3 locus.

File Name: Supplementary Data 32
Description: Association analysis in TYK2 locus.

File Name: Supplementary Data 33
Description: Bayesian Analysis in TYK2 locus.

File Name: Supplementary Data 34
Description: eQTL, Looping and Regulatory chromatin states for functional variants in TYK2 locus.
