# Supplementary material for: Genome-wide association study identifies Sjögren’s risk loci with functional implications in immune and glandular cells
Source: Nat Commun. 2022 Jul 27;13:4287. doi: 10.1038/s41467-022-30773-y (PMC9329286; doi:10.1038/s41467-022-30773-y)
Supplement: Supplementary file 4 — Reporting Summary [file 41467_2022_30773_MOESM4_ESM.pdf]

Corresponding author(s): Christopher J. Lessard, PhD

Last updated by author(s): Feb 15, 2022

Reporting Summary

Nature Portfolio wishes to improve the reproducibility of the work that we publish. This form provides structure for consistency and transparency in reporting. For further information on Nature Portfolio policies, see our Editorial Policies and the Editorial Policy Checklist.

Statistics

For all statistical analyses, confirm that the following items are present in the figure legend, table legend, main text, or Methods section.

n/a	Confirmed	
☐	☒	The exact sample size (*n*) for each experimental group/condition, given as a discrete number and unit of measurement
☐	☒	A statement on whether measurements were taken from distinct samples or whether the same sample was measured repeatedly
☐	☒	The statistical test(s) used AND whether they are one- or two-sided<br>*Only common tests should be described solely by name; describe more complex techniques in the Methods section.*
☐	☒	A description of all covariates tested
☒	☐	A description of any assumptions or corrections, such as tests of normality and adjustment for multiple comparisons
☐	☒	A full description of the statistical parameters including central tendency (e.g. means) or other basic estimates (e.g. regression coefficient) AND variation (e.g. standard deviation) or associated estimates of uncertainty (e.g. confidence intervals)
☐	☒	For null hypothesis testing, the test statistic (e.g. *F*, *t*, *r*) with confidence intervals, effect sizes, degrees of freedom and *P* value noted<br>*Give P values as exact values whenever suitable.*
☒	☐	For Bayesian analysis, information on the choice of priors and Markov chain Monte Carlo settings
☒	☐	For hierarchical and complex designs, identification of the appropriate level for tests and full reporting of outcomes
☐	☒	Estimates of effect sizes (e.g. Cohen's *d*, Pearson's *r*), indicating how they were calculated

Our web collection on statistics for biologists contains articles on many of the points above.

Software and code

Policy information about availability of computer code

Data collection	No software was used to collect data for this study.
Data analysis	R version 4.0.4, GenomeStudio (v2011. and v2.0), Common Workflow Language (CWL) Regulatory Element Locus Intersection (RELI) algorithm v0.90, PLINK v1.09, EIGENSTRAT (EIGENSOFT v7.2.1, released June 2017), SHAPEIT v4 algorithm, Minimac3 algorithm (released 2018-08-09), METAL (released 2011-03-25) LocusZoom, HAPLOVIEW v4.2, Trinculo v0.96, echolocatoR v0.1.2, PRSice2 v2.3.5, R package pROC v1.18.0, Cross-trait linkage disequilibrium score regression (LDSC) v1.0.1 command line tool , GREGOR.

For manuscripts utilizing custom algorithms or software that are central to the research but not yet described in published literature, software must be made available to editors and reviewers. We strongly encourage code deposition in a community repository (e.g. GitHub). See the Nature Portfolio guidelines for submitting code & software for further information.

Data

Policy information about availability of data

All manuscripts must include a data availability statement. This statement should provide the following information, where applicable:

- Accession codes, unique identifiers, or web links for publicly available datasets
- A description of any restrictions on data availability
- For clinical datasets or third party data, please ensure that the statement adheres to our policy

The genome-wide association summary statistics generated in this study have been deposited in the Databases of Genotypes and Phenotypes (dbGaP) under accession number: phs002723.v1.p1 [http://www.ncbi.nlm.nih.gov/projects/gap/cgi-bin/study.cgi?study_id=phs002723.v1.p1]. Individual-level genotype data for 1,199 subjects included in this study are available via dbGAP controlled access under accession number: phs002723.v1.p1 [http://www.ncbi.nlm.nih.gov/projects/gap/cgi-bin/study.cgi?study_id=phs002723.v1.p1]. Individual-level genotype data for 10,850 subjects are available via dbGAP controlled access under accession

numbers: phs000672.v1.p1 (n=735) [https://www.ncbi.nlm.nih.gov/projects/gap/cgi-bin/study.cgi?study_id=phs000672.v1.p1], phs000428.v2.p2 (n=8,519) [https://www.ncbi.nlm.nih.gov/projects/gap/cgi-bin/study.cgi?study_id=phs000428.v2.p2], phs000196.v3.p1 (n=995) [https://www.ncbi.nlm.nih.gov/projects/gap/cgi-bin/study.cgi?study_id=phs000196.v3.p1], phs000187.v1.p1 (n=602) [https://www.ncbi.nlm.nih.gov/projects/gap/cgi-bin/study.cgi?study_id=phs000187.v1.p1]. Remaining pre-existing individual-level genotype data were generated by coauthors and provided for specific use in this study and cannot be publicly shared per data use agreements. Contact the corresponding author (chris-lessard@omrf.org) with inquiries about accessing this pre-existing genotyping data.

All other data presented in this study was previously published and can be accessed by: Haplotype Reference Consortium panel version 1.1 (Michigan Imputation Server) [https://imputationserver.sph.umich.edu/], 1000 Genomes Project reference data [http://ftp.1000genomes.ebi.ac.uk/vol1/ftp/release/20130502/], EnhancerAtlas 2.0 database [http://www.enhanceratlas.org/], FUMA [http://fuma.ctglab.nl/], RegulomeDB [https://www.regulomedb.org/regulome-search/], Open Targets [https://www.opentargets.org], Haploreg [https://pubs.broadinstitute.org/mammals/haploreg/haploreg.php], GWAS Summary used in LDSC analysis [https://alkesgroup.broadinstitute.org/sumstats_formatted/?C=S;O=A], LD Score European data from the 1000 Genome Project [https://alkesgroup.broadinstitute.org/LDSCORE], 3D Genome Browser [http://3dgenome.fsm.northwestern.edu/chic.php], UCSC Genome Browser [http://genome.ucsc.edu/], QTLbase [http://mulinlab.tmu.edu.cn/qtlbase].

Field-specific reporting

Please select the one below that is the best fit for your research. If you are not sure, read the appropriate sections before making your selection.

☒ Life sciences ☐ Behavioural & social sciences ☐ Ecological, evolutionary & environmental sciences

For a reference copy of the document with all sections, see nature.com/documents/nr-reporting-summary-flat.pdf

Life sciences study design

All studies must disclose on these points even when the disclosure is negative.

Sample size	Sample size for the GWAS study was the sum of individual genotype data obtained from Sjögren's cases (n=3885) and population controls (n=23725) collected from the 25 individual cohorts and/or dbGaP as described in Supplementary Table 2; post-quality control sample size included n=3232 Sjögren's cases and n=17481 population controls. Polygenic Risk Score analyses was conducted in two parts: All Sjögren's cases v. population controls (3243 cases, 17464 controls) and Sjögren's cases that had reported anti-Ro autoantibody status v. population controls (1718 cases, 17440 controls). Genotype data on individuals with Sjögren's are limited, therefore all available Sjögren's cases of European ancestry that were available through the Sjögren's Genetics Network were used for this GWAS. Population controls were obtained from dbGaP to reach the accepted discovery ratio of 5 population controls to 1 case.
Data exclusions	Individual genotype data were excluded from the merged GWAS dataset and ImmunoChip dataset (see Figure 1a) if it had <95% call rate and excessive heterozygosity (>5 S.D. from the mean). PLINK was used to determine relatedness within the remaining samples using identity-by-decent (IBD) estimates. One individual from each pair was removed if the proportion of the alleles that shared IBD was >0.4.
Replication	All available genotype and ImmunoChIP data were incorporated into the GWAS and meta-analysis, respectively, to maximize discovery power. Effect sizes for each reported association signal are presented in Table 1 and the Supplementary Materials.
Randomization	Quality control was performed on each dataset (organized by sequencing technology used; see Figure 1A, Supplementary Table 2) prior to merging datasets for GWAS and meta-analysis. Genotyped individuals were randomly divided into training and testing datasets for the polygenic risk score calculation (See above and Figure 1A).
Blinding	Analysts were not blinded for this study because the case-control status of the genotyped subjects was essential for performing the case-control GWAS analysis and PRS calculation.

Reporting for specific materials, systems and methods

We require information from authors about some types of materials, experimental systems and methods used in many studies. Here, indicate whether each material, system or method listed is relevant to your study. If you are not sure if a list item applies to your research, read the appropriate section before selecting a response.

Materials & experimental systems

n/a	Involved in the study	
☒	☐	Antibodies
☒	☐	Eukaryotic cell lines
☒	☐	Palaeontology and archaeology
☒	☐	Animals and other organisms
☐	☒	Human research participants
☒	☐	Clinical data
☒	☐	Dual use research of concern

Methods

n/a	Involved in the study	
☒	☐	ChIP-seq
☒	☐	Flow cytometry
☒	☐	MRI-based neuroimaging

Human research participants

Policy information about studies involving human research participants

Population characteristics	This is a GWAS and meta-analysis of Sjögren's cases of European ancestry from the United States of America, Australia, Austria, France, Germany, Hungary, Italy, Norway, Spain, Sweden, Switzerland, and United Kingdom. For many of the Sjögren's case cohorts, demographic information was provided with Sjögren's diagnosis according to American-European Consensus Group (AECG) criteria. However, information on covariants such as age, sex, disease status, etc., were unavailable for most of the genotyped population control individuals obtained from dbGaP. For this reason, we were unable to analyze reported covariates. Instead, principal component analysis (PCA) was used to identify and remove outliers defined as having S.D. > 6 (see Supplementary Figure 1). Sex was determined from the genotype data post-quality control (see Supplementary Table 2).
Recruitment	This GWAS did not actively recruit subjects. Rather, recruitment of Sjögren's cases (and some matched controls) was conducted at each of the sites from which the 25 cohorts were obtained (see Supplementary Table 2). Recruitment strategies varied by site. The majority of the population controls were obtained through dbGaP.
Ethics oversight	Written informed consent was obtained in accordance with the Institutional Review Boards of each collaborating site as confirmed by the site Institutional Certification or dbGaP. All study protocols and informed consent documents from outside institutions were reviewed and approved by the OMRF Institutional Review Board where this GWAS and meta-analysis study was conducted. Similarly, the use of dbGaP data was reviewed and approved by the OMRF Institutional Review Board.

Note that full information on the approval of the study protocol must also be provided in the manuscript.
